# Cardiolipin coordinates inflammatory metabolic reprogramming through regulation of Complex II disassembly and degradation

**DOI:** 10.1126/sciadv.ade8701

**Published:** 2023-02-03

**Authors:** Mack B. Reynolds, Hanna S. Hong, Britton C Michmerhuizen, Anna-Lisa E. Lawrence, Li Zhang, Jason S. Knight, Costas A. Lyssiotis, Basel H. Abuaita, Mary X. O’Riordan

**Affiliations:** ^1^ Department of Microbiology and Immunology, University of Michigan Medical School, Ann Arbor, MI 48109, USA.; ^2^ Department of Molecular and Integrative Physiology, University of Michigan Medical School, Ann Arbor, MI 48109, USA.; ^3^ Department of Internal Medicine, Division of Rheumatology, University of Michigan, Ann Arbor, MI 48109, USA.

## Abstract

Macrophage metabolic plasticity enables repurposing of electron transport from energy generation to inflammation and host defense. Altered respiratory complex II function has been implicated in cancer, diabetes, and inflammation, but regulatory mechanisms are incompletely understood. Here, we show that macrophage inflammatory activation triggers Complex II disassembly and succinate dehydrogenase subunit B loss through sequestration and selective mitophagy. Mitochondrial fission supported lipopolysaccharide-stimulated succinate dehydrogenase subunit B degradation but not sequestration. We hypothesized that this Complex II regulatory mechanism might be coordinated by the mitochondrial phospholipid cardiolipin. Cardiolipin synthase knockdown prevented lipopolysaccharide-induced metabolic remodeling and Complex II disassembly, sequestration, and degradation. Cardiolipin-depleted macrophages were defective in lipopolysaccharide-induced pro-inflammatory cytokine production, a phenotype partially rescued by Complex II inhibition. Thus, cardiolipin acts as a critical organizer of inflammatory metabolic remodeling.

## Teaser

Lipids choose who stays and who loses

## INTRODUCTION

Metabolic plasticity is a central feature of immunity (*1*, *2*). Immune cells respond to diverse physiological cues, including infectious and sterile inflammatory stimuli, by eliciting distinct metabolic programs. While it has long been appreciated that these metabolic changes reshape cellular bioenergetics, more recent studies indicate that context-driven metabolic remodeling defines immune cell fate and function. Although metabolic plasticity is common among immune cells, the regulatory mechanisms and consequences of these metabolic changes are highly specialized in different cell types (*3*–*5*).

Metabolic remodeling in macrophages contributes to the initiation and resolution of inflammation in multiple human diseases, including sepsis, infection, inflammatory disease, and autoimmune disease (*3*, *6*–*8*). Furthermore, a variety of metabolic determinants of inflammatory signaling have been identified in macrophages. Control of oxidative phosphorylation, particularly through modulation of respiratory chain function, is critical for inflammatory programming. Recent work has highlighted respiratory complex II [Complex II; succinate dehydrogenase (SDH)], which functions uniquely at the interface of the electron transport chain (ETC) and the tricarboxylic acid (TCA) cycle, as a major player in macrophage metabolic remodeling and inflammatory programming (*9*–*13*). While ample evidence connects Complex II and its substrate succinate to macrophage inflammatory programming, the regulatory mechanisms governing Complex II activity in macrophages remain to be defined.

Prior work points to accumulation and oxidation of succinate as critical for macrophage inflammatory programming. Inflammatory accumulation of succinate has been attributed to increased flux through the gamma-aminobutyric acid (GABA) shunt (*10*). Succinate produced through this mechanism enhances lipopolysaccharide (LPS)-stimulated inflammatory signaling through oxidative stress–dependent activation of the transcription factor hypoxia inducible factor 1 (HIF-1), which increases the production of the immature form of the pro-inflammatory cytokine interleukin-1β (pro–IL-1β). Nevertheless, sustained inhibition of Complex II, which results in succinate accumulation, suppresses inflammatory signaling. Notably, deletion of one of the four subunits of Complex II, SDHB, in macrophages leads to a hypo-inflammatory phenotype, as indicated by a defect in LPS-induced pro–IL-1β (*9*). Thus, current models support the idea that Complex II is required for the inflammatory effect of succinate accumulation. Costimulation of macrophages with LPS and interferon gamma (IFN-γ) results in loss of Complex II in a nitric oxide (NO)–dependent manner (*14*). Furthermore, LPS-induced itaconate can serve as a reversible competitive inhibitor of Complex II (*15*). Last, acute bacterial infection of macrophages results in increased Complex II activity within the first 2 hours (*12*). Overall, the current literature predicts that precise and dynamic control of Complex II activity during inflammatory macrophage activation may be important, but the regulatory mechanisms controlling activity and stability of Complex II during the course of macrophage inflammatory programming are not well defined.

The supramolecular organization of the respiratory complexes depends on phospholipids present in the inner mitochondrial membrane (*16*, *17*). In particular, the mitochondrial phospholipid cardiolipin (CL) scaffolds the respiratory complexes in supercomplexes and enhances their function. Furthermore, human diseases caused by defects in CL metabolism, such as Barth syndrome, are associated with defects in RC organization and function across multiple cell types (*18*). While CL has been linked to RC function, little is known about how CL may control inflammatory modulation of RC activity in macrophages. Here, we identify the regulation of SDHB as a key step in LPS-induced respiratory chain remodeling and demonstrate a critical role for CL in enabling Complex II disassembly and SDHB degradation. Our findings point to CL as an architect of macrophage metabolic remodeling that repurposes the respiratory chain toward inflammation.

## RESULTS

### Macrophage inflammatory activation destabilizes Complex II subunit SDHB

**Fig. 1.**
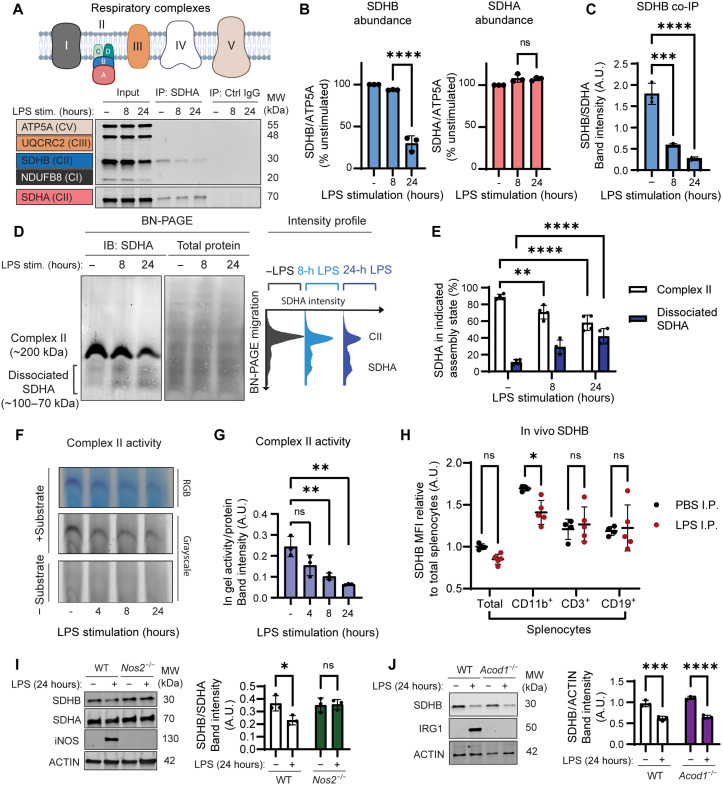
Macrophage LPS stimulation destabilizes Complex II. (**A**) Illustration of the five major respiratory complexes. Immortalized bone marrow–derived macrophages (iBMDMs) were synchronously stimulated with out without LPS (200 ng/ml) for 8 or 24 hours, and SDHA or control immunoglobulin G (IgG) was immunoprecipitated. SDS–polyacrylamide gel electrophoresis (PAGE) and IB analysis of ATP5A (Complex V adenosine 5´-triphosphate synthase F1 subunit alpha), UQCRC2 (ubiquinone Complex III cytochrome b-c1 complex subunit 2), SDHA, SHDB, and NDUFB8 (Complex I NADH dehydrogenase ubiquinone beta subcomplex subunit 8). (**B**) Quantification of SDHA and SDHB subunit abundance relative to ATP5A signal. (**C**) Quantification of immunoprecipitated SDHB relative to SDHA. Fluorescence measurements are reported in arbitrary units (A.U.). (**D**) Blue native (BN)–PAGE and SDHA IB analysis from iBMDM stimulated with or withoutLPS for 8 or 24 hours. Intensity profile of the SDHA blot showing Complex II–associated SDHA (~200 kDa) and dissociated SDHA (~100 to 70 kDa). (**E**) Percentage of SDHA in Complex II and dissociated SDHA populations. (**F**) Clear native (CN)–PAGE and SDH in-gel activity assay from iBMDM stimulated with out without LPS for 4, 8, or 24 hours. (**G**) SDH in-gel activity assay normalized to sample protein content. (**H**) Flow cytometric analysis of SDHB levels in total, CD3^+^, CD19^+^, and CD11b^+^ splenocytes from mice injected intraperitoneally (I.P.) with LPS (20 mg/kg) or phosphate-buffered saline (PBS). The mean fluorescence intensity (MFI) within each subset was plotted as a fold difference compared to the PBS-injected total splenocyte MFI. SDS-PAGE and IB analysis of SDHA, SDHB, IRG1 (immune-responsive gene 1), and iNOS (inducible NO synthase) from *Nos2*^−/−^ (**I**) and *Acod1*^−/−^ (**J**) BMDM stimulated with or without LPS for 24 hours. Graphs presented as the mean of *n* ≥ 3 independent experiments with SD error bars. *P* values were calculated using an unpaired *t* test or one-way analysis of variance (ANOVA) with Tukey’s posttest. *P < 0.05; **P < 0.01; ***P < 0.001; ****P < 0.0001. (A) was adapted from BioRender.com assets. co-IP, coimmunoprecipitation; MW, molecular weight; ns, not significant; WT, wild type.

Activation of Toll-like receptor 4 (TLR4) with LPS triggers profound metabolic remodeling in macrophages, which culminates in adenosine triphosphate (ATP) production by aerobic glycolysis at the expense of oxidative phosphorylation (*3*, *7*, *13*). In agreement with prior studies, stimulation of murine macrophages with LPS led to decreased oxygen consumption rate and increased extracellular acidification rate as early as 6 hours after treatment, shown by the Seahorse extracellular flux (XF) assay (fig. S1, A and B) (*6*, *19*, *20*). We therefore predicted that LPS stimulation might alter the relative abundance or assembly of the RCs, as has been shown for costimulation of macrophages with LPS and IFN-γ (*14*). To test this hypothesis, we performed immunoblot (IB) analysis of a panel of RC subunits in macrophages stimulated with LPS. RC subunit analysis revealed selective depletion of respiratory complex II (Complex II) subunit SDH subunit B (SDHB), but not subunit A (SDHA), at 24 hours after LPS stimulation (Fig. 1, A and B). Furthermore, Complex I NADH (reduced form of nicotinamide adenine dinucleotide) dehydrogenase ubiquinone beta subcomplex subunit 8 (NDUFB8) was decreased, while Complex III cytochrome b-c1 complex subunit 2 (UQCRC2) and Complex V ATP synthase F1 subunit alpha (ATP5A) were unchanged (fig. S2). Loss of SDHB was triggered by LPS in both immortalized and primary bone marrow–derived murine macrophages (fig. S3A). Furthermore, we found that Complex II activity was regulated in a similar fashion in primary human monocyte–derived macrophages (hMDMs), since SDHB, but not SDHA, was decreased by LPS stimulation, resulting in loss of Complex II activity by 24 hours after LPS stimulation (fig. S3, B and C). Our observation that Complex II is disrupted during LPS stimulation is consistent with earlier studies demonstrating that inflammatory macrophage activation triggers the inhibition of Complex II and the accumulation of the Complex II substrate succinate (*10*, *14*, *15*). Thus, Complex II disruption likely represents a conserved route to remodel the TCA cycle and complement succinate accumulation via the GABA shunt during LPS stimulation.

To investigate how LPS treatment affects the assembly of Complex II, we immunoprecipitated SDHA and measured coimmunoprecipitation of other representative RC subunits (Fig. 1, A and C). We found that, among the tested RC subunits, only SDHB coimmunoprecipitated with SDHA under unstimulated conditions, supporting a known phenomenon whereby Complex II rarely forms supercomplexes with other respiratory complexse (*21*). LPS stimulation triggered dissociation of SDHA and SDHB as early as 8 hours after LPS stimulation. In support of this, blue native–polyacrylamide gel electrophoresis (BN-PAGE) and IB analysis of SDHA 8 and 24 hours after LPS stimulation showed a decrease in native Complex II and a corresponding increase in Complex II–dissociated SDHA, which may represent SDHA monomer or SDHA bound to other proteins such as assembly factors (Fig. 1, D and E). Notably, the assemblies of Complex III, Complex IV, and Complex V were not perturbed by LPS stimulation (fig. S4). Thus, macrophage LPS stimulation alone seems to disrupt the respiratory complexes to a lesser extent than costimulation with LPS and IFN-γ (*14*). The disassembly of Complex II correlated with decreased enzymatic activity, as indicated by an in-gel activity assay of Complex II SDH activity (Fig. 1, F and G, and fig. S4C). Last, we found that pharmacological inhibition of Complex II was sufficient to stimulate glycolysis in macrophages (fig. S1C). Thus, we propose that Complex II disassembly and instability lead to functional inactivation, contributing to defective respiration and increased glycolysis during macrophage inflammatory activation.

Once we established the pattern of LPS-induced Complex II disassembly and instability in vitro, we sought to determine whether the phenomenon could occur in vivo. To accomplish this, we used a murine intraperitoneal endotoxemia model, injecting animals with LPS (20 mg/kg) and harvesting spleens at 24 hours after treatment. We measured intracellular SDHB levels by flow cytometry in different splenocyte cell lineages (Fig. 1H and fig. S5). Loss of SDHB occurred during endotoxemia in myeloid (cluster of differentiation [CD]11b^+^) but not lymphoid splenocytes (CD3^+^ or CD19^+^). Last, we tested if Complex II regulation occurred during infection with the Gram-negative bacterial pathogen *Salmonella enterica* serovar Typhimurium (STM). We found that STM infection led to comparable levels of Complex II disassembly as LPS, even at lower multiplicities of infection (MOIs) (fig. S6A). However, STM infection did not trigger the same extent of SDHB loss as LPS alone (fig. S6B). Two metabolites, NO, produced by inducible NO synthase (iNOS), and itaconate, produced by immune-responsive gene 1 (IRG1), have been identified as critical regulators of Complex II activity during inflammatory macrophage activation (*14*, *15*). Thus, we tested whether selective loss of SDHB during LPS stimulation could be attributed to iNOS or IRG1 by measuring SDHB levels and Complex II assembly in LPS-stimulated *Nos2*^−/−^ and *Acod1*^−/−^ macrophages, respectively (Fig. 1, I and J, and fig. S7). IRG1 was dispensable for LPS-induced loss of SDHB and Complex II disassembly, consistent with the model that itaconate is a competitive and reversible inhibitor of Complex II. Notably, iNOS was necessary for Complex II disassembly and loss of SDHB, as previously shown for costimulation of macrophages with LPS and IFN-γ (*14*).

### Complex II subunit SDHB is sequestered and degraded through selective mitophagy during macrophage inflammatory activation

**Fig. 2.**
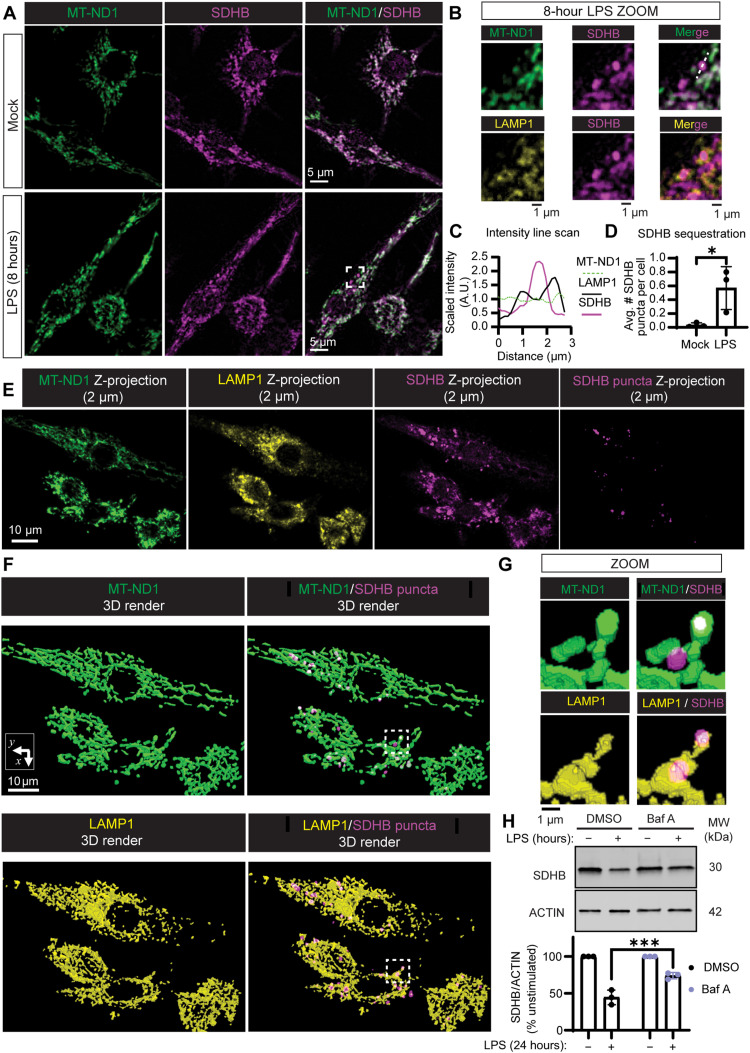
SDHB is delivered to an endolysosomal compartment for degradation during LPS stimulation. (**A**) Representative confocal fluorescence micrographs from iBMDM stimulated with or without LPS (200 ng/ml) for 8 hours and subjected to immunofluorescence labeling of SDHB and MT-ND1. (**B**) Magnified region from (A) and fig. S8B highlighting sequestration of SDHB signal into puncta, distinct from MT-ND1 signal and enclosed in a LAMP1-positive compartment. (**C**) Representative line scan from SDHB puncta in (B) where pixel intensity (A.U.) is scaled to the mean intensity across the sampled region. (**D**) Quantification of the average SDHB puncta per cell from (A) with CellProfiler, as highlighted in fig. S8 (C and D). (**E**) Representative maximal intensity Z-projections of 10 optical sections (2 μm), from MT-ND1, LAMP1, and SDHB immunostain in iBMDM stimulated with LPS. LPS-induced SDHB puncta are emphasized by thresholding the signal of mitochondrial network SDHB (magenta). (**F**) Three-dimensional render from (E) using the Allen Cell & Structure Segmenter Napari plugin. (**G**) Magnified regions highlight SDHB puncta localization within the mitochondrial network (MT-ND1 objects) and LAMP1 spherical structures. (**H**) SDS-PAGE and IB analysis of SDHB levels from iBMDM costimulated with or without LPS and bafilomycin A1 (Baf A, 100 μM) or vehicle control [dimethyl sulfoxide (DMSO)] quantified as the percentage of the unstimulated condition within each treatment condition. Graphs presented as the mean of *n* = 3 independent experiments with SD error bars. For image analysis, the mean of ~100 cells per condition is reported for each experiment. *P* values were calculated using an unpaired *t* test or two-way ANOVA with Sidak’s posttest. *P < 0.05; ***P < 0.001.

Selective loss of Complex II subunit SDHB, but not SDHA, upon LPS stimulation could be achieved by a variety of mechanisms, including gene expression and proteolytic pathways. To investigate SDHB protein-level regulation, we first explored the subcellular localization of Complex II during LPS stimulation. We visualized SDHA and SDHB subcellular localization by immunofluorescence staining and high-resolution confocal fluorescence microscopy, counterstaining with Complex I mitochondrial NADH–ubiquinone oxidoreductase chain 1 (MT-ND1) and lysosomal-associated membrane protein 1 (LAMP1) (Fig. 2A and fig. S8, A and B). At 8 hours after LPS stimulation, when we observed Complex II disassembly but not loss of SDHA or SDHB subunit abundance, we noted the sequestration of SDHB into puncta that were distinct from the main mitochondrial network as defined by MT-ND1 immunofluorescence (Fig. 2, B to D, and fig. S8, C and D). In contrast, SDHA remained ubiquitous across the mitochondrial network (fig. S8A). There was selective delivery of SDHB to the LAMP1-positive vesicular network (Fig. 2, B and C, and fig. S8B). Optical sectioning and three-dimensional (3D) rendering of confocal immunofluorescence micrographs revealed two subsets of SDHB puncta: one subset localized within spherical LAMP1 structures, while the other subset was retained within the mitochondrial network (Fig. 2, E to G). These results suggest that, upon LPS treatment, SDHB is locally sequestered within the mitochondrial network and then delivered to an endolysosomal compartment. To test whether endolysosomal degradation could contribute to LPS-induced loss of SDHB, we treated cells with LPS in the absence or presence of the vacuolar adenosine triphosphatase inhibitor bafilomycin A1 (Baf A1) and measured SDHB levels by IB (Fig. 2H). SDHB was not as efficiently lost in response to LPS when Baf A was present, compared to vehicle control, suggesting that SDHB can be degraded through delivery to the endolysosomal compartment.

**Fig. 3.**
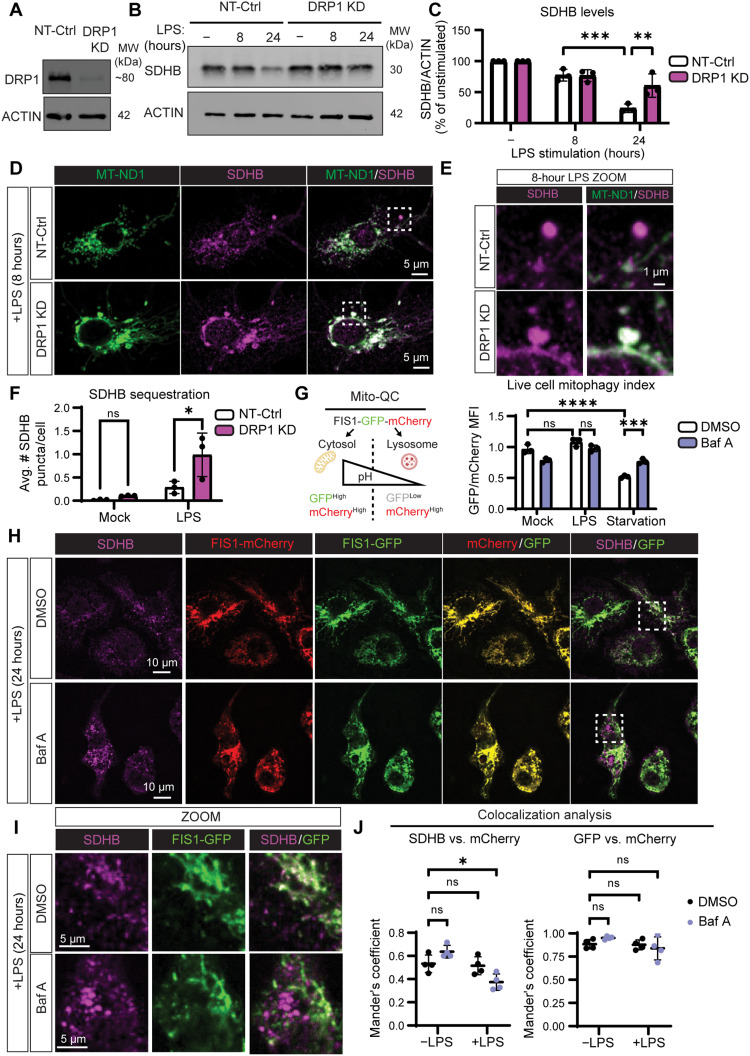
SDHB is degraded by selective mitophagy during LPS stimulation. (**A**) SDS-PAGE and IB analysis of DRP1 from iBMDM expressing nontarget control (NT-Control) or dynamin-related protein 1 (*Drp1*)–targeted short hairpin RNA (shRNA) (DRP1 KD). (**B**) SDS-PAGE and IB analysis of SDHB from NT-Control and DRP1 KD iBMDM stimulated with or without LPS (200 ng/ml) for 8 or 24 hours. (**C**) Quantification of (B) as the percentage of the unstimulated condition SDHB normalized to ACTIN. (**D**) Representative confocal SDHB and MT-ND1 immunofluorescence micrographs from NT-Control and DRP1 KD iBMDM stimulated with LPS for 8 hours. (**E**) Magnified region from (D). (**F**) Quantification of the average number of SDHB puncta per cell from (D) and fig. S9. (**G**) Illustration of the Mito-QC mitophagy reporter system. Ratio of green fluorescent protein (GFP) to mCherry MFI during live cell flow cytometric analysis of Mito-QC BMDM stimulated with LPS or starved (20% medium in PBS) for 24 hours in the presence of 100 nM bafilomycin A or vehicle control (DMSO). (**H**) Representative confocal SDHB immunofluorescence micrographs from Mito-QC BMDM costimulated with LPS and bafilomycin A or DMSO for 24 hours. (**I**) Magnified region from (H). (**J**) Colocalization of SDHB or GFP with mCherry using Mander’s coefficient. Graphs presented as the mean of *n* ≥ 3 independent experiments with SD error bars. The mean of ~100 cells per condition is reported for each experiment. *P* values were calculated using an unpaired *t* test or two-way ANOVA with Sidak’s posttest. *P < 0.05; **P < 0.01; ***P < 0.001; ****P < 0.0001. (G) was adapted from BioRender.com assets.

Delivery of mitochondrial components to the endolysosomal network can occur during mitochondrial autophagy (mitophagy), so we reasoned that the decrease in SDHB protein abundance might be mediated by mitophagy. During mitophagy, dynamin-related protein (DRP1)–dependent mitochondrial fission generates small (~1 μm^3^), fragmented mitochondria, which can be recognized by the autophagy machinery and captured for disposal (*22*). Thus, we predicted that DRP1 depletion would inhibit LPS-induced SDHB delivery to a LAMP1-positive compartment. We therefore stimulated DRP1 knockdown (KD) macrophages (Fig. 3A) (*23*) with LPS and measured SDHB levels by IB (Fig. 3, B and C). LPS-stimulated DRP1 KD macrophages failed to degrade SDHB compared to cells expressing nontarget control (NT-Control) short hairpin RNA (shRNA). In addition, we tracked SDHB subcellular localization by immunofluorescence labeling and confocal microscopy (Fig. 3D and fig. S9). We observed more prominent SDHB puncta formation in LPS-stimulated DRP1 KD macrophages, but these puncta remained associated with the mitochondrial network (Fig. 3, E and F). We conclude that DRP1 KD macrophages are capable of sequestering SDHB but infer that they cannot release SDHB puncta from the mitochondrial network for degradation.

Classically, mitophagy involves bulk organelle turnover, yet the route of SDHB degradation appears to exhibit selectivity since SDHA is not degraded (*24*). Thus, we tested whether enhanced bulk mitophagy contributes to SDHB loss during macrophage LPS stimulation. To accomplish this, we derived macrophages from Mito-QC mice that use dual green fluorescent protein (GFP) and mCherry-tagged outer mitochondrial membrane protein FIS1 to assess cytosolic versus endolysosomal localization of mitochondria (*25*). In principle, GFP fluorescence is quenched more rapidly than mCherry fluorescence when the mitochondria enter acidic conditions, such as the lysosomal compartment. We compared the ratio of GFP:mCherry intensity by flow cytometry in live Mito-QC macrophages stimulated with LPS, starvation conditions, or fresh medium in the presence of Baf A or vehicle control [dimethyl sulfoxide (DMSO)] (Fig. 3G). We found that starvation, which triggers mitophagy broadly (*26*), but not LPS stimulation, led to a decrease in the ratio of GFP:mCherry. Starvation-induced loss of GFP fluorescence was abrogated by Baf A treatment, which inhibits lysosomal acidification, validating the reporter system. We conclude that LPS stimulation does not trigger bulk mitophagy in macrophages within the first 24 hours of stimulation. With this knowledge, we performed a fixed cell immunofluorescence assay (IFA) against SDHB in 24-hour mock or LPS-stimulated Mito-QC macrophages in the presence of Baf A or vehicle control (DMSO) to assess selective mitophagy of SDHB (Fig. 3H and fig. S10A). We found that Baf A both inhibited the loss of SDHB (fig. S10, B and C) and resulted in vacuolar accumulation of SDHB outside the mitochondrial network, which could be quantified as a decrease in colocalization of SDHB with mCherry (Fig. 3, I and J). Notably, the colocalization of GFP and mCherry, another measurement of mitophagy in the Mito-QC system, was not altered by LPS stimulation relative to mock-treated cells. These data are consistent with a model where specific mitochondrial cargo, such as SDHB, can be selectively packaged and delivered for mitophagy during macrophage inflammatory activation.

While our data support selective mitophagy of SDHB, we also tested whether differences in *Sdhb* transcript could account for differences in protein levels. We stimulated macrophages with LPS and measured expression of *Sdha*, *Sdhb*, *Sdhc*, and *Sdhd* by reverse transcription quantitative polymerase chain reaction (RT-qPCR) (fig. S11). We found that LPS stimulation did not significantly decrease the transcript levels of Complex II subunits *Sdha*, *Sdhb*, or *Sdhc* but led to a ~50% reduction in *Sdhd* transcript. We conclude that transcript-level differences do not directly account for the selective loss of SDHB, but decreased expression of *Sdhd* may contribute indirectly. Together, our data support Complex II disassembly, followed by SDHB sequestration and degradation, as a functional consequence of LPS-induced macrophage innate immune signaling.

### CL licenses metabolic remodeling during macrophage inflammatory activation

**Fig. 4.**
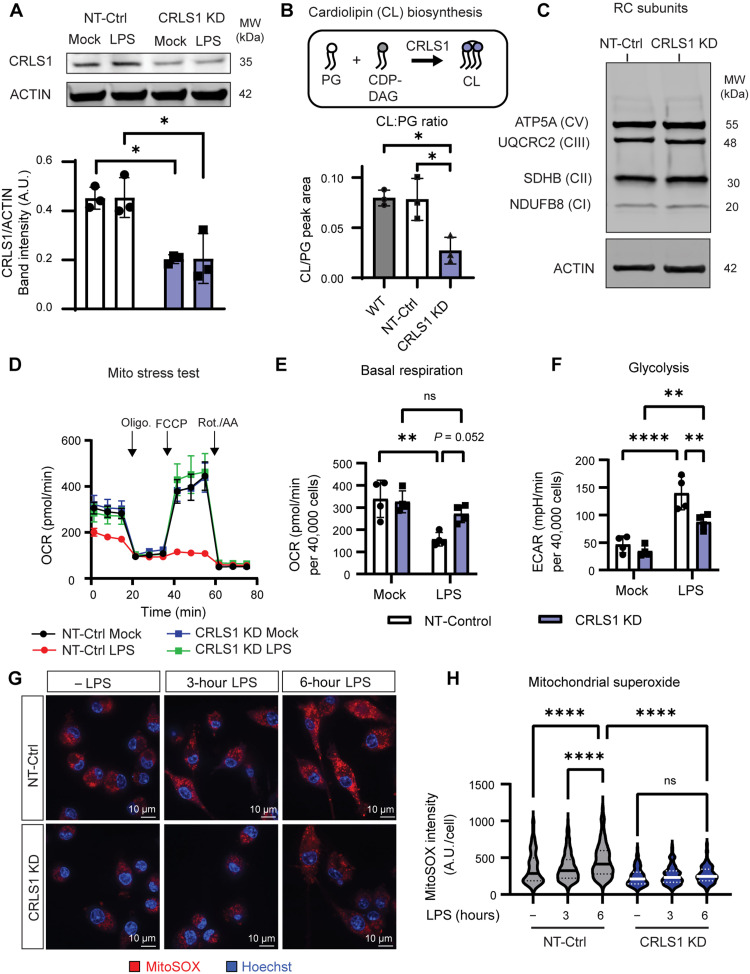
Cardiolipin biosynthesis licenses metabolic remodeling during macrophage LPS stimulation. (**A**) SDS-PAGE and IB analysis of CRLS1 from iBMDM expressing NT-Control or cardiolipin synthase (*Crls1*)-targeted shRNA (CRLS1 KD) stimulated with out without LPS (200 ng/ml) for 6 hours. (**B**) Illustration showing cardiolipin synthesis from phosphatidylglycerol (PG) and cytidine diphosphate diacylglycerol (CDP-DAG). Ratio of peak area corresponding to the total measured species of cardiolipin (CL) and its precursor PG from untargeted lipidomics performed on WT, NT-Control, and CRLS1 KD iBMDM mitochondrial isolates. (**C**) SDS-PAGE and IB analysis of ATP5A, UQCRC2, SDHB, and NDUFB8 from NT-Control and CRLS1 KD iBMDM. (**D**) Agilent Seahorse XF analysis of the oxygen consumption rate (OCR) and the extracellular acidification rate (ECAR) in 6-hour LPS-pretreated NT-Control and CRLS1 KD iBMDM during the Mito Stress Test assay, including additions 2 μM carbonyl cyanide *p*-trifluoromethoxyphenylhydrazone, 1.5 μM oligomycin, 0.5 μM rotenone, and 0.5 μM antimycin A at the indicated time points. (**E**) Quantification of basal OCR as in (D). (**F**) Quantification of basal ECAR paired to (D). (**G**) Representative confocal fluorescence micrographs from NT-Control and CRLS1 KD iBMDM stimulated with or without LPS (200 ng/ml) for 3 or 6 hours and then stained with the mitochondrial superoxide indicator MitoSOX and counterstain Hoechst. (**H**) Quantification of MitoSOX intensity per cell from ~300 cells per experiment using CellProfiler. Graphs presented as the mean of *n* ≥ 3 independent experiments with SD error bars. *P* values were calculated using a one-way ANOVA with Tukey’s posttest or two-way ANOVA with Sidak’s posttest. *P < 0.05; **P < 0.01; ****P < 0.0001.

As LPS stimulation triggers selective mitophagy of SDHB, we sought to determine the mechanism by which SDHB is selectively packaged and degraded while other mitochondrial proteins, like SDHA, are spared. Prior work has determined that the mitochondrial phospholipid CL regulates mitophagy upon exposure to the mitochondrial surface, a phenomenon that occurs during LPS stimulation (*27*, *28*). Furthermore, CL facilitates supramolecular organization and function of the respiratory complexes (*29*) and can stabilize Complex II in a biochemical nanodisc setting (*30*). Thus, we hypothesized that CL proximity to and/or interaction with Complex II might control selective mitophagy of SDHB during LPS stimulation. To investigate the role of CL in macrophage metabolism, we knocked down the terminal enzyme in the CL biosynthetic pathway, CL synthase (CRLS1 KD), and validated an effect on CRLS1 and CL levels by IB and untargeted lipidomics of mitochondrial fractions, respectively (Fig. 4, A and B, and fig. S12, A to D). Furthermore, we validated depletion of CL in CRLS1 KD macrophages with an orthogonal approach of mitochondrial inner membrane annexin V staining, which has been used previously to estimate membrane-associated CL (fig. S12, E to J) (*31*). We noted that CRLS1 KD did not significantly affect the basal abundance of the respiratory complex subunits tested, respiration, glycolysis, or membrane potential (Fig. 4, C to F, and fig. S13). These findings suggest that the minimum amount of CL needed for basal metabolism is achieved in CRLS1 KD macrophages. While CRLS1 KD macrophages maintained apparently normal basal metabolism, disruption of CL biosynthesis compromised the ability of macrophages to dampen respiration and switch to aerobic glycolysis during LPS stimulation (Fig. 4, D to F). Consistent with a failure to inhibit respiration, CRLS1 KD macrophages did not increase mitochondrial reactive oxygen species (ROS) during LPS stimulation, evidenced by decreased intensity of the mitochondrial superoxide indicator, MitoSOX, quantified by confocal microscopy (Fig. 4, G and H). Collectively, these data suggest that the ability of macrophages to remodel metabolism in response to LPS stimulation depends on CL biosynthesis.

### CL biosynthesis is required for Complex II destabilization and sequestration during macrophage inflammatory activation

**Fig. 5.**
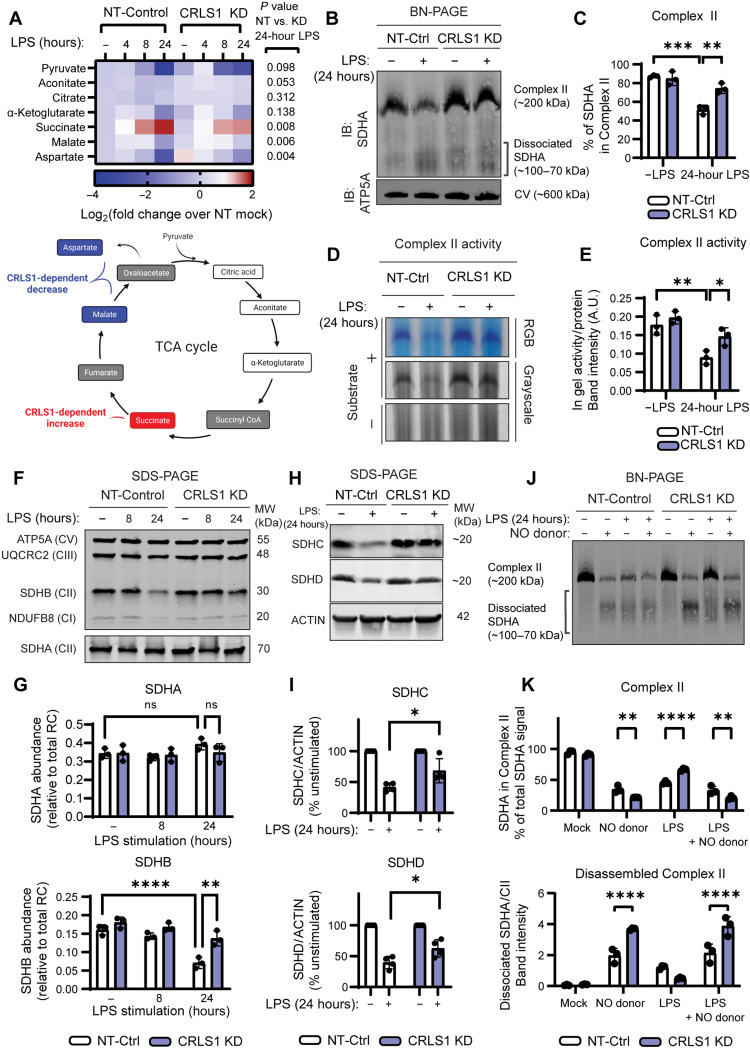
Cardiolipin biosynthesis is required for Complex II disassembly and degradation during LPS stimulation. (**A**) Highlighted TCA cycle metabolites from targeted metabolomics analysis of NT-Control and CRLS1 KD iBMDM stimulated with or withoutLPS (200 ng/ml) for 4, 8, or 24 hours. (**B**) BN-PAGE and IB analysis of SDHA and ATP5A from NT-Control and CRLS1 KD iBMDM stimulated with or withour LPS for 24 hours. (**C**) Percentage of SDHA in Complex II from (B). (**D**) CN-PAGE and in-gel SDH activity assay of NT-Control and CRLS1 KD iBMDM stimulated with or without LPS for 24 hours. (**E**) SDH in-gel activity normalized to sample protein. (**F**) SDS-PAGE and IB analysis of ATP5A, UQCRC2, SDHA, SDHB, and NDUFB8 from NT-Control and CRLS1 KD iBMDM stimulated with or without LPS for 8 or 24 hours. (**G**) Quantification of SDHB and SDHA abundance relative to total respiratory complex (RC) subunit signal. (**H**) SDS-PAGE and IB analysis of SDHC and SDHD in NT-Control and CRLS1 KD iBMDM stimulated with or withour LPS (200 ng/ml) for 24 hours. (**I**) Quantification of SDHC and SDHD abundance normalized to ACTIN as a percentage of the unstimulated condition. (**J**) BN-PAGE and IB analysis of SDHA from NT-Control and CRLS1 KD iBMDM costimulated with or without LPS and NO donor DETA NONOate (500 μM) for 24 hours. (**K**) Percentage of SDHA in Complex II and the ratio of dissociated SDHA to Complex II–associated SDHA from (J). Graphs presented as the mean of *n* = 3 independent experiments with SD error bars. *P* values were calculated using a two-way ANOVA with Sidak’s posttest. *P < 0.05; **P < 0.01; ***P < 0.001; ****P < 0.0001. (A) was adapted from BioRender.com assets.

Macrophage stimulation with LPS triggers rewiring of the TCA cycle, a process that is linked to inflammatory macrophage polarization (*32*). Since we identified a key role of CL biosynthesis in global metabolic remodeling in LPS-stimulated macrophages, we tested whether CRLS1 KD macrophages were defective in their capacity to alter TCA cycle metabolites in response to LPS. To this end, we performed a targeted metabolomics kinetic study to identify changes in metabolite levels between CRLS1 KD and NT-control macrophages during LPS stimulation. Our analysis revealed that LPS triggers a CRLS1-dependent break in the TCA cycle, whereby succinate, the substrate for Complex II, accumulates and downstream metabolites malate and aspartate are depleted (Fig. 5A and fig. S14A). We also observed decreased accumulation of itaconate in LPS-stimulated CRLS1 KD macrophages, a phenotype normally associated with Complex II dysfunction (fig. S14B) (*15*). Furthermore, we observed that CRLS1-dependent metabolic remodeling supported adaptation to energetic stress during LPS stimulation since CRLS1 KD macrophages, which had a normal ATP:ADP (adenosine diphosphate) ratio in the baseline, depleted the ATP pool more rapidly than NT-Control macrophages during LPS stimulation (fig. S14C). As succinate is a Complex II substrate and would be expected to accumulate when LPS triggers Complex II dysfunction, we hypothesized that CRLS1 KD macrophages might have a defect in Complex II regulation. We first tested whether CL contributes to LPS-induced destabilization of Complex II. To this end, we stimulated macrophages with or without LPS for 24 hours and measured the abundance and activity of Complex II by BN-PAGE and parallel IB and in-gel activity assays (Fig. 5, B to E). LPS-treated CRLS1 KD macrophages maintained Complex II levels and activity comparable to untreated cells, in contrast to LPS-treated NT-control macrophages. Furthermore, SDS-PAGE and IB analysis of RC subunits revealed that SDHB, SDHC, and SDHD, but not SDHA, were lost in NT-Control macrophages, but retained in CRLS1 KD macrophages (Fig. 5, F and G). Following CL biosynthesis by CRLS1, CL maturation depends on acyl chain remodeling by the phospholipid acyltransferase Tafazzin (TAZ) (*16*). We generated macrophages from inducible TAZ KD mice and found that TAZ was dispensable for LPS-induced SDHB degradation (fig. S15) (*33*, *34*). Accordingly, CL biosynthesis, but not acyl chain remodeling, is required for Complex II regulation during LPS stimulation.

**Fig. 6.**
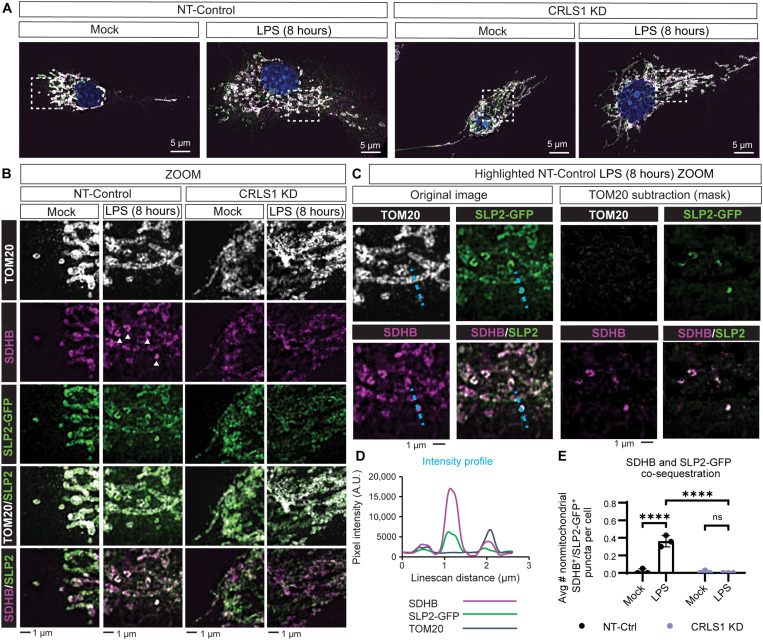
LPS triggers cardiolipin-dependent sequestration of SDHB. (**A**) Representative super-resolution 2D structured illumination microscopy (SIM) fluorescence micrographs from NT-Control and CRLS1 KD iBMDM expressing the CL-binding protein SLP2-GFP stimulated with or without LPS (200 ng/ml) for 8 hours and subjected to immunofluorescence labeling of SDHB and TOM20. (**B**) Magnified regions of interest from (A) highlighting LPS-induced SDHB and SLP2-GFP sequestration away from the TOM20-positive mitochondrial network. White arrowheads on the LPS-stimulated NT-Control SDHB image indicate SDHB/SLP2-GFP double-positive puncta. (**C**) Masking of the mitochondrial network based on TOM20 thresholding in LPS-stimulated NT-Control condition to highlight nonmitochondrial SDHB and SLP2-GFP signal. (**D**) Line scan analysis of the relative intensity of SDHB, SLP2-GFP, and TOM20 along the dotted cyan line in (C). (**E**) Automated quantification of the average number of co-sequestered SDHB and SLP2-GFP puncta per cell as illustrated in fig. S17. Graphs presented as the mean of *n* = 3 independent experiments with SD error bars. For SIM image analysis, the mean of ~30 cells per condition is reported for each independent experiment. *P* values were calculated using a two-way ANOVA with Sidak’s posttest. ****P < 0.0001.

While we predicted that CL biosynthesis would be important for sequestration and degradation of SDHB during LPS stimulation, we were surprised to observe that CL biosynthesis was critical for LPS-induced disassembly and inhibition of Complex II as well. We and others have observed that iNOS is required for Complex II disassembly during inflammatory macrophage activation (*14*). For this reason, we sought to determine whether CRLS1-dependent Complex II disassembly could be bypassed through NO supplementation with the NO adduct diethylenetriamine (DETA)-NONOate. NO supplementation was sufficient to trigger Complex II disassembly in CRLS1 KD macrophages, indicating that the missing signal for Complex II disassembly is NO. However, we also observed an accumulation of dissociated Complex II components in the DETA-NONOate-treated CRLS1 KD macrophages compared to NT-Control (Fig. 5, J and K). This unexpected finding could indicate that either CL protects the respiratory chain from the action of NO or the disposal of dysfunctional Complex II is defective in the CRLS1 KD macrophages, as indicated by the accumulation of dissociated Complex II. On the basis of these observations, we propose that CL plays dual roles in early signaling, which leads to NO production and later mitochondrial quality control. We observed that CRLS1 KD immortalized bone marrow–derived macrophage (iBMDM) did not produce iNOS and had markedly decreased l-citrulline production during LPS stimulation (fig. S16). Since CRLS1 KD macrophages less effectively degraded SDHB, we anticipated that SDHB sequestration would also be limited. Furthermore, we hypothesized that SDHB would co-sequester with other known CL-binding proteins in a CRLS1-dependent manner. To test these hypotheses, we inducibly expressed the CL-binding protein stomatin-like protein 2 tagged with GFP (SLP2-GFP) in CRLS1 KD and NT-Control macrophages and tracked localization of SLP2-GFP, Translocase of Outer Membrane-20 (TOM20), and SDHB by immunofluorescence and super-resolution 2D structured illumination microscopy (SIM) (Fig. 6A) (*35*, *36*). During LPS stimulation, subsets of both SDHB and SLP2-GFP were sequestered away from the mitochondrial network, as indicated by TOM20, in a CRLS1-dependent manner (Fig. 6B and fig. S17). SLP2-GFP and SDHB often co-sequestered and remained colocalized while losing colocalization with TOM20 (Fig. 6, C to E, and fig. S17). Modest differences in the Pearson’s correlation coefficient reflect our observation that small quantities of SDHB and SLP2-GFP appear to be shuttled away from the mitochondrial network gradually over time. Together, our data support that LPS-induced SDHB sequestration and degradation through mitophagy depend on CL.

### CL biosynthesis and modulation of Complex II activity are critical for early inflammatory responses in macrophages

**Fig. 7.**
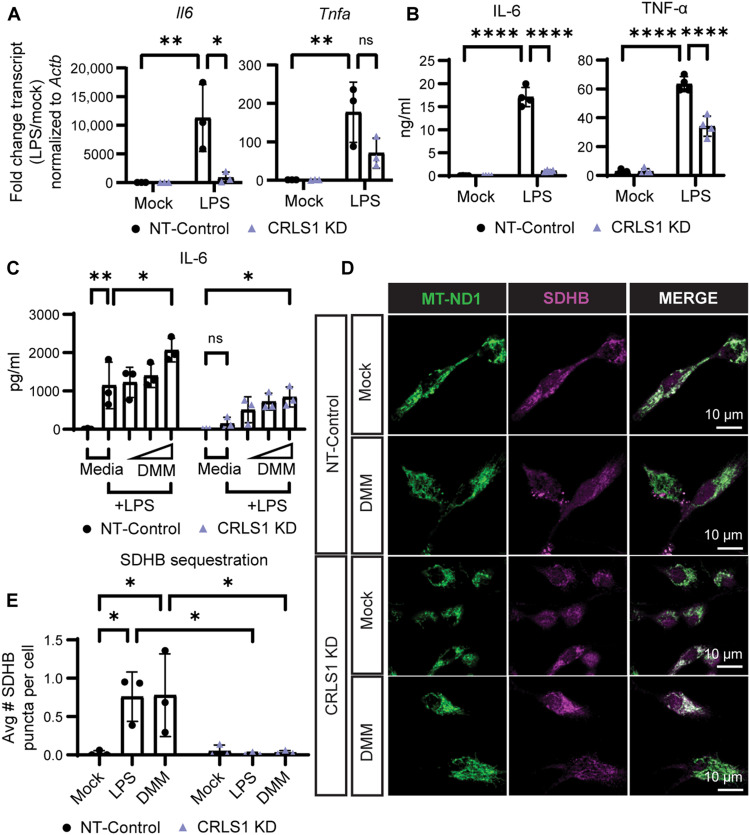
Cardiolipin biosynthesis and early Complex II inhibition contribute to inflammatory gene expression. (**A**) RT-qPCR analysis of transcript levels of *Il6* and *Tnf* in NT-Control and CRLS1 KD iBMDM stimulated with or without LPS (200 ng/ml) for 4 hours. (**B**) Enzyme-linked immunosorbent assay (ELISA) analysis of IL-6 and TNF-α secreted by NT-Control and CRLS1 KD iBMDM stimulated with LPS (200 ng/ml) for 24 hours. (**C**) ELISA analysis of IL-6 secreted by NT-Control and CRLS1 KD iBMDM pretreated for 1 hour with or without 0.1, 1, or 10 mM dimethyl malonate (DMM) and then challenged with LPS (200 ng/ml) for 6 hours. (**D**) Representative confocal fluorescence micrographs from NT-Control and CRLS1 KD iBMDM stimulated with or without 10 mM DMM for 8 hours and subjected to immunofluorescence labeling of SDHB and MT-ND1. (**E**) Quantification of SDHB puncta in NT-Control and CRLS1 KD iBMDM stimulated with 10 mM DMM or LPS (200 ng/ml) for 8 hours, quantified as described in Fig. 2D. The complete set of representative confocal fluorescence micrographs is shown in fig. S21. Graphs presented as the mean of *n* = 3 independent experiments with SD error bars. For image analysis, the mean of ~100 cells per condition is reported for each independent experiment. *P* values were calculated using a two-way ANOVA with Sidak’s posttest. *P < 0.05; **P < 0.01; ****P < 0.0001.

Complex II has been implicated in macrophage inflammatory function through HIF-1–driven inflammatory gene expression (*10*, *28*). With this observation in mind, we hypothesized that interaction between CL and Complex II may regulate macrophage inflammatory programming. To test this hypothesis, we stimulated NT-Control and CRLS1 KD macrophages with LPS and measured transcript levels of hallmark inflammatory genes interleukin-6 (*Il6*) and tumor necrosis factor (*Tnf*) by RT-qPCR. CRLS1 KD macrophages exhibited markedly decreased levels of *Il6* transcript compared to NT-control, with only a slight nonsignificant defect in transcript levels of *Tnfa* (Fig. 7A). In parallel, we measured secreted levels of these cytokines by enzyme-linked immunosorbent assay (ELISA) (Fig. 7B). Similar to transcript levels, we found that CRLS1 was required for LPS-induced production of IL-6, with a moderate contribution to TNF-α secretion. We performed a broader panel of ELISAs for LPS-stimulated cytokines and chemokines from CRLS1 KD and NT-Control macrophage supernatants, which revealed three classes of CRLS1 dependency in cytokine production: First, CRLS1 was required for the production of cytokines IL-6, pro–IL-1β, IL-1α, and transforming growth factor-beta (TGF-β); second, CRLS1 partially contributed to the production of TNF-α, C-C motif chemokine ligand (CCL)5, C-X-C motif chemokine ligand (CXCL)2, and CXCL10; and, last, CRLS1 was modestly or not at all important for the production of CCL2, CCL3, and CCL4 (fig. S18, A to C). To determine whether CRLS1 was important for inflammatory responses in other types of macrophages beyond bone marrow–derived macrophages (BMDMs), we knocked down CRLS1 in the peritoneal origin RAW264.7 murine macrophage cell line and saw similar CRLS1-dependent inflammatory responses (fig. S18, D to F). Last, we tested the effect of CRLS1 KD on broader macrophage effector functions including phagocytosis, bacterial killing, and cytokine production during bacterial infection (fig. S19). We observed that CRLS1 KD macrophages retained the ability to phagocytose the bacterial pathogen STM and nonpathogenic *Escherichia coli* (EC). CRLS1 KD macrophages could effectively kill EC yet were defective in IL-6 production in response to both STM and EC. These findings indicate that CL biosynthesis regulates expression of a subset of inflammatory gene in macrophages.

**Fig. 8.**
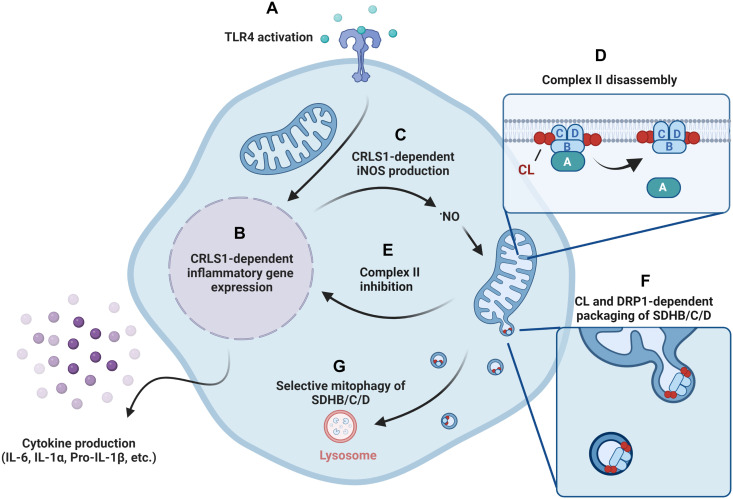
Regulation of macrophage inflammatory and metabolic reprogramming by cardiolipin. Activation of TLR4 by LPS triggers robust inflammatory and metabolic reprogramming in macrophages (**A**). The biosynthesis of the mitochondrial phospholipid CL by CL synthase (CRLS1) is critical for inflammatory programming, especially the production of IL-6, IL-1α, pro–IL-1β, and iNOS (**B** and **C**). NO triggers Complex II disassembly through SDHA dissociation (**D**). This route of Complex II inhibition contributes to acute inflammatory programming in macrophages (**E**). Disassembled Complex II, comprising SDHB, SDHC, and SDHD, is sequestered in a CL-dependent manner and released from the mitochondrial network through DRP1-dependent mitochondrial fission (**F**). A CL-dependent form of mitophagy promotes the selective degradation of SDHB, SDHC, and SDHD (**G**). This figure was made with BioRender.com.

Our results provide evidence that CL biosynthesis is critical for inflammatory programming in macrophages, and work from other laboratories collectively supports that Complex II regulates inflammatory responses in macrophages. To test whether these are related processes, we treated LPS-stimulated NT-control or CRLS KD macrophages with two different commercially available Complex II inhibitors, atpenin A5 (AA5) or dimethyl malonate (DMM), at a range of subcytotoxic concentrations and measured IL-6 production by ELISA (Fig. 7C and fig. S20A). We found that both Complex II inhibitors partially restored IL-6 production in CRLS1 KD macrophages within the first 6 hours of treatment. We conclude that CL biosynthesis is critically important for inflammatory programming in macrophages and that there are likely Complex II–dependent and –independent components to this programming. Exogenous addition of diethyl succinate did not rescue IL-6 production, indicating that modulation of Complex II activity may be more nuanced than accumulation of succinate for inflammatory outcomes in the early phase of inflammatory activation (fig. S20B). Last, we tested whether inhibition of Complex II was sufficient to trigger SDHB sequestration. We found that DMM-treated macrophages sequestered SDHB into puncta to a similar extent as LPS-treated cells (Fig. 7, D and E, and fig. S21). In addition, DMM-induced SDHB sequestration was dependent on CRLS1. Thus, we speculate that CL biosynthesis contributes to separable processes of Complex II disassembly and sequestration of dysfunctional Complex II subunits targeted for degradation (Fig. 8).

## DISCUSSION

TLR4 activation by LPS triggers global metabolic remodeling, shifting away from respiration and toward glycolysis in macrophages, repurposing the respiratory chain for host defense and inflammatory signaling (*9*, *10*, *14*, *15*). Inhibition of the respiratory complexes themselves triggers substantive metabolic reprogramming and is sufficient to stimulate a robust glycolytic burst (*37*). From these observations, we predicted that one or more of the respiratory complexes would be negatively regulated by LPS stimulation. We found that Complex II activity decreased, and its components were disassembled in response to LPS stimulation. Following disassembly, Complex II subunit SDHB was sequestered into microdomains of the mitochondrial network and released in a DRP1-dependent manner. SDHB released from the mitochondrial network through this mechanism was turned over through endolysosomal degradation in a selective form of mitophagy. Biosynthesis of the mitochondrial phospholipid CL was critical for disassembly of Complex II, and sequestration and degradation of SDHB, SDHC, and SDHD. Last, we show that defective CL biosynthesis compromises the induction or stability of pro-inflammatory cytokine transcripts, notably IL-6, a phenotype that could be partially rescued by Complex II inhibition. Collectively, our work highlights the importance of CL in regulating macrophage inflammatory programming through coordination of Complex II disassembly and degradation (Fig. 8).

Ample evidence supports an integral role for regulation of ETC components in executing the shift from homeostatic to inflammatory programming (*38*). Environmental cues alter macrophage metabolism, which guides polarization between inflammatory and anti-inflammatory macrophage phenotypes. Metabolic changes influence cellular fate through a variety of factors, including oxidative stress pathways, posttranslational modification of proteins by induced metabolites, and epigenetics (*10*, *15*, *39*–*41*). Mills *et al.* (*9*) demonstrated that succinate produced through the GABA shunt enhanced LPS-induced IL-1β production, while conditional deletion of the *Sdhb* gene in macrophages or pharmacological inhibition of Complex II, both approaches that increase succinate pools, paradoxically compromised IL-1β production during prolonged LPS stimulation. The model that best fits these data is that succinate oxidation by Complex II participates in inflammatory signaling. Nevertheless, our data demonstrate that pharmacological inhibition of Complex II in the acute early phase of LPS stimulation enhances inflammatory responses, particularly IL-6 production. Furthermore, our data demonstrate that succinate oxidation alone cannot account for the inflammatory contribution of Complex II, as pro-inflammatory cytokine production in CRLS1 KD macrophages, which maintain SDH activity, cannot be rescued by exogenous succinate supplementation. Together, our work and prior studies point to dynamic regulation of both Complex II and succinate levels as critical for orchestrating both the acute inflammatory response as well as the subsequent shift from pro- to anti-inflammatory macrophage function. Prior work has identified that alternative assembly of Complex II, reminiscent of what we have observed, aids in the adaptation to energetic stress (*42*). Following the model of alternative assembly, one possibility is that the SDHA/SDHB dimer dissociates from the integral membrane SDHC/SDHD dimer to alter Complex II function. At 8 hours after LPS stimulation, we observed by BN-PAGE and SDHA IB a banding pattern consistent with multiple SDHA-containing species dissociated from Complex II (fig. S4B). However, since LPS triggers a CRLS1-dependent loss of SDHC and SDHD (Fig. 5, H and I), the most parsimonious explanation is that SDHB remains associated with the integral membrane proteins SDHC and SDHD to facilitate sequestration, while SDHA dissociates and remains soluble in the mitochondrial matrix.

Recent findings indicate that inhibitors of Complex I, II, III, or V prevent NOD-, LRR-, and pyrin domain–containing protein 3 (NLRP3) inflammasome activation and IL-1β production, by sustaining high ATP levels in a ROS-independent manner (*43*). Although observations using pharmacological inhibition must be interpreted with caution, the preponderance of data supports the idea that components of the respiratory complexes are prime targets for multilevel regulation. In addition to alterations in transcript or protein levels, Clayton *et al*. (*44*) determined that, in response to inflammatory signals, the Complex IV subunit NADH dehydrogenase ubiquinone alpha subunit 4 (NDUFA4) is replaced by a paralogous component, C15ORF48. The expression of *C15orf48* transcript is positively correlated with disease severity in patients with rheumatoid arthritis. In addition, macrophages from human patients genetically lacking NDUFA4 exhibit hyperinflammatory characteristics, independent from changes in cellular ATP production rates, again underscoring the complex relationship between the function of respiration and its component parts (*44*). Our findings identify a regulatory modality that controls availability and function of SDHB protein during the early pro-inflammatory macrophage response, thereby tuning the activity of Complex II. Our results further establish that CL, a key architect of homeostatic mitochondrial function, plays a prominent and distinct role in Complex II regulation, and possibly other respiratory complexes, during LPS-induced mitochondrial reprogramming that leads to inflammation.

Metabolic reprogramming is implicated in the pathophysiology of many human diseases, with changes in respiratory chain function evident in many disease states. Gene-encoding components of Complex II are mutated in several types of cancer, including hereditary paraganglioma due at least in part to increased ROS production and cell proliferation (*45*–*47*). In β cells, deficiency in Complex II leads to metabolic dysfunction and diabetes in a mouse model of disease (*48*). A substantial body of evidence therefore points to Complex II as a critical regulatory nexus for metabolic reprogramming and accordingly positions it as a target of multiple layers of regulation. Most work to date has focused on transcriptional regulation and posttranslational modifications as regulatory determinants of Complex II abundance and activity. Posttranslational regulatory mechanisms, particularly degradative pathways, have been identified for other respiratory complexes, including multiple subunits of Complex I, but not Complex II (*49*). Last, the activity, abundance, and assembly of respiratory complexes are affected under diverse biological contexts. Thus, our observations that Complex II stability and activity are disrupted by TLR4 activation in macrophages fits into the broader context of complex regulation of the RCs. We propose that control of Complex II disassembly enables rapid modulation of inflammatory metabolism in macrophages. Notably, we observe a trending drop in Complex II activity as early as 4 hours after LPS stimulation and a substantial drop in respiration by 6 hours after LPS treatment. Furthermore, these early metabolic changes may regulate gene expression during inflammatory signaling. LPS-induced TCA cycle remodeling alters inflammatory signaling through posttranslational modifications of signaling molecules and epigenetic decoration of histones by TCA intermediates (*50*). The multiprotein respiratory complexes present many molecular interfaces for posttranslational regulation and how such mechanisms determine the nature and magnitude of inflammation is ripe for further study.

Different inflammatory stimuli elicit unique metabolic and inflammatory programs in macrophages (*3*). These metabolic changes are regulated by complex and multifactorial mechanisms (*51*). One particular mechanism for the regulation of mitochondrial enzymes is control of protein abundance. The abundance of mitochondrial proteins can be regulated by a variety of mechanisms including gene expression, efficiency of mitochondrial import, degradation by local proteases, and organelle-level autophagic turnover of mitochondria (mitophagy). Here, we provide evidence that degradation of the Complex II subunit SDHB occurs through a selective mitophagy mechanism, whereby specific cargoes are enriched into fragmented mitochondria and targeted for endolysosomal degradation. Prior work from our laboratory and others has identified that TLR4 activation triggers the generation of a pool of small, fragmented mitochondria through the activity of DRP1 (*23*, *52*). While the purpose of these fragmented mitochondria was previously unclear, data from this study support that a subset of these fragmented mitochondria are enriched with specific mitochondrial cargo, including SDHB, to be turned over through mitophagy. CL organizes the mitochondrial inner membrane into functional microdomains, where it can facilitate respiratory supercomplex assembly and function (*16*). Our CRLS1 KD macrophages did not have a respiratory defect. One CL species was preferentially preserved even in the context of CRLS1 KD (fig. S12). This particular acyl chain state is maintained by a CL-specific acyltransferase, TAZ (*53*). Thus, we speculate that preservation of this CL species, or a combination of the remaining species, is sufficient to sustain homeostatic respiratory chain function. Alternatively, normal respiration in the CRLS1 KD macrophages may be attributed to a compensatory increase in phosphatidylethanolamine (PE). PE has been shown to support RC function and a compensatory increase in this phospholipid may sustain RC function when CL is limiting (*17*).

CRLS1 deficiency did not disrupt homeostatic respiration in macrophages, but these cells failed to remodel their metabolism in response to LPS stimulation, pointing to a key role for CL specifically in metabolic plasticity. We observed that CL biosynthesis is required for the glycolytic burst and the production of mitochondrial superoxide, both of which are linked to inflammatory signaling in macrophages (*9*, *10*). Furthermore, we found that CL biosynthesis was required for selective inflammatory gene expression, where LPS-induced *Il6* transcript levels were more sensitive to CL biosynthesis than *Tnf* transcript levels. This effect was partially dependent on CL-dependent modulation of Complex II stability but was not completely restored by pharmacological Complex II inhibition. Our results indicate that CL biosynthesis contributes to inflammatory signaling through multiple routes. CL, which is exposed to the outer mitochondrial membrane through membrane contact sites generated by the protein Non-metastatic cells 4 (NME4), contributes to inflammatory signaling upstream of nuclear factor κB (*31*). Thus, CL may contribute to metabolic changes and inflammatory signaling cascades to strictly control inflammatory responses at the transcript level. In addition, CL is proposed to scaffold the assembly of the NLRP3 inflammasome (*28*). Thus, CL contributes to inflammatory programming in macrophages at multiple levels. Within this context, we have identified a fundamental role for CL in early metabolic changes in macrophages that likely shapes later processes of gene expression and posttranslational regulation of inflammatory responses. Together, our work reveals multiple mechanisms by which macrophages adapt their metabolism to inflammatory cues. We have identified a CL-dependent route by which Complex II is disassembled and degraded in macrophages. Furthermore, our work has revealed that CL enables selective mitophagy of SDHB during inflammatory macrophage activation, without depleting its partner, SDHA. We speculate that CL microdomains may more generally provide the spatial architecture for selective packaging of mitochondrial cargo, acting as a key platform to execute different metabolic programs.

## MATERIALS AND METHODS

### Ethics statement

All animals used for experimental protocols were housed in specific pathogen–free facilities at the University of Michigan Medical School Unit for Laboratory Animal Medicine (ULAM) and treated humanely in accordance with an Institutional Animal Care Use for Research Committee–approved protocol (PRO00010463). Blood samples were obtained from healthy adult donors according to the protocol approved by the University of Michigan Medical School (HUM00044257). Written consent was obtained from all donors.

### Cell culture

Murine iBMDMs were generated as previously described (*54*–*56*). Briefly, recombinant Cre-J2 virus containing v-Raf and v-Myc oncogenes was generated in 3T3 fibroblasts grown in Dulbecco’s modified Eagle’s medium (DMEM) supplemented with 10% heat-inactivated fetal bovine serum (FBS) and penicillin (50 U/ml) and streptomycin (50 μg/ml). Sterile-filtered culture supernatants containing Cre-J2 virus were stored at −80°C. C57BL/6J lineage mouse femurs and tibiae were flushed and cells were transduced with Cre-J2 virus in macrophage differentiation media [50% DMEM, 2 mM l-glutamine, 1 mM sodium pyruvate, 30% L929 cell-conditioned medium, 20% FBS, penicillin (50 U/ml), and streptomycin (50 μg/ml)]. iBMDMs were grown for at least 1 month before use in experiments to ensure that immortalization was successful. L-929 cells were cultured in minimum essential Eagle’s medium supplemented with 2 mM l-glutamine, 1 mM sodium pyruvate, 1 mM nonessential amino acid, 10 mM Hepes, and 10% FBS. All experiments were performed in DMEM supplemented with 2 mM l-glutamine, 1 mM sodium pyruvate, and 10% FBS, unless otherwise indicated. *Nos2*^−/−^ (strain #002609) and *Acod1*^−/−^ (Strain #029340) mice and wild-type (WT) littermate pairs were obtained from the Jackson Laboratory. BMDMs were derived according to the protocol described above. Effective knockout was validated by IB following 24-hour stimulation with LPS (200 ng/ml) using iNOS antibody (1:1000; Cell Signaling Technology, 2982S) and IRG1 antibody (1:1000; Abcam, ab222411). Doxycycline-inducible TAZ KD (Strain #014648) and littermate WT pair mice were obtained from the Jackson Laboratory and used to generate iBMDM as described above. Effective KD was validated using IB analysis of TAZ with an anti-TAZ antibody (1:1000) provided by the laboratory of S. Claypool (*34*). When indicated, macrophages were treated with LPS (200 ng/ml) derived from STM (Sigma-Aldrich, L2262). In addition, a variety of inhibitors were used in this study, the concentration and source of which are included in the appended table (table S1). Throughout culturing, all cells were incubated at 37°C in 5% CO_2_.

### Human macrophage differentiation

Blood samples were obtained from healthy adult donors. Peripheral blood mononuclear cells were isolated by Ficoll separation and differentiated in vitro into hMDMs. Briefly, blood was layered on Ficoll and centrifuged at 500*g* at room temperature (RT) with low acceleration and no breaks for 20 min. Plasma was discarded, and the buffy coat and the layer beneath were collected, centrifuged, and resuspended in RPMI 1640 containing 20% FBS and human macrophage colony-stimulating factor (50 ng/ml) (PeproTech, 300-25). Cells were seeded into sterile tissue-culture plates and differentiated for 7 days. At 3 days after isolation, cultures were supplemented with fresh media. On the day of the experiment, cells were washed three times with phosphate-buffered saline (PBS) without Ca^2+^ or Mg^2+^ to remove nonadherent cells. Differentiation of macrophages by this protocol was validated by detection of CD68 (1:1000; BioLegend, 333821) and CD11b (1:1000; BioLegend, 301329) double positivity by flow cytometry.

### Murine endotoxemia model and intracellular SDHB flow cytometry in splenocytes

Male C57BL/6J (Strain #00064) mice (14 to 16 weeks) were purchased from the Jackson Laboratory and were housed in specific pathogen–free and climate-controlled facilities at the University of Michigan Medical School ULAM. Chow (5L0D) and water were provided ad libitum. On the day of the experiment, mice were injected intraperitoneally with LPS (20 mg/kg) derived from STM (Sigma-Aldrich, L2262) in PBS (five animals) or PBS alone (four animals) and monitored for the duration of the experiment. Animals were euthanized at the 24-hour time point and the spleen was collected and mechanically separated into a single-cell suspension by passage through a sterile 40-μm cell strainer (Thermo Fisher Scientific, 22-363-547). Red blood cells (RBCs) were lysed with 5 min of 1× RBC lysis buffer (eBioscience, 00-4300-54) at RT. Cells were washed with PBS and then fixed with 4% paraformaldehyde (PFA) for 15 min. Cells were permeabilized with 0.1% Triton X-100 in PBS (wash buffer) for 20 min. Cells were blocked with 5% bovine serum albumin (BSA) (Thermo Fisher Scientific, BP1600-100) in wash buffer (block buffer) at RT for 30 min. Cells were incubated with anti-SDHB antibody (Proteintech, 10620-1-AP) or Rabbit immunoglobulin G (IgG) isotype control (Thermo Fisher Scientific, PI31235) in block buffer (0.3 μg/10^6^ cells) for 45 min. Cells were incubated with different cocktails of fluorescent secondary antibody anti-Rabbit IgG AF488 (1:100; Thermo Fisher Scientific, A11034) or anti-Rabbit IgG AF647 (1:100; Thermo Fisher Scientific, A21244) and lineage-marker primary-conjugated fluorescent antibodies anti-CD3 Pacific Blue (1:100; BioLegend, 100213), anti-CD19 FITC (1:100; SouthernBiotech, 1575-02), anti-CD11b BV421 (1:100; BioLegend, 101235), and/or anti-CD11b allophycocyanin (APC) (1:100; SouthernBiotech, 1561-11). Cells were washed and immediately analyzed by flow cytometry (Fortessa, BD Biosciences). Gating on Forward Scatter (FSC)/Side Scatter (SSC) was used to exclude cell debris or clumps. The isotype control was used to validate specific staining, and fluorescence minus one controls were used to guide in subset gating. Within each cell population, the geometric mean of SDHB staining was recorded.

### Bacterial infection

STM (SL1344) and cloning grade EC (TOP10) were grown overnight in LB in a 37°C slanted, shaking incubator (250 rpm). Bacteria were washed three times and diluted in sterile PBS. iBMDMs were infected with STM or EC at an MOI of 25 (or as otherwise indicated) for 1 hour, and extracellular bacteria were killed with high-concentration gentamicin treatment (100 μg/ml) for 1 hour. Cells were washed three times with PBS and then either lysates were harvested in sterile water for the 1 hour post-infection (hpi) time point or the supernatant was replaced with media containing gentamicin (10 μg/ml) for a final time point of 24 hpi. After 24 hpi, lysates were collected in sterile water and plated on LB agar plates for colony-forming units (CFU). Bacterial killing was calculated as the percentage of CFU at 1 hpi minus the CFU at 24 hpi divided by the CFU at 1 hpi. Supernatants from STM, EC, or mock-infected iBMDMs were collected for cytokine analysis at 1 and 24 hpi.

### Phagocytosis assay

STM (SL1344) and cloning grade EC (TOP10) were transformed to express dsRED via the plasmid pGEN222::Pem7-DsRED and selected for ampicillin resistance. Bacteria were grown overnight at 37°C in LB containing ampicillin (100 μg/ml), slanted with 250 rpm shaking. Bacteria were washed with sterile PBS three times. iBMDMs were infected with either MOI 25 STM-dsRED or EC-dsRED for 1 hour. Cells were washed three times with PBS and directly analyzed by flow cytometry (Fortessa, BD Biosciences). The percentage of dsRED-positive cells is reported.

### Generation of DRP1 KD and CRLS1 KD

Stable KD of DRP1 and CL synthase (CRLS1) in iBMDM was achieved using lentiviral delivery of shRNA. Lentivirus was generated and packaged in human embryonic kidney (HEK) 293T cells grown in DMEM supplemented with 10% FBS. HEK293T cells were transfected with pLKO.1 plasmid encoding *Drp1*-targeted shRNA, *Crls1*-targeted shRNA, or a NT-Control along with the packaging plasmids (pHCMV-G, and pHCMV-HIV-1) (*57*) using FUGENE-HD transfection reagent (Promega). The mouse *Drp1*-targeted shRNA plasmid with the sense sequence of GGCAATTGAGCTAGCTATA, *Crls1*-targeted shRNA plasmid with the sense sequence of GAAGACTTTAATGTTGCACTA, and the NT-Control shRNA plasmid were purchased from Sigma-Aldrich. Lentivirus-containing supernatants were collected, filtered, and used to transduce macrophage cell lines. Transduced cells were selected with puromycin (3 μg/ml), and resistant cells were grown and used for the experiments.

### Mitochondrial isolation

Mitochondria were isolated from cells by subcellular fractionation and density centrifugation as previously described (*58*). Briefly, iBMDMs were washed three times with ice-cold Dulbecco’s PBS (DPBS), centrifuged at 500*g*, 4°C, for 5 min, and then resuspended in ice-cold mitochondrial isolation buffer (MIB), composed of 0.25 M sucrose, 20 mM Hepes (pH 7.4), 2 mM EGTA, 10 mM KCl, 1.5 mM MgCl_2_, 0.1% fatty acid-free BSA (Sigma-Aldrich, A8806), and Halt Protease inhibitor. Cells were lysed on ice using a sterile 27G syringe and centrifuged at 1100*g*, 4°C, for 3 min to remove unlysed cells and cell debris. Mitochondria were pelleted from the clarified cell lysate by centrifugation at 12,000*g*, 4°C, for 15 min. The supernatant from this step was kept as the cytosolic fraction, and the mitochondria-containing pellet (mitochondrial fraction) was resuspended in MIB and centrifuged at 20,000*g*, 4°C, for 10 min. The supernatant was discarded and the mitochondrial fraction was again resuspended in MIB and centrifuged at 20,000*g*, 4°C, for 5 min. The mitochondrial fraction was washed twice with ice-cold DPBS at 20,000*g*, 4°C, for 5 min, immediately analyzed or flash-frozen in liquid nitrogen and stored at −80°C for subsequent analysis.

### Untargeted lipidomics

WT, CRLS1 KD, and NT-Control macrophages were grown overnight in DMEM supplemented with 10 mM glucose, 1 mM pyruvate, 2 mM glutamine, and 10% FBS. Mitochondria were isolated as described above, flash-frozen in liquid nitrogen, and stored at −80°C. Mitochondrial isolates were quality-controlled and tested for cytosolic contamination by IB analysis of TOM20 and GAPDH (glyceraldehyde-3-phosphate dehydrogenase), and total protein stains from SDS-PAGE–separated mitochondrial fractions (Revert 700, LI-COR) were used for sample loading normalization for lipidomics data. Untargeted lipidomics analysis was performed after general lipid extraction using a methyl tert-butyl ether (MTBE)–based liquid-liquid protocol. Samples were thawed at RT and 200 μl of PBS and 500 ml of methanol containing 20 ml of an internal standard mixture (custom mixture from Cayman Chemical; see associated documentation) were added to each sample. Samples were vortexed, and 1000 ml of methanol and 5 ml of MTBE were sequentially added to each sample. After additional vortexing, the mixture was incubated on a tabletop shaker at 500 rpm at RT for 1 hour. Phase separation was induced by the addition of 1.25 ml of water. Samples were sonicated for 10 min and then centrifuged at 2000*g* for 20 min. The upper organic phase of each sample was carefully removed using a Pasteur pipette and transferred into a clean glass tube. The remaining aqueous phase was re-extracted with 2.5 ml of the upper phase of MTBE/methanol/water 10:3:2.5 (v/v/v) solvent mixture, whose composition was similar to the expected composition of the upper phase. After vortexing and centrifugation as above, the organic phase was collected and combined with the initial organic phase. The extracted lipids were dried overnight in a SpeedVac concentrator. The dried lipid extracts were reconstituted in 200 μl of *n*-butanol/methanol 1:1 (v/v) and transferred into autosampler vials for analysis by liquid chromatography–tandem mass spectrometry (LC–MS/MS). The parameters of the LC–MS/MS settings can be found in the supplementary data files. Lipostar software (version 2.0.2b3; Molecular Discovery) was used for feature detection, noise and artifact reduction, alignment, normalization, and lipid identification. Significantly changed lipids between CRLS1 KD and NT-Control macrophages were identified using unpaired *t* tests and filtering of *P* < 0.05 and absolute value of log_2_(fold change) > 0.5. The full dataset is available in the Supplementary Materials (data S1).

### Mitochondrial annexin V staining

Mitochondrial fractions were prepared by density centrifugation of syringe-lysed CRLS1 KD and NT-Control iBMDM. The outer mitochondrial membrane was permeabilized with 0.005% digitonin in PBS for 20 min on ice. Permeabilization was performed concurrently with annexin V AF647 (1:1000; BioLegend 640911) or anti-TOM20 Coralite594 (1:100; Proteintech, CL594-11802) staining. Mitochondrial staining was analyzed by flow cytometry (Fortessa, BD Biosciences).

### Metabolomics

CRLS1 KD and NT-Control macrophages were grown overnight in DMEM supplemented with 10 mM glucose, 1 mM pyruvate, 2 mM glutamine, and 10% FBS and then stimulated with or without LPS (200 ng/ml) for 4, 8, or 24 hours. Stimulation was synchronized such that cells across conditions were in culture for the same amount of time. After stimulation, cells were washed twice with ice-cold DPBS, and metabolites were extracted by adding cold 80% methanol, incubating at −80°C for 10 min, followed by centrifugation at 17,000*g* for 10 min at 4°C. The resulting metabolite supernatant was collected. Metabolite extracts were normalized to protein content from paired samples, and the normalized fraction was dried using a SpeedVac at 4°C for 8 hours. Dried metabolite pellets were resuspended in a 50:50 mixture of MeOH and water. LC-MS/MS–based metabolomics were performed, and the data were analyzed as previously described (*59*, *60*). Briefly, samples were run on an Agilent 1290 Infinity II LC-6470 Triple Quadrupole (QqQ) tandem mass spectrometer system consisting of the 1290 Infinity II LC Flexible Pump (Quaternary Pump), the 1290 Infinity II Multisampler, the 1290 Infinity II Multicolumn Thermostat with a six-port valve, and the 6470 triple quad mass spectrometer. Agilent Masshunter Workstation Software LC/MS Data Acquisition for 6400 Series Triple Quadrupole MS with Version B.08.02 was used for compound optimization, calibration, and data acquisition. Significantly changed metabolites between CRLS1 KD and NT-Control macrophages were identified using *t* tests and filtering of *P* < 0.05 and absolute value of log_2_(fold change) > 0.5. The full dataset is available in the Supplementary Materials (data S2).

### Seahorse extracellular flux assay

An Agilent Seahorse XF96 analyzer was used to simultaneously measure the rate of oxygen consumption and extracellular acidification from cultured macrophages. Macrophages were plated in a 96-well Seahorse plate and allowed to adhere overnight in Seahorse XF DMEM supplemented with 10 mM glucose, 1 mM pyruvate, 2 mM glutamine, and 10% FBS. On the following day, medium was replaced with or without LPS (200 ng/ml) and cells were stimulated for 6 hours. After 6 hours, medium was exchanged for DMEM supplemented with the same levels of glucose, pyruvate, and glutamine without FBS. Cells were kept in a 37°C incubator without CO_2_ for 30 min before analysis. In some experiments, the Mito Stress Test kit from Agilent was used to probe different aspects of mitochondrial function with manufacturer-recommended concentrations of respiratory chain inhibitors, 2 μM carbonyl cyanide *p*-trifluoromethoxyphenylhydrazone (FCCP), 1.5 μM oligomycin, 0.5 μM rotenone, and 0.5 μM antimycin A. Comparable plating between conditions was confirmed by staining plates after analysis with CellTracker Red and Hoechst dye and analysis using a Biotek Synergy H1 plate reader.

### Protein extraction, SDS-PAGE, and IB analysis

At experimental endpoints, cells were washed twice with ice-cold DPBS and then lysed in 1% NP-40 lysis buffer supplemented with Halt Protease and phosphatase inhibitors for 15 min on ice. A Bio-Rad protein assay was used to normalize sample loading before SDS-PAGE. Samples were diluted in Laemmli sample loading buffer, heated for 5 min at 95°C, and then loaded onto precast 4 to 20% polyacrylamide tris-glycine gels (Bio-Rad). For CRLS1, SDHC, and SDHD IB, samples were instead heated for 30 min at 50°C to prevent precipitation. After SDS-PAGE, protein was transferred to a 0.45-μm nitrocellulose membrane by a semi-dry transfer system (Cytiva). Membranes were blocked with 5% BSA and 0.1% Tween 20 (IB blocking buffer) for 30 min at RT and then incubated with primary antibody in IB blocking buffer overnight at 4°C. Blots were developed using LI-COR IRdye secondary antibodies and an Odyssey IR Imager.Quantification of IBs was performed using ImageJ densitometric gel analysis protocol for 1D gels. A list of all antibody sources, concentrations, and applications is found in table S1.

### Immunoprecipitation of Complex II

Whole-cell extracts were prepared by solubilization in 1% dodecyl maltoside supplemented with Halt protease and phosphatase inhibitors on ice for 30 min. The Pierce Cross-link immunoprecipitation (IP) kit was used to conjugate 5 μg of SDHA antibody (14865-1-AP) to protein A/G agarose resin. Complex II was immunoprecipitated from whole-cell extracts overnight at 4°C with rocking. Complex II was eluted according to the Pierce Cross-link IP kit, and the pH was neutralized with pH 9.5 tris-HCl before SDS-PAGE analysis.

### Blue native–PAGE

BN-PAGE analysis of native respiratory complexes was performed as previously described (*61*) using commercially available reagents (Thermo Fisher Scientific). In summary, whole-cell extracts were prepared by solubilization of 10^6^ cells with 2 mg of digitonin and 1× native-PAGE sample buffer (Thermo Fisher Scientific, BN2008) on ice for 30 min. Subsequently, insoluble material was removed by 10 min of 17,000*g* centrifugation at 4°C. The soluble fraction was supplemented with 0.5% Coomassie G-250 immediately before loading samples onto Native PAGE 4 to 16% bis-tris mini gels from Invitrogen. Samples were electrophoresed at 4°C for 30 min in dark blue cathode at 150 V (Thermo Fisher Scientific, BN2007). After this initial electrophoresis, the cathode buffer was switched to the light blue cathode buffer. Following electrophoresis, gels were washed with ultrapure water and then soaked with 2× NuPAGE transfer buffer without methanol supplemented with 0.04% SDS for 15 min (Thermo Fisher Scientific, NP0006). Protein was transferred to an Immobilon-FL polyvinylidene difluoride membrane at 15 V for 15 min with 2× NuPAGE transfer buffer supplemented with 10% methanol using a Bio-Rad Transblot Turbo semi-dry transfer system. Immediately following transfer, proteins were fixed in the membrane with 8% acetic acid for 5 min. Samples were washed three times with water, three times with 100% methanol, and then three times with water. A downstream IB analysis was performed normally. Quantification of IBs was performed using ImageJ densitometric gel analysis protocol for 1D gels.

### Complex II in-gel activity assay

Complex II activity was assessed downstream of Native PAGE using an in-gel activity assay as previously described (*61*). Briefly, Native PAGE was performed similar to the BN-PAGE protocol above, except that decreased Coomassie G-250 was used to prevent interference with the colorimetric reaction of the activity assay. Following normal BN-PAGE sample loading, samples were electrophoresed at 150 V for 30 min at 4°C with the light blue cathode buffer (Thermo Fisher Scientific, NP0006). After 30 min, the light blue cathode buffer was replaced with the clear anode buffer and electrophoresis proceeded for an additional 1.5 hours. Following electrophoresis, the gel was removed and transferred to ice-cold ultrapure water. Fresh Complex II activity assay substrate was prepared with the following components: 5 mM tris-HCl (pH 7.4), 20 mM sodium succinate, 4-nitroblue tetrazolium (2.5 mg/ml), and 2 mM phenazine methosulfate in ultrapure water. Ten milliliters of the Complex II substrate was incubated on the gel in a 37°C incubator for 40 min. The reaction was stopped with 10% acetic acid, washed with ultrapure water, and imaged using a Bio-Rad Gel Doc system.

### IFA and confocal fluorescence microscopy

Macrophages were plated on glass coverslips (no. 1.5). Following stimulation, macrophages were fixed at RT with freshly prepared 4% PFA in PBS for 15 min. The IFA was always performed on the same day as the experiment. Coverslips were washed three times with PBS + 0.1% Triton X-100 (IFA wash buffer). Coverslips were blocked with a blocking buffer composed of 5% BSA and 10% normal goat serum in wash buffer. A cocktail of primary antibodies was prepared in blocking buffer and incubated on coverslips for 1 hour at RT. Coverslips were washed three times with IFA wash buffer and incubated with secondary antibodies and counterstains at RT for 30 min. Coverslips were washed and mounted onto microscope slides using Prolong Glass mounting solution. Coverslips were imaged using a Nikon Yokogawa X1-CSU spinning disk confocal microscope equipped with a 100× objective. Operator bias was minimized during image acquisition through selection of fields of view and focal planes based on counterstains that were unrelated to the experimental question. A list of all antibody sources and applications is available in the associated table (table S1).

### Confocal microscopic analysis of mitochondrial superoxide (MitoSOX)

Macrophages were plated on glass-bottom (no. 1.5) Mat-Tek dishes and allowed to adhere overnight. Cells were stimulated with or without LPS for 3 or 6 hours. Within the last 20 min of the experiment, cells were stained with 5 μM MitoSOX dye (Thermo Fisher Scientific, M36008) and Hoechst (1 μg/ml) for 20 min at 37°C, protected from light. Cells were washed with PBS and then fixed with 4% PFA at RT for 15 min. Cells were washed with PBS and then immediately imaged using a Nikon Yokogawa X1-CSU spinning disk confocal microscope. Fields of view were selected on the basis of the Hoechst stain.

### Image analysis, processing, and 3D rendering

Automated image analysis was performed with the open-source software CellProfiler. All CellProfiler pipelines related to this publication are available in the Supplementary Materials (files S1 to S4) and via GitHub. Image analysis was performed on raw images unless otherwise indicated. Automated single-cell analysis was achieved by identification of nuclear objects based on global thresholding of a nuclear stain [e.g., Hoechst or DAPI (4′,6-diamidino-2-phenylindole)] using the identify primary objects module, followed by propagation of the nuclear objects to the cellular periphery based on a whole-cell stain using the identify secondary objects module. Subsequently, a variety of cellular parameters were measured and related to parent cells using the relate objects module. To quantify mitochondrial superoxide (file S1), the intensity of the mitochondrial superoxide indicator MitoSOX was measured within cell objects. To measure SDHB sequestration (file S2), puncta in the SDHB immunostain were identified using the speckles enhancement module and Otsu adaptive thresholding within the identify primary objects module. Three-dimensional rendering of confocal micrographs was performed using Napari and the Allen Cell & Structure Segmenter plugin. Removal of outliers resulting from automated image analysis was performed using strict ROUT outlier identification (*Q* = 0.1%) in GraphPad Prism from cell-level data pooled across multiple experiments. Representative confocal images shown in this manuscript were prepared using the ImageJ background subtraction tool with a rolling ball radius of 30 pixels.

### Cytokine analysis

Macrophages were stimulated with or without LPS (200 ng/ml) for 6 or 24 hours or infected with STM or EC for 24 hours. In some experiments, 1 or 0.1 μM Atpenin A5 (AA5) or 10, 1, or 0.1 mM DMM was added to the culture supernatant for 1 hour before treatment. Following stimulation, culture supernatants were collected, and IL-6, TNF-α, IL-1α, TGF-β, CCL2, CCL3, CCL4, CCL5, CXCL2, and/or CXCL-10 levels in the supernatant were measured by ELISA by the University of Michigan Cancer Center Immunology Core. Pro–IL-1β was detected in the cell lysate by IB (1:1000; R&D Systems, AF-401-SP).

### Flow cytometric analysis of mitochondrial membrane potential

Macrophages were treated with or without LPS (200 ng/ml) for 6 hours or 20 μM of the protonophore carbonyl cyanide 3-chlorophenylhydrazone for 1 hour. After stimulation, cells were stained with 150 nM of the mitochondrial membrane potential dye tetramethylrhodamine methyl ester (TMRM) for 10 min at RT, protected from light. Cells were washed and resuspended in PBS. Cells were immediately analyzed by flow cytometry (Fortessa, BD Biosciences). Data were processed using FlowJo, and the geometric mean intensity of TMRM from each experiment is reported.

### Flow cytometric analysis of bulk mitophagy using the Mito-QC reporter system

BMDMs were generated from the Mito-QC mitophagy reporter mice that harbor recombinant FIS1 protein dual tagged with GFP and mCherry. The Mito-QC mice were originally generated by the laboratory of I. Ganley and were a gift from the laboratory of D. Goldstein. BMDMs were stimulated with LPS (200 ng/ml), starved (20% medium in PBS supplemented with 10 mM glucose), or given fresh medium for 24 hours. Within each condition, BMDMs were stimulated with either Baf A (100 nM) or vehicle control (DMSO). The geometric mean intensity of GFP and mCherry in live cells was measured by flow cytometry (Fortessa, BD Biosciences), and the ratio of these values is reported as a mitophagy index, a method that has been previously described (*25*).

### Mito-QC fixed confocal microscopy

Mito-QC BMDMs were grown overnight in 96-well Perkin Elmer LLC CellCarrier-96 Ultra Microplates (Thermo Fisher Scientific, catalog no. 6055302). On the day of the experiment, BMDMs were stimulated for 24 hours with or without LPS (200 ng/ml) in the presence of Baf A (100 nM) or vehicle control (DMSO). Samples were washed with PBS and fixed with 4% PFA for 15 min at RT. Samples were permeabilized with 0.1% Triton X-100 in PBS. Samples were blocked with 5% BSA and 10% normal goat serum for 30 min. An IFA against SDHB (1:300; Proteintech 10620-1-AP) was performed. Samples were stained with anti-rabbit AF647 (1:500; Thermo Fisher Scientific, A21244) secondary antibody and DAPI at RT for 30 min. Samples were washed and then immediately imaged in PBS using a Nikon Yokogawa X1-CSU Spinning disk confocal microscope. Image quantification was performed using CellProfiler and the analysis pipeline is available in file S3. Briefly, cell area was identified by low threshold segmentation of the mCherry signal. Nuclear signal, based on DAPI segmentation, was subtracted from the SDHB immunostain to eliminate nonspecific nuclear staining present with this antibody (representative images are shown without nuclear subtraction). Colocalization (Mander’s) analysis of SDHB with mCherry or GFP with mCherry is reported. Each data point represents an average of approximately 100 cells per condition derived from four different mice.

### Stable expression of inducible stomatin-like protein-GFP in iBMDM

A pBig2i doxycycline-inducible human SLP2 plasmid was obtained from the laboratory of J. Madrenas (*36*). SLP2-GFP was PCR-amplified from the original plasmid using Q5 polymerase (NEB) with Gibson assembly primers that are available in the Supplementary Materials (table S2). The purified PCR product was then inserted into the Age I/Mlu I digested dox-inducible lentiviral plasmid pTRIPZ using Gibson assembly. Sequence validation was performed by Eurofin to confirm successful subcloning and is available in the Supplementary Materials (data S3). The plasmid was packaged in HEK293T cells using the VSV-G and psPAX2 lentiviral packaging plasmids by the University of Michigan Vector Core. Cells were transduced and selected with puromycin (3 μg/ml) for 1 week. Doxycycline-induced expression was validated by widefield imaging on a Lionheart FX Automated Microscope (BioTek) after 48-hour stimulation with or without doxycycline hyclate (1 μg/ml).

### Two-dimensional SIM analysis of SLP2-GFP and SDHB

CRLS1 KD and NT-Control iBMDM were transduced with the SLP2-GFP pTRIPZ plasmid, selected for a week with puromycin (3 μg/ml) and then induced for 48 hours with doxycycline (1 μg/ml). Following SLP2-GFP induction, iBMDMs were plated on sterile no. 1.5 cover glass and allowed to adhere overnight. On the day of the experiment, iBMDMs were stimulated with or without LPS (200 ng/ml) for 8 hours. Samples were washed with PBS and fixed with 4% PFA for 15 min at RT. Samples were permeabilized with 0.1% Triton X-100 in PBS for 20 min. Samples were blocked with 5% BSA and 10% normal goat serum for 30 min. SDHB (1:300; Abcam, ab14714), TOM20 (1:300; Proteintech, 11802-1-AP), and DAPI staining was performed at RT for 1 hour. Samples were stained with anti-rabbit AF594 (1:500; Thermo Fisher Scientific, A11012) and anti-mouse AF647 (1:500; Thermo Fisher Scientific, A-21235) secondary antibodies at RT for 30 min. Samples were mounted using ProLong Glass mounting reagent (P36980) and cured for 48 hours at RT before imaging. Samples were imaged and reconstructed using 2D SIM (N-SIM). Image quantification was performed using CellProfiler and the analysis pipeline is available in file S4. Segmentation of nonmitochondrial SLP2-GFP and SDHB puncta was performed using Otsu-based adaptive thresholding of TOM20, SLP2-GFP, and SDHB signal, and exclusion of TOM20-overlapped SDHB or SLP2-GFP puncta was accomplished using the relate and filter modules. SLP2-GFP and SDHB double-positive puncta were identified using the relate objects module in CellProfiler. In parallel, colocalization analysis (Pearson’s) between SLP2-GFP, SDHB, and TOM20 was performed.

### Reverse transcription quantitative PCR

RNA was extracted from cells using TRIzol and purified using the Direct-zol RNA MiniPrep Plus kit (Zymo, R2072). The concentration and purity of RNA extracts were determined by NanoDrop. cDNA was synthesized from equivalent amounts of RNA using the iScript cDNA synthesis kit (Bio-Rad, 1708890). RT-qPCR was performed using the SYBR Green system and a Bio-Rad CFX96 Real-Time system. qPCR primers were designed on the basis of the literature or using National Center for Biotechnology Information (NCBI) Primer BLAST. A list of all qPCR primers is available in a separate document (table S2).

## References

[1] L. A. J. O’Neill, R. J. Kishton, J. Rathmell, A guide to immunometabolism for immunologists. Nat. Rev. Immunol. 16, 553–565 (2016). 27396447 10.1038/nri.2016.70 PMC5001910

[2] L. Sun (孙李哲), X. Yang (杨晓峰), Z. Yuan (袁祖贻), H. Wang (王虹), Metabolic reprogramming in immune response and tissue inflammation. Arterioscler. Thromb. Vasc. Biol. 40, 1990–2001 (2020). 32698683 10.1161/ATVBAHA.120.314037 PMC7484156

[3] A. Viola, F. Munari, R. Sánchez-Rodríguez, T. Scolaro, A. Castegna, The metabolic signature of macrophage responses. Front. Immunol. 10, 1462 (2019). 31333642 10.3389/fimmu.2019.01462 PMC6618143

[4] S. K. Wculek, S. C. Khouili, E. Priego, I. Heras-Murillo, D. Sancho, Metabolic control of dendritic cell functions: Digesting information. Frontiers in Immunology. 10, (2019). 10.3389/fimmu.2019.00775 PMC6496459 31073300

[5] N. M. Chapman, H. Chi, Metabolic adaptation of lymphocytes in immunity and disease. Immunity 55, 14–30 (2022). 35021054 10.1016/j.immuni.2021.12.012 PMC8842882

[6] B. Kelly, L. A. J. O’Neill, Metabolic reprogramming in macrophages and dendritic cells in innate immunity. Cell Res. 25, 771–784 (2015). 26045163 10.1038/cr.2015.68 PMC4493277

[7] Y. Liu, R. Xu, H. Gu, E. Zhang, J. Qu, W. Cao, X. Huang, H. Yan, J. He, Z. Cai, Metabolic reprogramming in macrophage responses. Biomark Res. 9, 1 (2021). 33407885 10.1186/s40364-020-00251-y PMC7786975

[8] A. Chawla, K. D. Nguyen, Y. P. S. Goh, Macrophage-mediated inflammation in metabolic disease. Nat. Rev. Immunol. 11, 738–749 (2011). 21984069 10.1038/nri3071 PMC3383854

[9] E. L. Mills, B. Kelly, A. Logan, A. S. H. Costa, M. Varma, C. E. Bryant, P. Tourlomousis, J. H. M. Däbritz, E. Gottlieb, I. Latorre, S. C. Corr, G. McManus, D. Ryan, H. T. Jacobs, M. Szibor, R. J. Xavier, T. Braun, C. Frezza, M. P. Murphy, L. A. O’Neill, Succinate dehydrogenase supports metabolic repurposing of mitochondria to drive inflammatory macrophages. Cell. 167, 457–470.e13 (2016). 27667687 10.1016/j.cell.2016.08.064 PMC5863951

[10] G. M. Tannahill, A. M. Curtis, J. Adamik, E. M. Palsson-McDermott, A. F. McGettrick, G. Goel, C. Frezza, N. J. Bernard, B. Kelly, N. H. Foley, L. Zheng, A. Gardet, Z. Tong, S. S. Jany, S. C. Corr, M. Haneklaus, B. E. Caffrey, K. Pierce, S. Walmsley, F. C. Beasley, E. Cummins, V. Nizet, M. Whyte, C. T. Taylor, H. Lin, S. L. Masters, E. Gottlieb, V. P. Kelly, C. Clish, P. E. Auron, R. J. Xavier, L. A. J. O’Neill, Succinate is an inflammatory signal that induces IL-1β through HIF-1α. Nature 496, 238–242 (2013). 23535595 10.1038/nature11986 PMC4031686

[11] J. K. Dowling, R. Afzal, L. J. Gearing, M. P. Cervantes-Silva, S. Annett, G. M. Davis, C. De Santi, N. Assmann, K. Dettmer, D. J. Gough, G. R. Bantug, F. I. Hamid, F. K. Nally, C. P. Duffy, A. L. Gorman, A. M. Liddicoat, E. C. Lavelle, C. Hess, P. J. Oefner, D. K. Finlay, G. P. Davey, T. Robson, A. M. Curtis, P. J. Hertzog, B. R. G. Williams, C. E. McCoy, Mitochondrial arginase-2 is essential for IL-10 metabolic reprogramming of inflammatory macrophages. Nat. Commun. 12, 1460 (2021). 33674584 10.1038/s41467-021-21617-2 PMC7936006

[12] J. Garaude, R. Acín-Pérez, S. Martínez-Cano, M. Enamorado, M. Ugolini, E. Nistal-Villán, S. Hervás-Stubbs, P. Pelegrín, L. E. Sander, J. A. Enríquez, D. Sancho, Mitochondrial respiratory-chain adaptations in macrophages contribute to antibacterial host defense. Nat. Immunol. 17, 1037–1045 (2016). 27348412 10.1038/ni.3509 PMC4994870

[13] H. Zuo, Y. Wan, Metabolic reprogramming in mitochondria of myeloid cells. Cells 9, 5 (2019). 31861356 10.3390/cells9010005 PMC7017304

[14] E. M. Palmieri, M. Gonzalez-Cotto, W. A. Baseler, L. C. Davies, B. Ghesquière, N. Maio, C. M. Rice, T. A. Rouault, T. Cassel, R. M. Higashi, A. N. Lane, T. W.-M. Fan, D. A. Wink, D. W. McVicar, Nitric oxide orchestrates metabolic rewiring in M1 macrophages by targeting aconitase 2 and pyruvate dehydrogenase. Nat. Commun. 11, 698 (2020). 32019928 10.1038/s41467-020-14433-7 PMC7000728

[15] V. Lampropoulou, A. Sergushichev, M. Bambouskova, S. Nair, E. E. Vincent, E. Loginicheva, L. Cervantes-Barragan, X. Ma, S. C.-C. Huang, T. Griss, C. J. Weinheimer, S. Khader, G. J. Randolph, E. J. Pearce, R. G. Jones, A. Diwan, M. S. Diamond, M. N. Artyomov, Itaconate links inhibition of succinate dehydrogenase with macrophage metabolic remodeling and regulation of inflammation. Cell Metab. 24, 158–166 (2016). 27374498 10.1016/j.cmet.2016.06.004 PMC5108454

[16] J. Dudek, Role of cardiolipin in mitochondrial signaling pathways. Front Cell Dev. Biol. 5, 90 (2017). 29034233 10.3389/fcell.2017.00090 PMC5626828

[17] G. Tasseva, H. D. Bai, M. Davidescu, A. Haromy, E. Michelakis, J. E. Vance, Phosphatidylethanolamine deficiency in mammalian mitochondria impairs oxidative phosphorylation and alters mitochondrial morphology. J. Biol. Chem. 288, 4158–4173 (2013). 23250747 10.1074/jbc.M112.434183 PMC3567666

[18] J. Dudek, M. Hartmann, P. Rehling, The role of mitochondrial cardiolipin in heart function and its implication in cardiac disease. Biochim. Biophys. Acta Mol. Basis Dis. 1865, 810–821 (2019). 30837070 10.1016/j.bbadis.2018.08.025

[19] G. C. Hard, Some biochemical aspects of the immune macrophage. Br. J. Exp. Pathol. 51, 97–105 (1970). 5434449 PMC2072214

[20] P. Newsholme, S. Gordon, E. A. Newsholme, Rates of utilization and fates of glucose, glutamine, pyruvate, fatty acids and ketone bodies by mouse macrophages. Biochem. J. 242, 631–636 (1987). 3593269 10.1042/bj2420631 PMC1147758

[21] N. V. Dudkina, R. Kouril, K. Peters, H.-P. Braun, E. J. Boekema, Structure and function of mitochondrial supercomplexes. Biochim. Biophys. Acta. 1797, 664–670 (2010). 20036212 10.1016/j.bbabio.2009.12.013

[22] Y. Kageyama, M. Hoshijima, K. Seo, D. Bedja, P. Sysa-Shah, S. A. Andrabi, W. Chen, A. Höke, V. L. Dawson, T. M. Dawson, K. Gabrielson, D. A. Kass, M. Iijima, H. Sesaki, Parkin-independent mitophagy requires Drp1 and maintains the integrity of mammalian heart and brain. EMBO J. 33, 2798–2813 (2014). 25349190 10.15252/embj.201488658 PMC4282557

[23] F. Gao, M. B. Reynolds, K. D. Passalacqua, J. Z. Sexton, B. H. Abuaita, M. X. D. O’Riordan, The mitochondrial fission regulator DRP1 controls post-transcriptional regulation of TNF-α. Front. Cell. Infect. Microbiol. 10, 593805 (2021). 33520735 10.3389/fcimb.2020.593805 PMC7840702

[24] K. Palikaras, E. Lionaki, N. Tavernarakis, Mechanisms of mitophagy in cellular homeostasis, physiology and pathology. Nat. Cell Biol. 20, 1013–1022 (2018). 30154567 10.1038/s41556-018-0176-2

[25] T. G. McWilliams, A. R. Prescott, G. F. G. Allen, J. Tamjar, M. J. Munson, C. Thomson, M. M. K. Muqit, I. G. Ganley, mito-QC illuminates mitophagy and mitochondrial architecture in vivo. J. Cell Biol. 214, 333–345 (2016). 27458135 10.1083/jcb.201603039 PMC4970326

[26] Y. Hirota, S.-I. Yamashita, Y. Kurihara, X. Jin, M. Aihara, T. Saigusa, D. Kang, T. Kanki, Mitophagy is primarily due to alternative autophagy and requires the MAPK1 and MAPK14 signaling pathways. Autophagy 11, 332–343 (2015). 25831013 10.1080/15548627.2015.1023047 PMC4502654

[27] V. E. Kagan, J. Jiang, Z. Huang, Y. Y. Tyurina, C. Desbourdes, C. Cottet-Rousselle, H. H. Dar, M. Verma, V. A. Tyurin, A. A. Kapralov, A. Cheikhi, G. Mao, D. Stolz, C. M. St Croix, S. Watkins, Z. Shen, Y. Li, M. L. Greenberg, M. Tokarska-Schlattner, M. Boissan, M.-L. Lacombe, R. M. Epand, C. T. Chu, R. K. Mallampalli, H. Bayır, U. Schlattner, NDPK-D (NM23-H4)-mediated externalization of cardiolipin enables elimination of depolarized mitochondria by mitophagy. Cell Death Differ. 23, 1140–1151 (2016). 26742431 10.1038/cdd.2015.160 PMC4946882

[28] S. S. Iyer, Q. He, J. R. Janczy, E. I. Elliott, Z. Zhong, A. K. Olivier, J. J. Sadler, V. Knepper-Adrian, R. Han, L. Qiao, S. C. Eisenbarth, W. M. Nauseef, S. L. Cassel, F. S. Sutterwala, Mitochondrial cardiolipin is required for Nlrp3 inflammasome activation. Immunity 39, 311–323 (2013). 23954133 10.1016/j.immuni.2013.08.001 PMC3779285

[29] Y.-W. Lu, S. M. Claypool, Disorders of phospholipid metabolism: An emerging class of mitochondrial disease due to defects in nuclear genes. Front. Genet. 6, 3 (2015). 25691889 10.3389/fgene.2015.00003 PMC4315098

[30] C. T. Schwall, V. L. Greenwood, N. N. Alder, The stability and activity of respiratory Complex II is cardiolipin-dependent. Biochim. Biophys. Acta. 1817, 1588–1596 (2012). 22575443 10.1016/j.bbabio.2012.04.015

[31] O. Ernst, J. Sun, B. Lin, B. Banoth, M. G. Dorrington, J. Liang, B. Schwarz, K. A. Stromberg, S. Katz, S. J. Vayttaden, C. J. Bradfield, N. Slepushkina, C. M. Rice, E. Buehler, J. S. Khillan, D. W. McVicar, C. M. Bosio, C. E. Bryant, F. S. Sutterwala, S. E. Martin, M. Lal-Nag, I. D. C. Fraser, A genome-wide screen uncovers multiple roles for mitochondrial nucleoside diphosphate kinase D in inflammasome activation. Sci. Signal. 14, (2021). 10.1126/scisignal.abe0387 PMC7613020 34344832

[32] D. G. Ryan, L. A. J. O’Neill, Krebs cycle reborn in macrophage immunometabolism. Annu. Rev. Immunol. 38, 289–313 (2020). 31986069 10.1146/annurev-immunol-081619-104850

[33] Y. Huang, C. Powers, S. K. Madala, K. D. Greis, W. D. Haffey, J. A. Towbin, E. Purevjav, S. Javadov, A. W. Strauss, Z. Khuchua, Cardiac metabolic pathways affected in the mouse model of barth syndrome. PLOS ONE 10, e0128561 (2015). 26030409 10.1371/journal.pone.0128561 PMC4451073

[34] Y.-W. Lu, L. Galbraith, J. D. Herndon, Y.-L. Lu, M. Pras-Raves, M. Vervaart, A. Van Kampen, A. Luyf, C. M. Koehler, J. M. McCaffery, E. Gottlieb, F. M. Vaz, S. M. Claypool, Defining functional classes of Barth syndrome mutation in humans. Hum. Mol. Genet. 25, 1754–1770 (2016). 26908608 10.1093/hmg/ddw046 PMC4986330

[35] D. A. Christie, C. D. Lemke, I. M. Elias, L. A. Chau, M. G. Kirchhof, B. Li, E. H. Ball, S. D. Dunn, G. M. Hatch, J. Madrenas, Stomatin-like protein 2 binds cardiolipin and regulates mitochondrial biogenesis and function. Mol. Cell. Biol. 31, 3845–3856 (2011). 21746876 10.1128/MCB.05393-11 PMC3165718

[36] D. A. Christie, M. G. Kirchhof, S. Vardhana, M. L. Dustin, J. Madrenas, Mitochondrial and plasma membrane pools of stomatin-like protein 2 coalesce at the immunological synapse during T cell activation. PLOS ONE 7, e37144 (2012). 22623988 10.1371/journal.pone.0037144 PMC3356372

[37] W. van der Stel, H. Yang, N. G. Vrijenhoek, J. P. Schimming, G. Callegaro, G. Carta, S. Darici, J. Delp, A. Forsby, A. White, S. le Dévédec, M. Leist, P. Jennings, J. B. Beltman, B. van de Water, E. H. J. Danen, Mapping the cellular response to electron transport chain inhibitors reveals selective signaling networks triggered by mitochondrial perturbation. Arch. Toxicol. 96, 259–285 (2022). 34642769 10.1007/s00204-021-03160-7 PMC8748354

[38] M. Yin, L. A. J. O’Neill, The role of the electron transport chain in immunity. FASEB J. 35, e21974 (2021). 34793601 10.1096/fj.202101161R

[39] S. Chen, J. Yang, Y. Wei, X. Wei, Epigenetic regulation of macrophages: From homeostasis maintenance to host defense. Cell. Mol. Immunol. 17, 36–49 (2020). 31664225 10.1038/s41423-019-0315-0 PMC6952359

[40] F. Humphries, L. Shmuel-Galia, N. Ketelut-Carneiro, S. Li, B. Wang, V. V. Nemmara, R. Wilson, Z. Jiang, F. Khalighinejad, K. Muneeruddin, S. A. Shaffer, R. Dutta, C. Ionete, S. Pesiridis, S. Yang, P. R. Thompson, K. A. Fitzgerald, Succination inactivates gasdermin D and blocks pyroptosis. Science 369, 1633–1637 (2020). 32820063 10.1126/science.abb9818 PMC8744141

[41] M. Canton, R. Sánchez-Rodríguez, I. Spera, F. C. Venegas, M. Favia, A. Viola, A. Castegna, Reactive oxygen species in macrophages: Sources and targets. Front. Immunol. 12, 734229 (2021). 34659222 10.3389/fimmu.2021.734229 PMC8515906

[42] A. Bezawork-Geleta, H. Wen, L. Dong, B. Yan, J. Vider, S. Boukalova, L. Krobova, K. Vanova, R. Zobalova, M. Sobol, P. Hozak, S. M. Novais, V. Caisova, P. Abaffy, R. Naraine, Y. Pang, T. Zaw, P. Zhang, R. Sindelka, M. Kubista, S. Zuryn, M. P. Molloy, M. V. Berridge, K. Pacak, J. Rohlena, S. Park, J. Neuzil, Alternative assembly of respiratory complex II connects energy stress to metabolic checkpoints. Nat. Commun. 9, 2221 (2018). 29880867 10.1038/s41467-018-04603-z PMC5992162

[43] L. K. Billingham, J. S. Stoolman, K. Vasan, A. E. Rodriguez, T. A. Poor, M. Szibor, H. T. Jacobs, C. R. Reczek, A. Rashidi, P. Zhang, J. Miska, N. S. Chandel, Mitochondrial electron transport chain is necessary for NLRP3 inflammasome activation. Nat. Immunol. 23, 692–704 (2022). 35484407 10.1038/s41590-022-01185-3 PMC9098388

[44] S. A. Clayton, K. K. Daley, L. MacDonald, E. Fernandez-Vizarra, G. Bottegoni, J. D. O’Neil, T. Major, D. Griffin, Q. Zhuang, A. B. Adewoye, K. Woolcock, S. W. Jones, C. Goodyear, A. Elmesmari, A. Filer, D. A. Tennant, S. Alivernini, C. D. Buckley, R. D. S. Pitceathly, M. Kurowska-Stolarska, A. R. Clark, Inflammation causes remodeling of mitochondrial cytochrome *c* oxidase mediated by the bifunctional gene *C15orf48*. Sci Adv. 7, eabl5182 (2021). 34878835 10.1126/sciadv.abl5182 PMC8654286

[45] B. G. Slane, N. Aykin-Burns, B. J. Smith, A. L. Kalen, P. C. Goswami, F. E. Domann, D. R. Spitz, Mutation of succinate dehydrogenase subunit C results in increased O_2_^·−^, oxidative stress, and genomic instability. Cancer Res. 66, 7615–7620 (2006). 16885361 10.1158/0008-5472.CAN-06-0833

[46] R. D. Guzy, B. Sharma, E. Bell, N. S. Chandel, P. T. Schumacker, Loss of the SdhB, but Not the SdhA, subunit of complex II triggers reactive oxygen species-dependent hypoxia-inducible factor activation and tumorigenesis. Mol. Cell. Biol. 28, 718–731 (2008). 17967865 10.1128/MCB.01338-07 PMC2223429

[47] B. E. Baysal, R. E. Ferrell, J. E. Willett-Brozick, E. C. Lawrence, D. Myssiorek, A. Bosch, A. van der Mey, P. E. M. Taschner, W. S. Rubinstein, E. N. Myers, C. W. Richard, C. J. Cornelisse, P. Devilee, B. Devlin, Mutations in *SDHD*, a mitochondrial complex II gene, in hereditary paraganglioma. Science 287, 848–851 (2000). 10657297 10.1126/science.287.5454.848

[48] S. Lee, H. Xu, A. Van Vleck, A. M. Mawla, A. M. Li, J. Ye, M. O. Huising, J. P. Annes, β-Cell succinate dehydrogenase deficiency triggers metabolic dysfunction and insulinopenic diabetes. Diabetes 71, 1439–1453 (2022). 35472723 10.2337/db21-0834 PMC9233299

[49] K. R. Pryde, J. W. Taanman, A. H. Schapira, A LON-ClpP proteolytic axis degrades complex I to extinguish ROS production in depolarized mitochondria. Cell Rep. 17, 2522–2531 (2016). 27926857 10.1016/j.celrep.2016.11.027 PMC5177631

[50] I. Martínez-Reyes, N. S. Chandel, Mitochondrial TCA cycle metabolites control physiology and disease. Nat. Commun. 11, 102 (2020). 31900386 10.1038/s41467-019-13668-3 PMC6941980

[51] P. Jadiya, D. Tomar, Mitochondrial protein quality control mechanisms. Genes 11, (2020). 10.3390/genes11050563 PMC7290828 32443488

[52] R. Kapetanovic, S. F. Afroz, D. Ramnath, G. M. Lawrence, T. Okada, J. E. Curson, J. de Bruin, D. P. Fairlie, K. Schroder, J. C. St John, A. Blumenthal, M. J. Sweet, Lipopolysaccharide promotes Drp1-dependent mitochondrial fission and associated inflammatory responses in macrophages. Immunol. Cell Biol. 98, 528–539 (2020). 32686869 10.1111/imcb.12363 PMC7497224

[53] M. Schlame, Y. Xu, M. Ren, The basis for acyl specificity in the Tafazzin reaction. J. Biol. Chem. 292, 5499–5506 (2017). 28202545 10.1074/jbc.M116.769182 PMC5392692

[54] S. M. Roberson, W. S. Walker, Immortalization of cloned mouse splenic macrophages with a retrovirus containing the v-raf/mil and v-myc oncogenes. Cell. Immunol. 116, 341–351 (1988). 2460250 10.1016/0008-8749(88)90236-5

[55] V. Hornung, F. Bauernfeind, A. Halle, E. O. Samstad, H. Kono, K. L. Rock, K. A. Fitzgerald, E. Latz, Silica crystals and aluminum salts activate the NALP3 inflammasome through phagosomal destabilization. Nat. Immunol. 9, 847–856 (2008). 18604214 10.1038/ni.1631 PMC2834784

[56] D. De Nardo, D. V. Kalvakolanu, E. Latz, Immortalization of murine bone marrow-derived macrophages. Methods Mol. Biol. 1784, 35–49 (2018). 29761386 10.1007/978-1-4939-7837-3_4

[57] D. A. Kulpa, N. Del Cid, K. A. Peterson, K. L. Collins, Adaptor protein 1 promotes cross-presentation through the same tyrosine signal in major histocompatibility complex class I as that targeted by HIV-1. J. Virol. 87, 8085–8098 (2013). 23678182 10.1128/JVI.00701-13 PMC3700188

[58] M. J. Kim, J. W. Hwang, C.-K. Yun, Y. Lee, Y.-S. Choi, Delivery of exogenous mitochondria via centrifugation enhances cellular metabolic function. Sci. Rep. 8, 3330 (2018). 29463809 10.1038/s41598-018-21539-y PMC5820364

[59] C. J. Halbrook, C. Pontious, I. Kovalenko, L. Lapienyte, S. Dreyer, H.-J. Lee, G. Thurston, Y. Zhang, J. Lazarus, P. Sajjakulnukit, H. S. Hong, D. M. Kremer, B. S. Nelson, S. Kemp, L. Zhang, D. Chang, A. Biankin, J. Shi, T. L. Frankel, H. C. Crawford, J. P. Morton, M. Pasca di Magliano, C. A. Lyssiotis, Macrophage-released pyrimidines inhibit gemcitabine therapy in pancreatic cancer. Cell Metab. 29, 1390–1399.e6 (2019). 30827862 10.1016/j.cmet.2019.02.001 PMC6602533

[60] H.-J. Lee, D. M. Kremer, P. Sajjakulnukit, L. Zhang, C. A. Lyssiotis, A large-scale analysis of targeted metabolomics data from heterogeneous biological samples provides insights into metabolite dynamics. Metabolomics 15, 103 (2019). 31289941 10.1007/s11306-019-1564-8 PMC6616221

[61] P. Jha, X. Wang, J. Auwerx, Analysis of mitochondrial respiratory chain supercomplexes using blue native polyacrylamide gel electrophoresis (BN-PAGE). Curr. Protoc. Mouse Biol. 6, 1–14 (2016). 26928661 10.1002/9780470942390.mo150182 PMC4823378

