# Supplementary material for: Cardiolipin coordinates inflammatory metabolic reprogramming through regulation of Complex II disassembly and degradation
Source: Sci Adv. 2023 Feb 3;9(5):eade8701. doi: 10.1126/sciadv.ade8701 (PMC9897665; doi:10.1126/sciadv.ade8701)
Supplement: Supplementary file 1 — Figs. S1 to S21 Tables S1 and S2 Data S3 [file sciadv.ade8701_sm.pdf]

Science Advances
AAAS

Supplementary Materials for

Cardiolipin coordinates inflammatory metabolic reprogramming through regulation of Complex II disassembly and degradation

Mack B. Reynolds *et al.*

Corresponding author: Mary X. O'Riordan, oriordan@umich.edu

Sci. Adv. **9**, eade8701 (2023)
DOI: 10.1126/sciadv.ade8701

The PDF file includes:

Figs. S1 to S21
Tables S1 and S2
Data S3
Legends for data S1 and S2
Legends for files S1 to S4

Other Supplementary Material for this manuscript includes the following:

Data S1 and S2
Files S1 to S4

Supplementary materials:

Supplemental Figures 1-21

Table 1: Antibodies and chemicals used in this study

Table 2: Primer and shRNA sequences used in this study

Data S1: Untargeted lipidomics dataset including normalization and data analysis relevant to this study

Data S2: Targeted metabolomics dataset including data analysis relevant to this study

Data S3: SLP2-GFP pTRIPZ plasmid map and full sequence

File S1: CellProfiler image analysis pipeline 1: Confocal analysis of MitoSOX intensity per cell

File S2: CellProfiler image analysis pipeline 2: Confocal analysis of SDHB puncta per cell

File S3: CellProfiler image analysis pipeline 3: Confocal analysis of MitoQC reporter

File S4: CellProfiler image analysis pipeline 4: SIM analysis of SDHB and SLP2-GFP puncta

Fig. S1 Effect of LPS stimulation on oxygen consumption and extracellular acidification in immortalized bone marrow-derived macrophages (iBMDM)

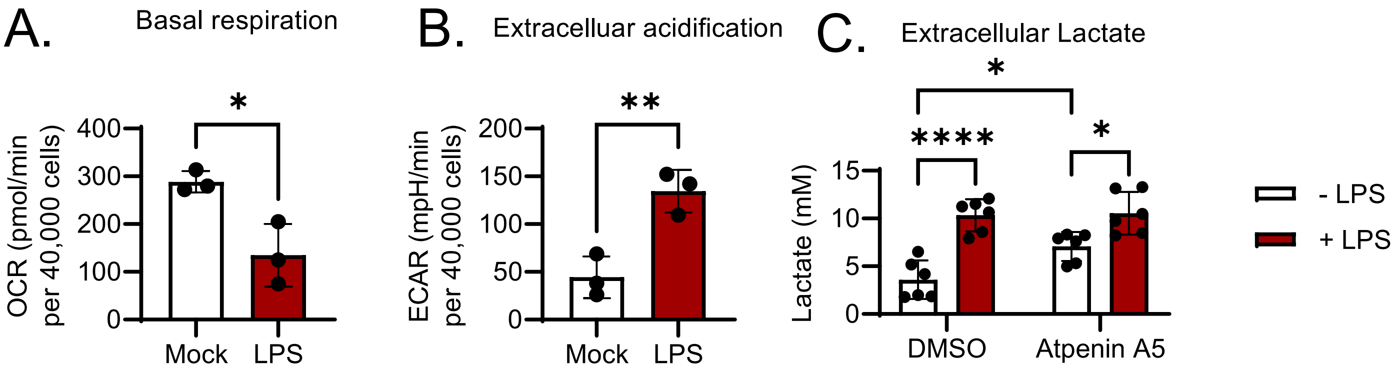

Fig. S1 Effect of LPS stimulation on oxygen consumption and extracellular acidification in immortalized bone marrow-derived macrophages (iBMDM). iBMDM were pretreated with LPS (200 ng/mL) for 6h and then subject to the Agilent Seahorse Extracellular Flux (XF) analysis of the oxygen consumption rate (OCR) and the extracellular acidification rate (ECAR) (**A** and **B**). **C**. Quantification of extracellular lactate as a readout of glycolysis in iBMDM stimulated for 8h with combinations of LPS (200 ng/mL), Complex II inhibitor (Atpenin A5, 1 μM), or vehicle control (0.1% DMSO). Graphs are presented as the mean of $n \geq 3$ independent experiments with standard deviation (SD) error bars. P values were calculated using an unpaired T-test or two-way ANOVA with Sidak's post-test for multiple comparisons. *$P < 0.05$; **$P < 0.01$; and ****$P < 0.0001$.

Fig. S2 Quantification of NDUFB8, ATP5A, and UQCRC2 abundance in LPS stimulated iBMDM.

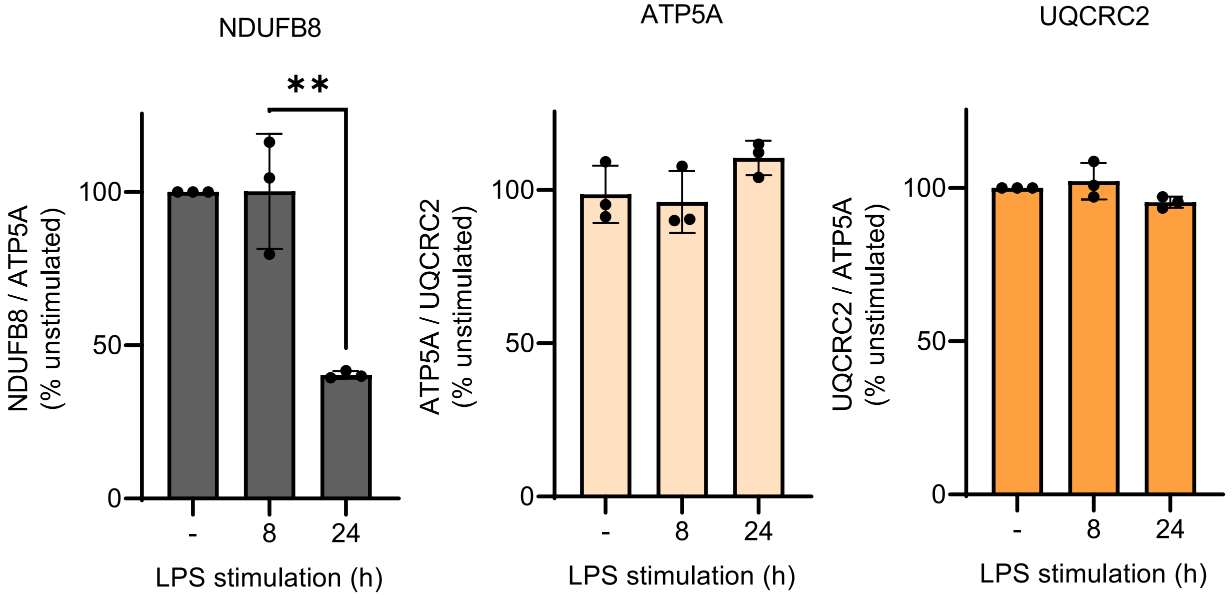

Fig. S2 Quantification of NDUFB8, ATP5A, and UQCRC2 abundance in LPS stimulated iBMDM. Quantification of NDUFB8, ATP5A, and UQCRC2 from Fig. 1A where iBMDM were synchronously stimulated with or without 200 ng/mL LPS for 8 or 24h. NDUFB8 and UQCRC2 are quantified relative to ATP5A which does not change during LPS stimulation. ATP5A was quantified relative to UQCRC2 which also does not change during LPS stimulation. Graphs are presented as the mean of $n = 3$ independent experiments with standard deviation (SD) error bars. P values were calculated using a one-way ANOVA with Tukey's post-test for multiple comparisons. **$P < 0.01$

Fig. S3 Effect of LPS on SDHB in primary murine bone marrow-derived macrophages and primary human monocyte-derived macrophages.

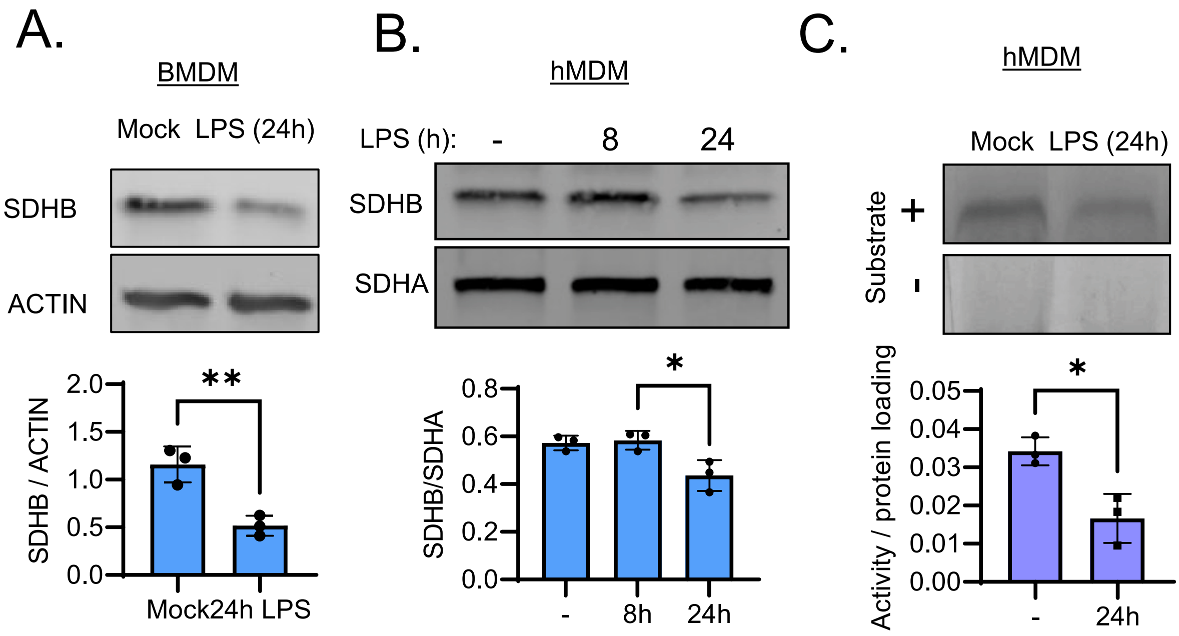

Fig. S3 Effect of LPS on SDHB in primary murine bone marrow-derived macrophages and primary human monocyte-derived macrophages. A. Quantification of SDHB relative to ACTIN in primary bone marrow-derived macrophages (BMDM) stimulated +/- 200 ng/mL LPS for 24h. **B.** Primary human monocyte-derived macrophages (hMDM) were stimulated with or without 200 ng/mL LPS for 8 or 24h. Whole cell extracts were subject to SDS-PAGE and SDHA/SDHB immunoblot. The ratio between SDHB and SDHA was quantified. **C.** Complex II succinate dehydrogenase activity was measured as in Fig. 1F-G from hMDM stimulated with or without LPS for 24h. Graphs are presented as the mean of n = 3 independent experiments from 3 different healthy donors with standard deviation (SD) error bars. P values were calculated using an unpaired T-test or one-way ANOVA with Tukey's post-test for multiple comparisons. *$P < 0.05$ and **$P < 0.01$

Fig. S4 BN-PAGE analysis of Respiratory Complexes I, II, III, IV, and V during LPS stimulation.

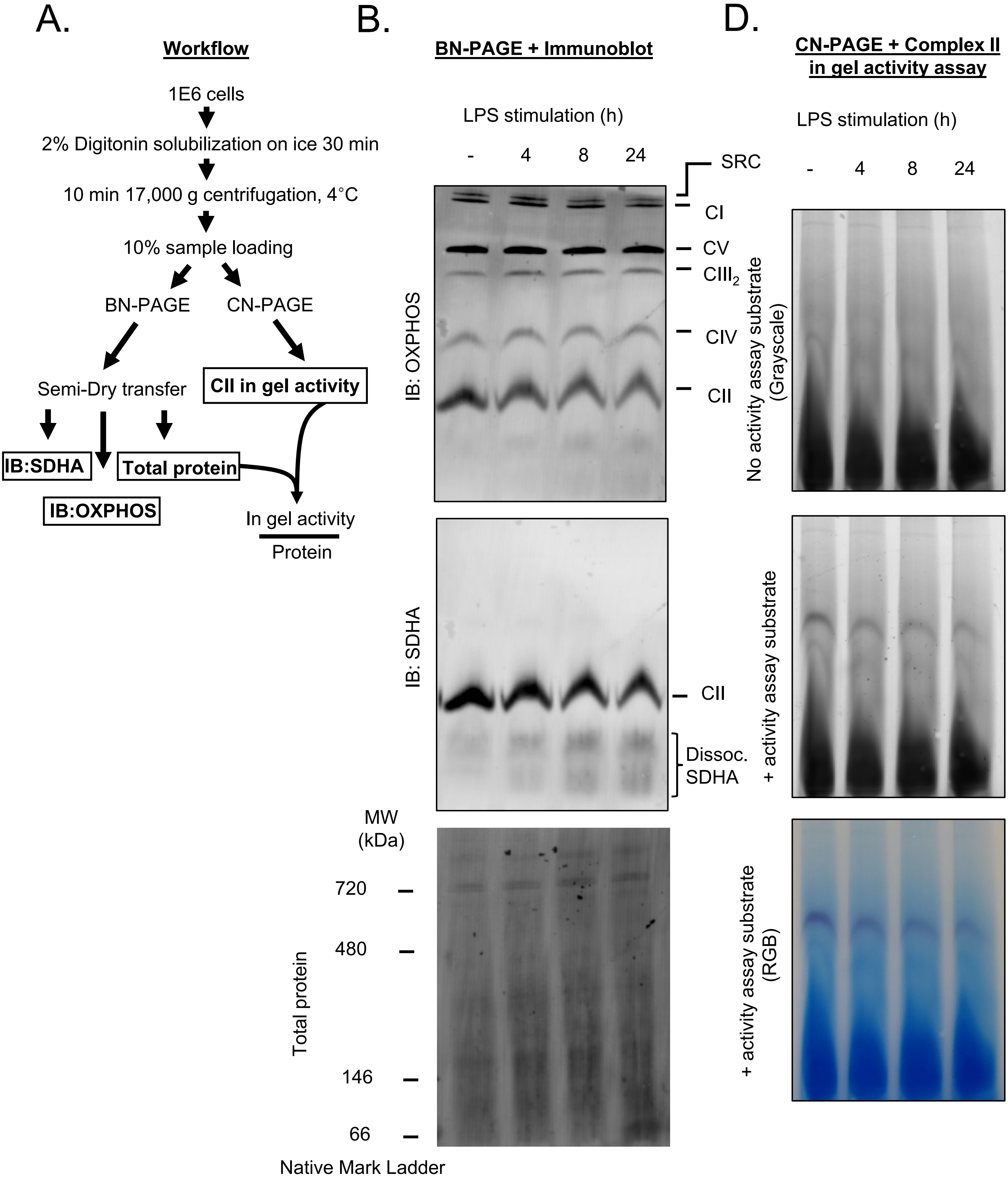

Fig. S4 BN-PAGE analysis of Respiratory Complexes I, II, III, IV, and V during LPS stimulation. A. Workflow of parallel native (BN PAGE) respiratory complex immunoblot and Complex II in gel activity assays shown. OXPHOS immunoblot (cocktail of NDUFB8, SDHB, UQCRC2, MTCO1, and ATPA5) and SDHA immunoblot (**B**) paired to in gel activity assay (**C**) shown in Fig 1F. As indicated in the analysis workflow, total protein stain (Coomassie) was used as normalization for in gel activity.

Fig. S5 Splenocyte SDHB levels during in vivo endotoxemia.

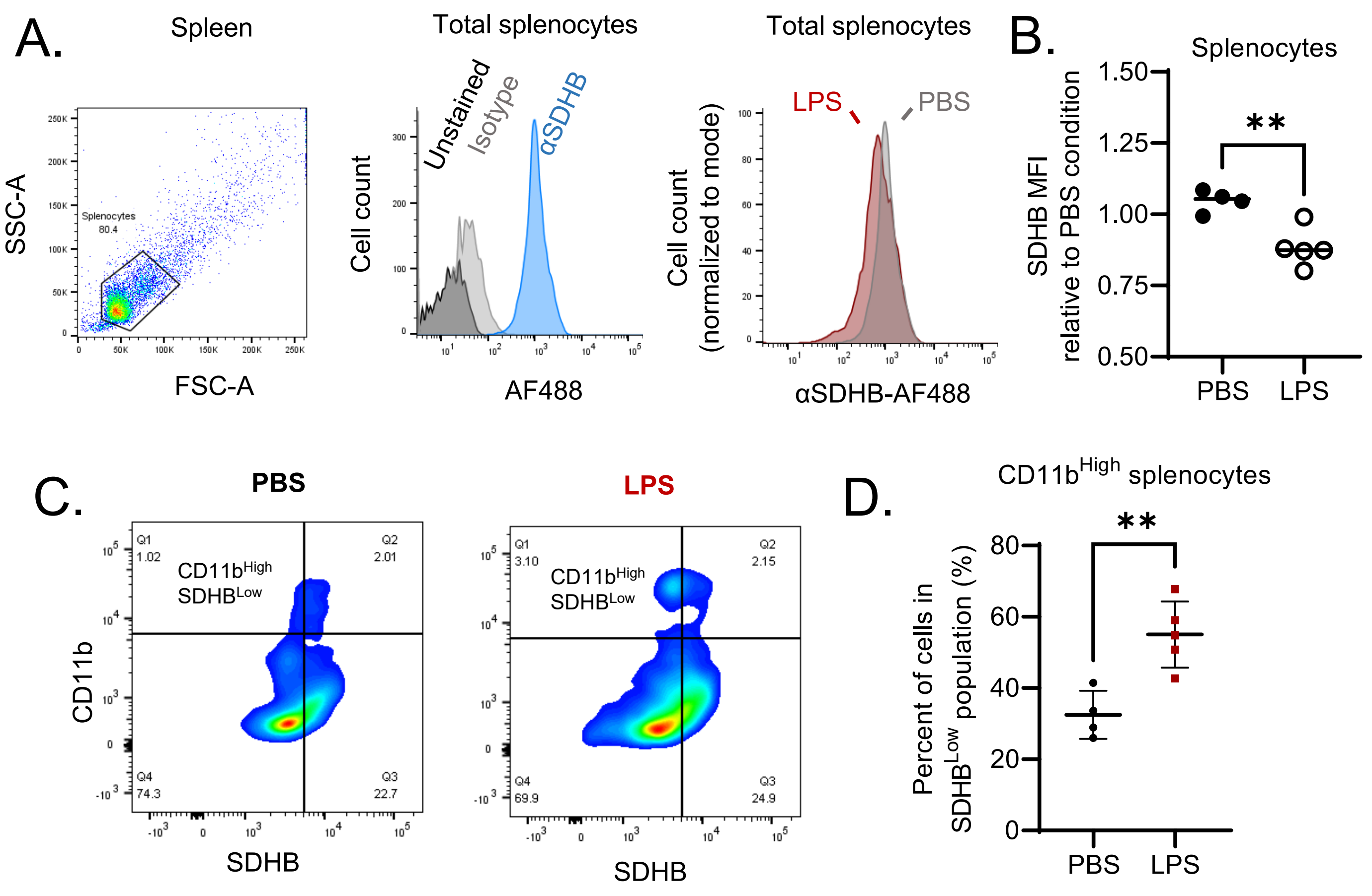

Fig. S5 Splenocyte SDHB levels during *in vivo* endotoxemia. A. Flow cytometric analysis of RBC-lysed PFA-fixed splenocytes with gating in FlowJo to remove debris and cell aggregates. Total splenocyte intracellular staining with anti-SDHB antibody or isotype control and AF488 secondary antibody or left unstained. Representative histogram showing SDHB staining in total splenocytes compared between 24h post 20 mg/kg LPS I.P. injection or PBS I.P. injection. **B.** Quantification of the relative mean fluorescence intensity (MFI) compared to the average MFI of total splenocytes in the PBS injection condition. **C.** Density plots of CD11b expression vs. SDHB abundance in splenocytes from PBS or LPS injected mice. Quadrant gating to identify low vs. high SDHB abundance in CD11b highly expressing splenocytes ($CD11b^{High}$). The SDHB intensity threshold was set at the MFI of total splenocytes in the PBS-injected condition and values to the left of this line are designated $SDHB^{Low}$. **D.** Calculation of the percent of $CD11b^{High}$ splenocytes designated as $SDHB^{Low}$. Graphs are presented as the mean of n=4 (PBS) or n=5 (LPS) mice with standard deviation (SD) error bars. P values were calculated using an unpaired T-test. **$P < 0.01$

Fig. S6 Complex II and SDHB levels in *Salmonella* Typhimurium-infected iBMDM.

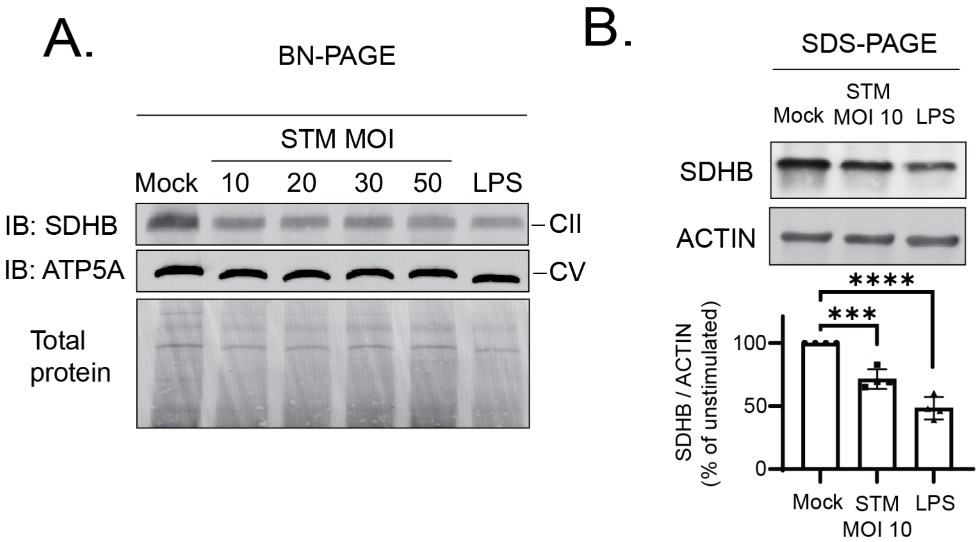

Fig. S6 Complex II and SDHB levels in *Salmonella* Typhimurium-infected iBMDM. A. WT iBMDM were infected with *Salmonella enterica* serovar Typhimurium (STM) at a multiplicity of infection (MOI) of 10, 20, 30, or 50 or stimulated with 200 ng/mL LPS for 24h. Samples were analyzed by BN-PAGE and immunoblot against SDHB and ATP5A to visualize Complex II and Complex V, respectively. Total protein indicates Coomassie staining of the PVDF membrane. **B.** WT iBMDM were infected with STM at a MOI of 10 or treated with 200 ng/mL LPS for 24h and then analyzed by SDS-PAGE and immunoblot against SDHB and ACTIN. The ratio of SDHB/ACTIN as a percentage of the mock-treated sample was calculated for each condition. Fig. S6A is representative of n = 2 independent experiments. The graph in Fig S6B is presented as the mean of n = 4 independent experiments with standard deviation (SD) error bars. P values were calculated using a one-way ANOVA with Tukey's post-test for multiple comparisons. ***$P < 0.001$ and ****$P < 0.0001$.

Fig. S7 BN-PAGE analysis of Complex II in Nos2-/- and Acod1-/- primary BMDM

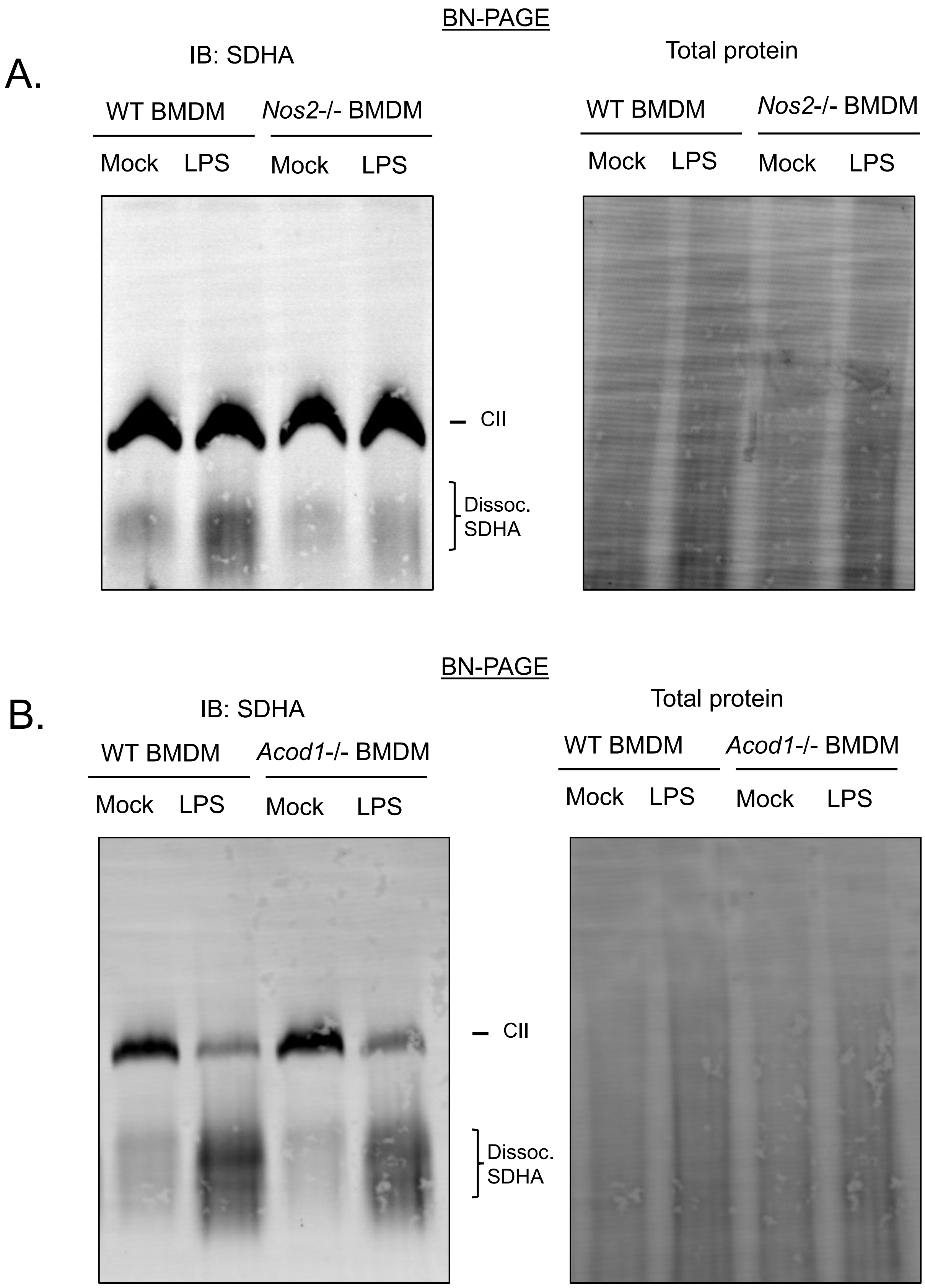

Fig. S7 BN-PAGE analysis of Complex II in *Nos2*-/- and *Acod1*-/- primary BMDM. Bone marrow-derived macrophages generated from *Nos2*-/- (**A.**) or *Acod1*-/- (**B**.) mice and WT littermate controls were stimulated with 200 ng/mL LPS for 24h. Samples were analyzed by BN-PAGE and immunoblot against SDHA. Total protein indicates coomassie staining of the PVDF membrane. Blots are representative of n = 2 independent experiments.

Fig. S8 SDHA and SDHB localization in iBMDM and image analysis workflow.

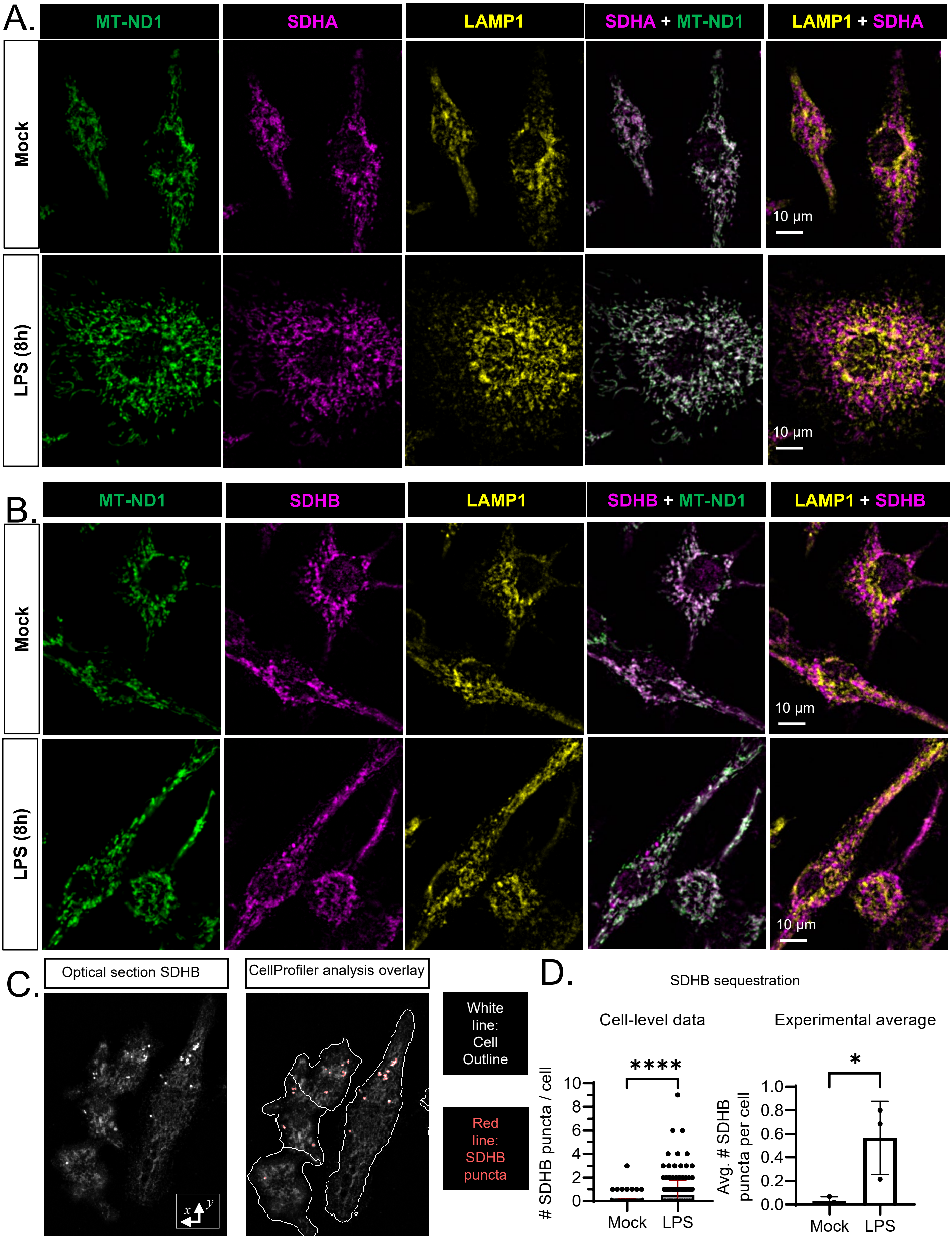

Fig. S8 SDHA and SDHB localization in iBMDM and image analysis workflow. Representative confocal fluorescence micrographs from iBMDM stimulated with or without 200 ng/mL LPS for 8h and subject to immunofluorescence labeling of Complex II subunit SDHA (**A**), SDHB (**B**), Complex I subunit MT-ND1, and endolysosomal protein LAMP1. Uncropped LAMP1 immunostain from Fig 2B is shown in panel S8B. **C.** Analysis overlay of cell area (white) and SDHB puncta (red). **D.** Quantification of the number of SDHB puncta per cell in iBMDM stimulated with or without 200 ng/mL LPS for 8h using CellProfiler™. Cell-level data pooled from 3 independent experiments with ~100 cells per condition and experimental averages are shown. Experimental averages are reported for the relevant main figures. Graphs are presented as the mean of n = 3 independent experiments with standard deviation (SD) error bars. P values were calculated using an unpaired T-test. *P < 0.05 and ****P < 0.0001.

Fig. S9 Effect of DRP1 KD on LPS-induced SDHB sequestration.

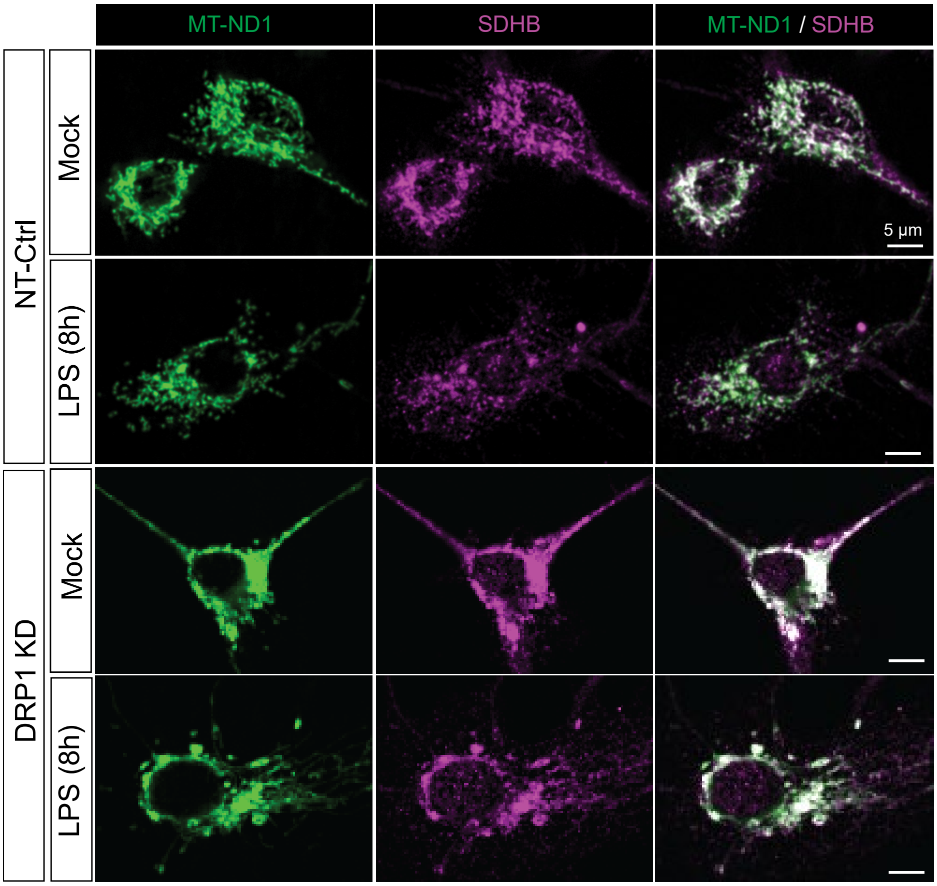

Fig. S9 Effect of DRP1 KD on LPS-induced SDHB sequestration. Representative confocal fluorescence micrographs from NT-Control and DRP1 KD iBMDM stimulated with or without 200 ng/mL LPS for 8h and subject to immunofluorescence labeling of SDHB and MT-ND1. Representative images paired to analysis in Fig. 3F. Images are representative of n=3 independent experiments.

Fig. S10 Monitoring SDHB localization and abundance in Mito-QC primary BMDM.

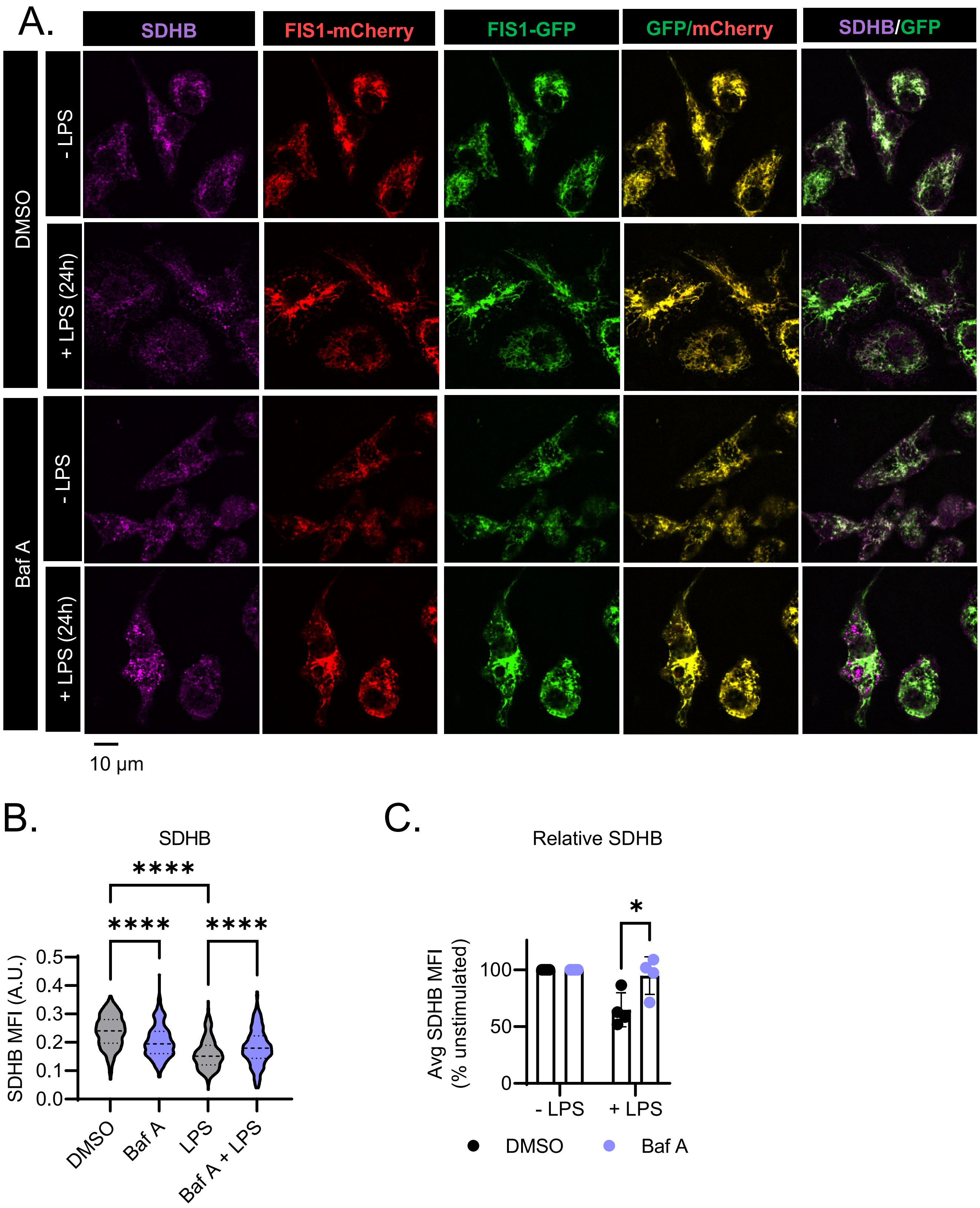

Fig. S10 Monitoring SDHB localization and abundance in Mito-QC primary BMDM. A. Representative confocal fluorescence micrographs from Mito-QC BMDM stimulated with or without 200 ng/mL LPS for 24h in the presence of 100 nM Bafilomycin A (Baf A) or vehicle control (DMSO) and subject to immunofluorescence labeling of SDHB. Representative images are paired to analysis in Fig. 3J. **B.** Cell-level quantification of the mean fluorescence intensity (MFI) of SDHB. MFI reported as arbitrary fluorescence units (A.U.). **C.** Quantification of SDHB MFI as a percentage of the intensity baseline (unstimulated) in either the Baf A or DMSO condition per experiment. Cell-level data are pooled from 4 independent experiments with ~100 cells per condition in each experiment. Graphs are presented as the mean of $n = 3$ independent experiments with standard deviation (SD) error bars. P values were calculated using a two-way ANOVA with Sidak's post-test for multiple comparisons. $*P < 0.05$ and $****P < 0.0001$.

Fig. S11 RT-qPCR analysis of Complex II subunit transcripts Sdha, Sdhb, Sdhc, and Sdhd.

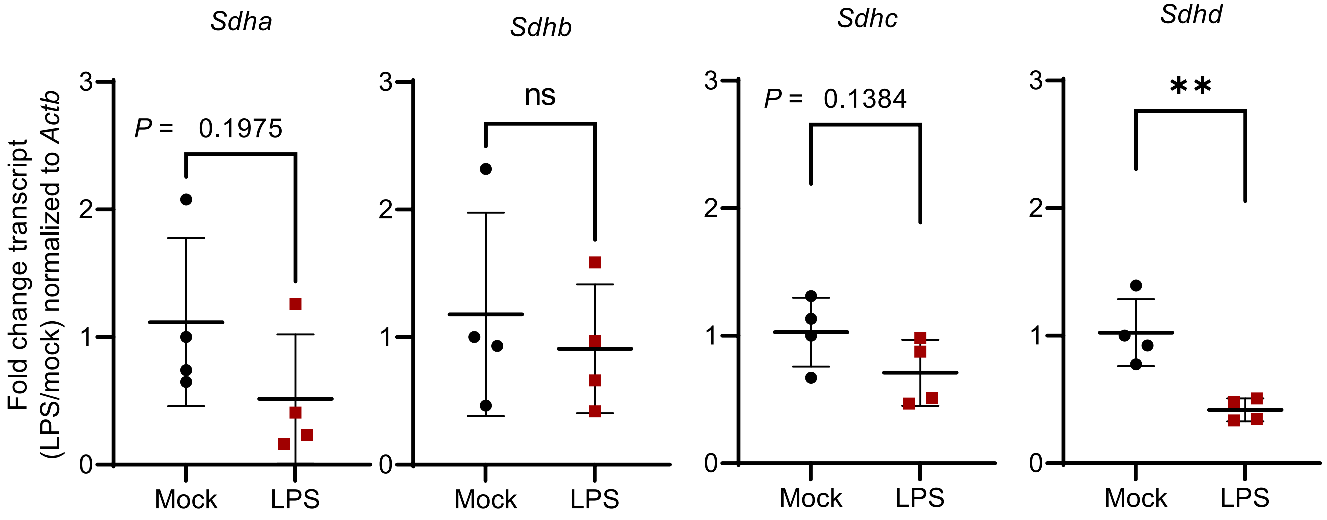

Fig. S11 RT-qPCR analysis of Complex II subunit transcripts *Sdha*, *Sdhb*, *Sdhc*, and *Sdhd*. The levels of *Sdha*, *Sdhb*, *Sdhc,* and *Sdhd* transcript were measured by RT-qPCR relative to *Actb* in 6h mock or LPS-treated iBMDM. The fold difference relative to the average mock-treated value for each transcript is reported. Graphs are presented as the mean of n = 4 independent experiments with standard deviation (SD) error bars. P values were calculated using an unpaired t-test. $^{**}P < 0.01$

Fig. S12 Validation of disruption of CL biosynthesis in CRLS1 KD iBMDM

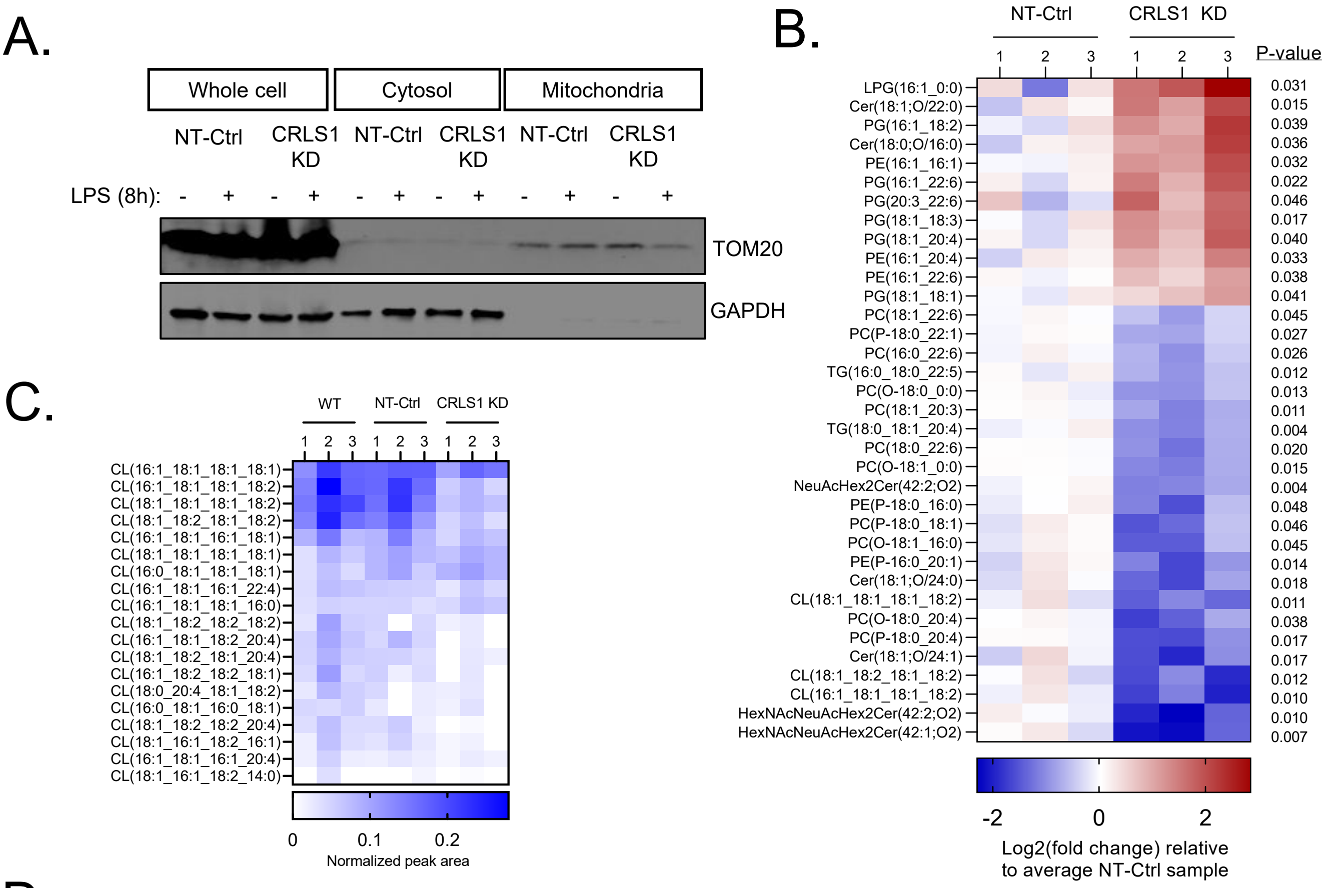

D.

Molecular Species		Lipid Class	Lipid Subclass	Category
Abbreviated	Common name			
CL (xx:x_xx:x_xx:x_xx:x)	Cardiolipin	Glycerophosphoglycerophosphoglycerols	Diacylglycerophosphoglycerophosphodiradylglycerols	Glycerophospholipids
LPG (xx:x_0:0)	Lysophosphatidylgycerol	Glycerophosphoglycerols	Monoacylglycerophosphoglycerols	Glycerophospholipids
PG (xx:x_xx:x)	Phosphatidylgycerol	Glycerophosphoglycerols	Monoacylglycerophosphomonoradylglycerols	Glycerophospholipids
PE (xx:x_xx:x)	Phosphatidylethanolamine	Glycerophosphoethanolamines	Diacylglycerophosphoethanolamines	Glycerophospholipids
PE (P-xx:x_xx:x)	Phosphatidylethanolamine	Glycerophosphoethanolamines	1-(1Z-alkenyl),2-acylglycerophosphoethanolamines	Glycerophospholipids
PC (xx:x_xx:x)	Phosphatidylcholine	Glycerophosphocholines	Diacylglycerophosphocholines	Glycerophospholipids
PC (P-xx:x_xx:x)	Phosphatidylcholine	Glycerophosphocholines	1-(1Z-alkenyl),2-acylglycerophosphocholines	Glycerophospholipids
PC (O-xx:x_xx:x)	Phosphatidylcholine	Glycerophosphocholines	1-alkyl,2-acylglycerophosphocholines	Glycerophospholipids
TG (xx:x_xx:x_xx:x)	Triacylglyceride	Triradylglycerols	Triacylglycerols	Glycerolipids
Cer (xx:x;O2/xx:x)	Ceramide	Ceramides	N-acylsphingosines (ceramides)	Sphingolipids
NeuAcHex2Cer (xx:x;O2)	Glycosphingolipids	Acidic glycosphingolipids	Gangliosides	Sphingolipids
HexNAcNeuAcHex2Cer (xx:x;O2)	Glycosphingolipids	Acidic glycosphingolipids	Gangliosides	Sphingolipids

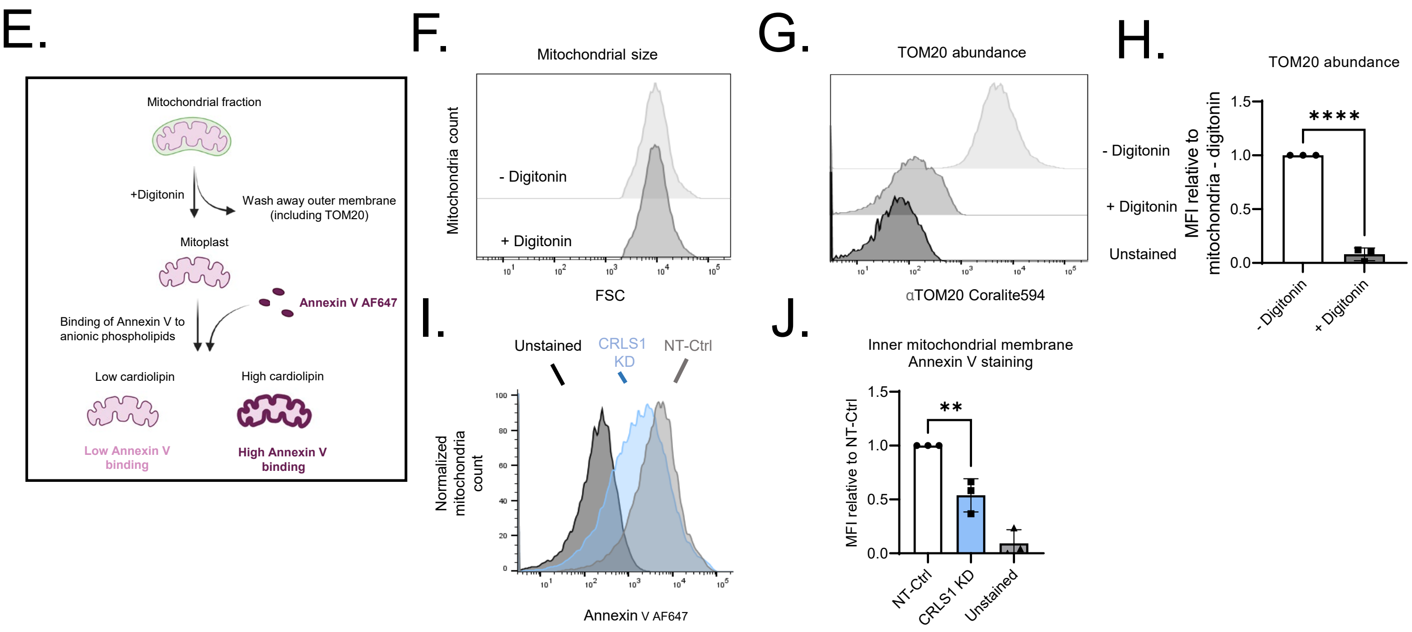

Fig. S12 Validation of disruption of CL biosynthesis in CRLS1 KD iBMDM A. Subcellular fractionation of mitochondria and immunoblot analysis of whole cell, cytosolic (GAPDH), and mitochondrial (TOM20) fractions **B.** Significantly changed lipids identified in untargeted lipidomics analysis of mitochondria isolated from CRLS1 KD and NT-Control iBMDM. Peak area is normalized to protein content per sample (Revert700 total protein stain). Lipids levels are reported as Log_2(fold change over average level in NT-Control) and sorted by fold change. **C.** All CL species detected in WT, NT-Control, and CRLS1 KD iBMDM are shown. **D.** Table with lipid nomenclature for identified molecular species. **E.** Schematic illustrating inner mitochondrial membrane Annexin V staining as a route to estimate cardiolipin levels. **F.** Flow cytometric analysis of mitochondrial size (FSC-A) +/- 0.005% digitonin-based outer membrane permeabilization. **G.** Flow cytometric analysis of mitochondrial TOM20 staining +/- digitonin. **H.** Mean fluorescence intensity (MFI) of TOM20 staining. **I.** Annexin V AF647 staining in digitonin-permeabilized mitochondria from CRLS1 KD iBMDM or NT-Control iBMDM. **J.** MFI of Annexin V AF647 staining. Graphs are presented as the mean of n = 3 independent experiments with standard deviation (SD) error bars. P values were calculated using an unpaired t-test or one-way ANOVA with Tukey's post-test for multiple comparisons. *P < 0.01 and ****P < 0.0001

Fig. S13 Flow cytometric analysis of TMRM staining in NT-Control and CRLS1 KD iBMDM.

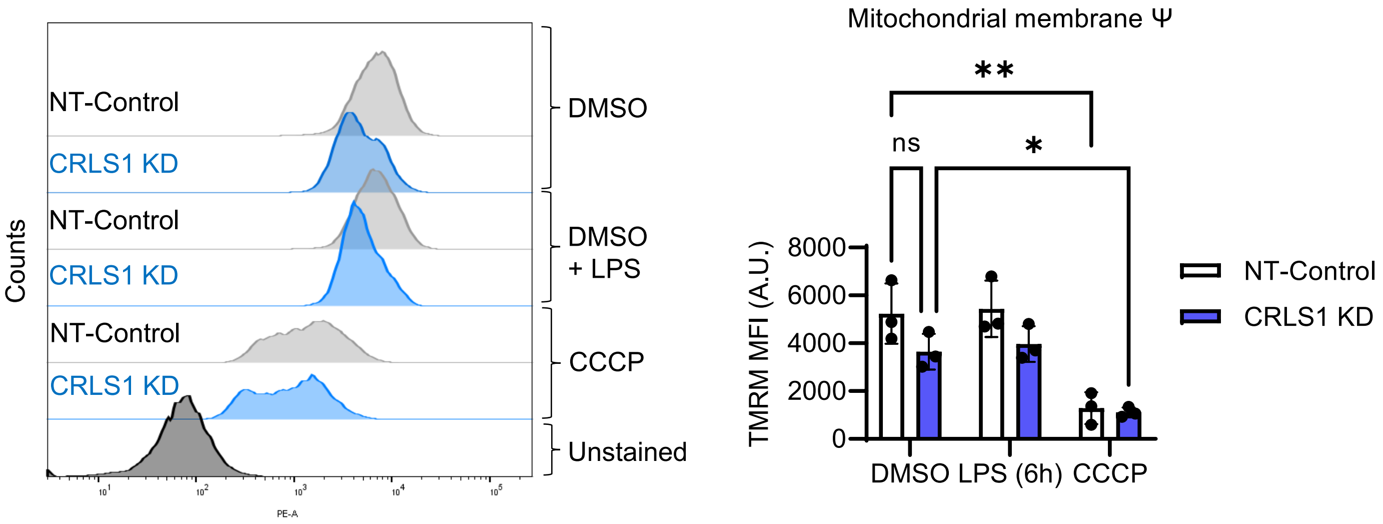

Fig. S13 Flow cytometric analysis of TMRM staining in NT-Control and CRLS1 KD iBMDM. NT-Control and CRLS1 iBMDM were stimulated +/- 200 ng/ml LPS for 6h or 20 μM of the protonophore carbonyl cyanide 3-chlorophenylhydrazone (CCCP) for 1h and then stained with the mitochondrial-membrane potential dye TMRM. DMSO was included as a vehicle control for CCCP. The geometric mean intensity of TMRM was measured by flow cytometry per experiment. Representative flow plots are shown. Graphs are presented as the mean of n = 3 independent experiments with standard deviation (SD) error bars. P values were calculated using a two-way ANOVA with Sidak's post-test for multiple comparisons. *$P < 0.05$ and **$P < 0.01$.

Fig. S14 Targeted metabolomics analysis of NT-Control and CRLS1 KD iBMDM during LPS stimulation

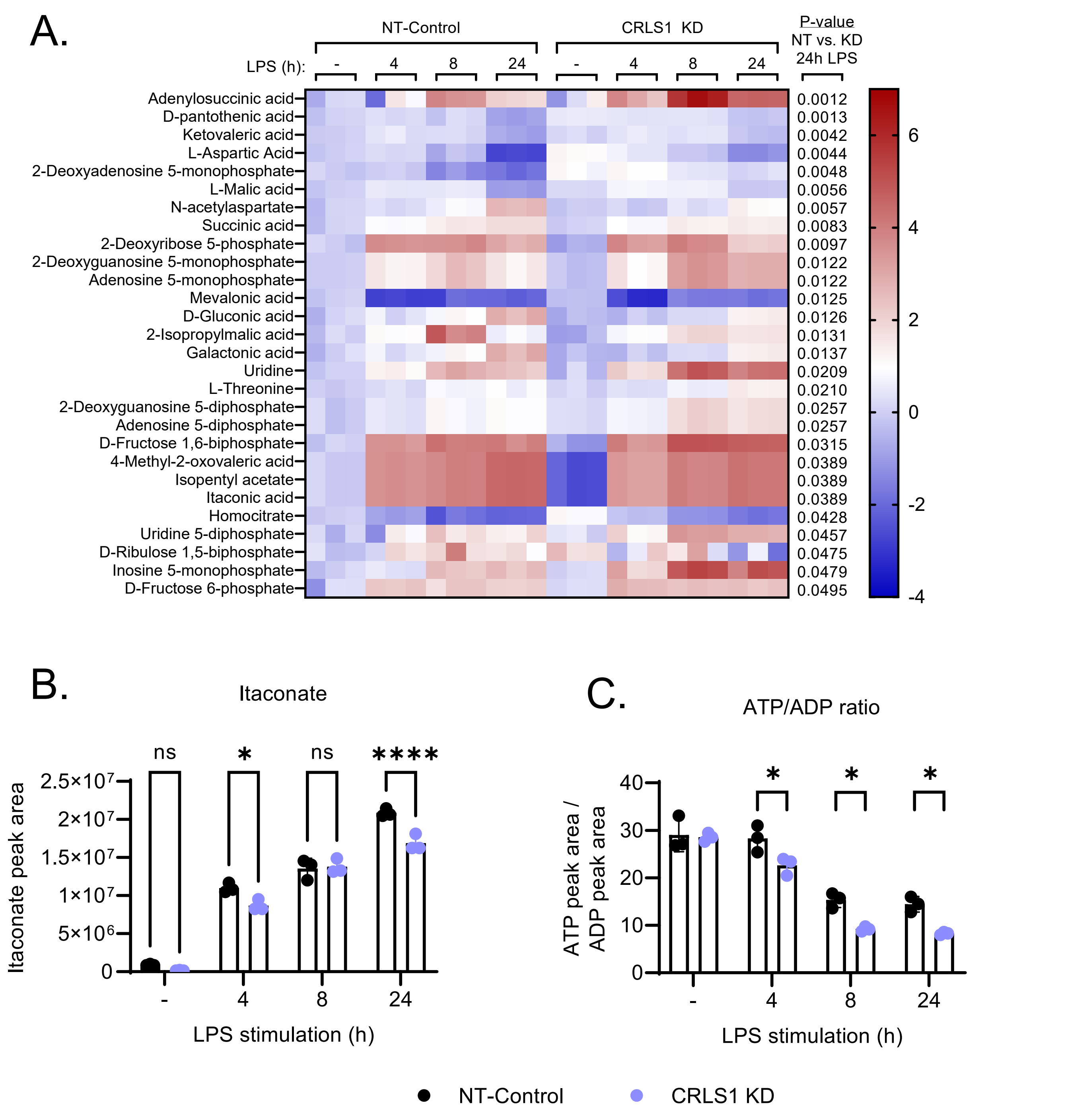

Fig. S14 Targeted metabolomics analysis of NT-Control and CRLS1 KD iBMDM during LPS stimulation. A. Significantly changed metabolites identified in targeted metabolomics analysis of whole cell extracts from CRLS1 KD and NT-Control iBMDM stimulated +/- 200 ng/ml LPS for 4, 8, or 24h. Metabolite levels are reported as Log_2(fold change over average level in unstimulated NT-Control). Metabolites are sorted by P-value following T-test comparison of 24h LPS-treated NT-Control and CRLS1 KD iBMDM. Identified TCA-related metabolites are bolded and notated with an asterisk. **B.** Highlighted analysis of Itaconate peak area. **C.** Highlighted analysis of the ratio of ATP:ADP. Graphs are presented as the mean of n = 3 independent experiments with standard deviation (SD) error bars. P values were calculated using a two-way ANOVA with Sidak's post-test for multiple comparisons. *P < 0.05 and ****P < 0.0001

Fig S15 Effect of inducible Tafazzin (TAZ) KD on LPS-stimulated SDHB loss in iBMDM

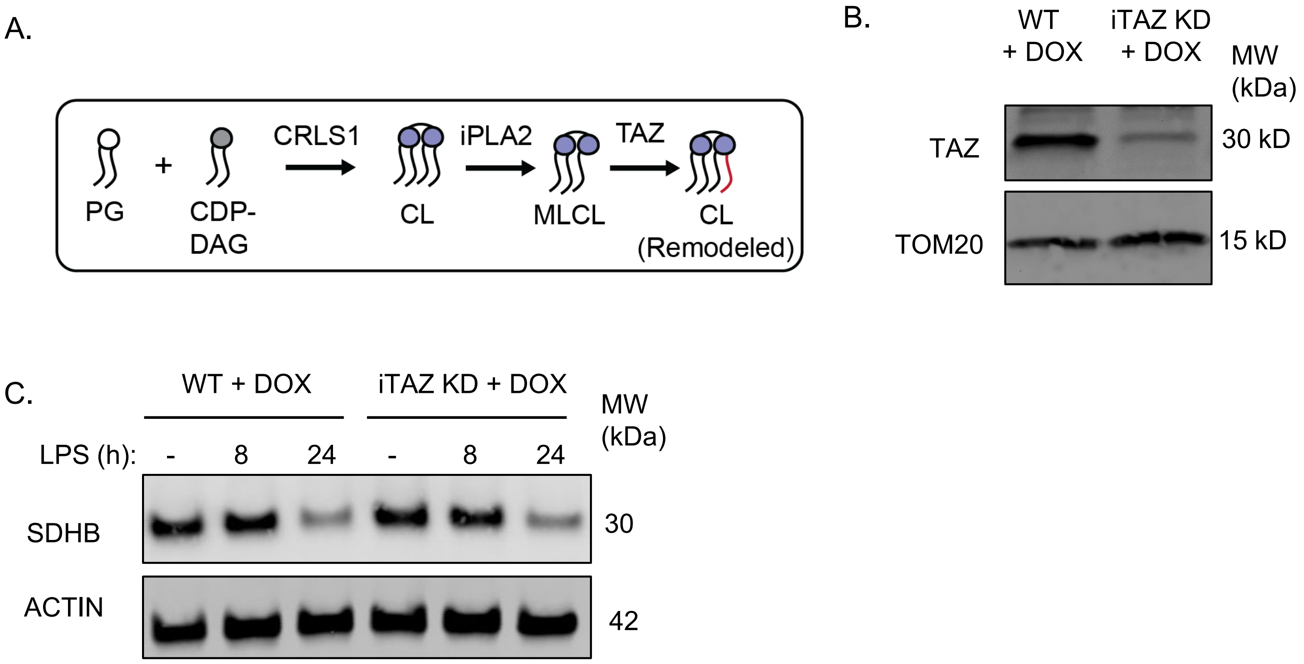

Fig. S15 Effect of inducible Tafazzin (TAZ) KD on LPS-stimulated SDHB loss in iBMDM. A. Illustration showing cardiolipin synthesis from phosphatidylglycerol (PG) and cytidine diphosphate diacylglycerol (CDP-DAG), acyl chain removal by calcium-independent phospholipase A2 enzymes (iPLA2), and acyl chain remodeling by Tafazzin (TAZ) in the mitochondria. **B.** Doxycycline-inducible TAZ KD iBMDM or iBMDM derived from WT littermates were induced with 1 µg/mL doxycycline hyclate (DOX) for 1 week and TAZ levels were measured by immunoblot. **C.** TAZ KD and WT iBMDM were synchronously stimulated with or without 200 ng/mL LPS for 8 or 24h and analyzed by SDS-PAGE and immunoblot against SDHB and ACTIN. Fig S15B is representative of n>3 independent experiments. Fig S15C is representative of n=2 independent experiments.

Fig S16. CRLS1 is required for LPS-induced iNOS expression

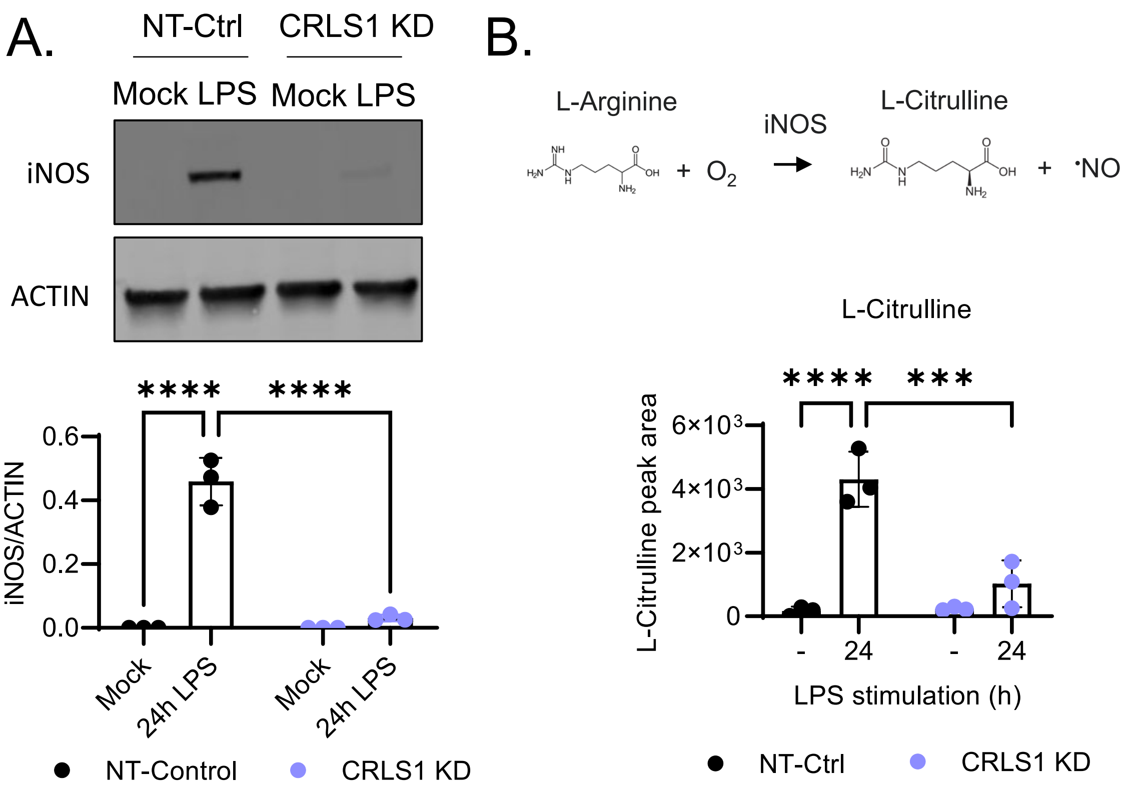

Fig. S16 CRLS1 is required for LPS-induced iNOS expression. A. CRLS1 KD and NT-Control iBMDM were stimulated with 200 ng/mL LPS for 24h and analyzed by SDS-PAGE and immunoblot against inducible nitric oxide synthase (iNOS) and ACTIN. iNOS levels relative to ACTIN in each condition are reported. **B.** Illustration of the enzymatic activity of iNOS and highlighted analysis of L-Citrulline peak area as measured in Fig S14. Graphs are presented as the mean of n = 3 independent experiments with standard deviation (SD) error bars. P values were calculated using a two-way ANOVA with Sidak's post-test for multiple comparisons. ***P < 0.001 and ****P < 0.0001

Fig. S17 Analysis of SDHB and SLP2-GFP sequestration in NT-Ctrl and CRLS1 KD iBMDM.

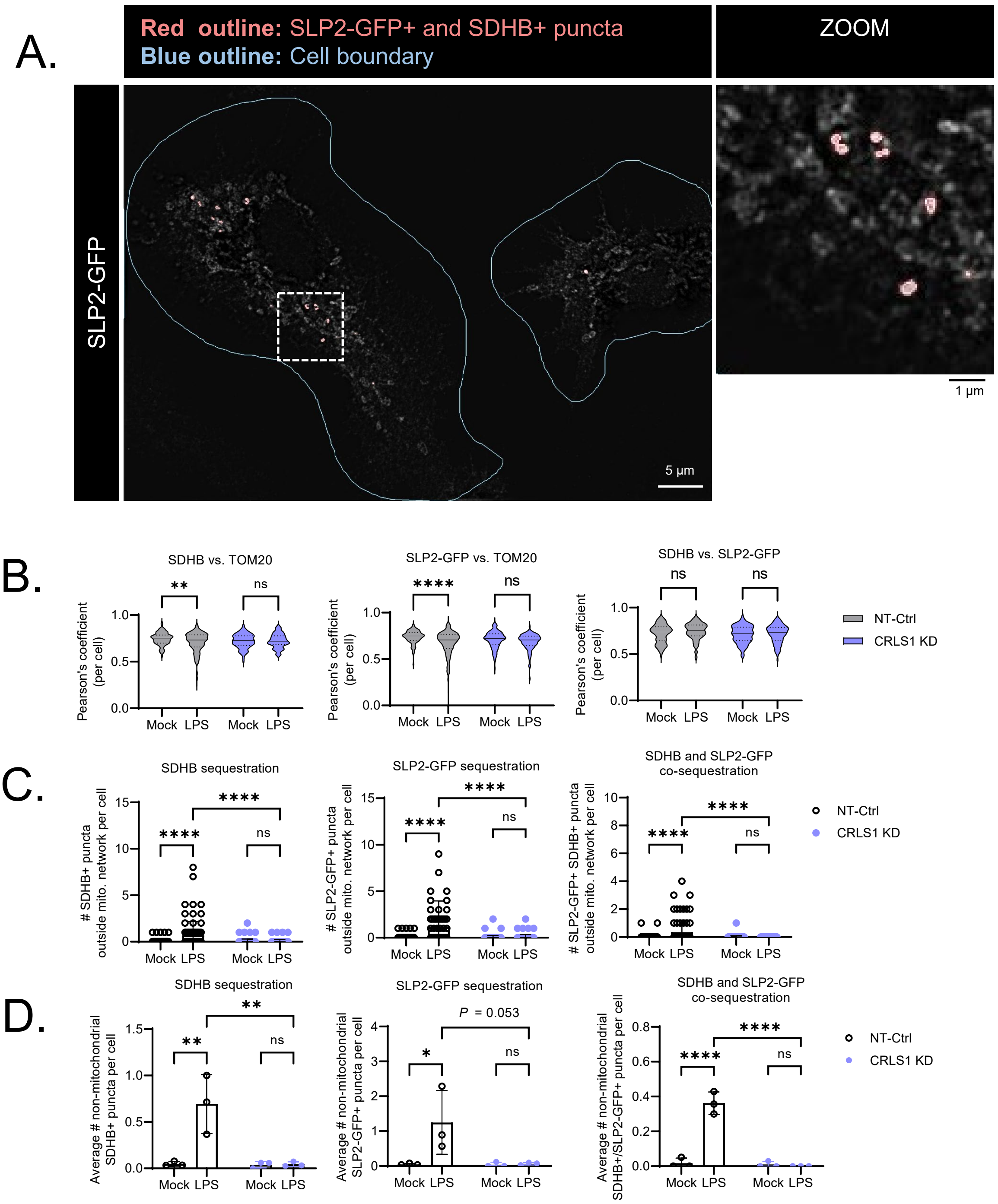

Fig. S17 Analysis of SDHB and SLP2-GFP sequestration in NT-Ctrl and CRLS1 KD iBMDM. A. CellProfiler™ analysis overlay illustrating identification of non-mitochondrial (no TOM20 overlap) SDHB and SLP2-GFP double-positive puncta presented on 2-D SIM SLP2-GFP image. **B.** Colocalization analysis (Pearson's) of total SDHB vs. TOM20, SLP2-GFP vs. TOM20, and SHDB vs. SLP2-GFP per cell. **C.** Cell-level data illustrating the number of SHB, SLP2-GFP, or SDHB and SLP2-GFP double positive puncta per cell. **D.** Experimental averages corresponding to Fig S17C. Graphs are presented as the mean of n = 3 independent experiments with standard deviation (SD) error bars. P values were calculated using a two-way ANOVA with Sidak's post-test for multiple comparisons. *$P < 0.05$; **$P < 0.01$; and ****$P < 0.0001$

Fig. S18 Cytokine production in LPS-stimulated CRLS1 KD and NT-Control iBMDM

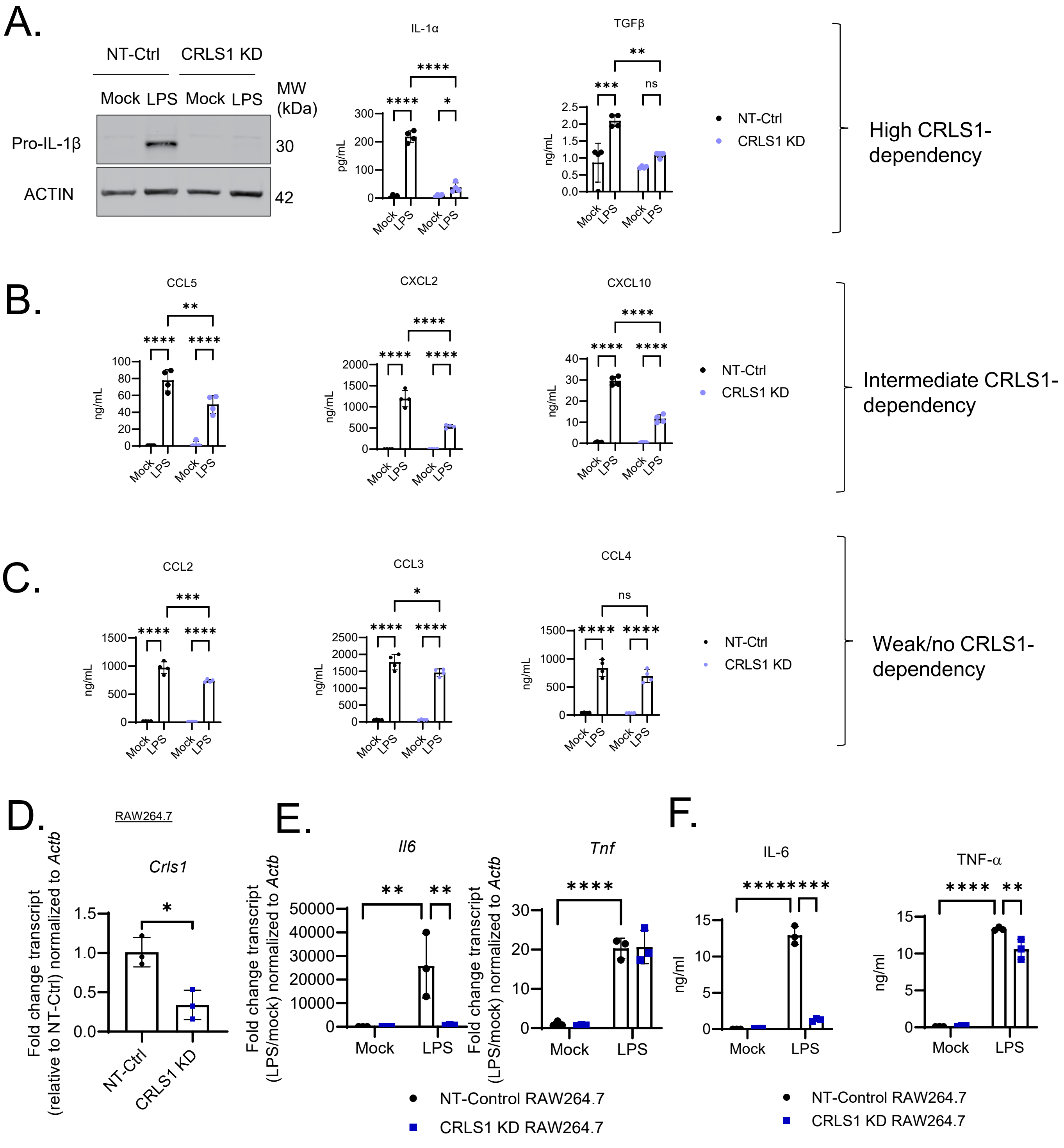

Fig. S18 Cytokine production in LPS-stimulated CRLS1 KD and NT-Control macrophages. CRLS1 KD and NT-Control iBMDM were stimulated with 200 ng/mL LPS for 24h and then supernatants were collected for secreted cytokine ELISA analysis and lysates were collected for IL-1β and ACTIN immunoblot. Cytokine analysis revealed three categories of CRLS1-dependency for LPS-induced cytokines and chemokines: highly dependent cytokines Pro-IL-1β, IL-1α, and TGFβ (**A**), moderately dependent cytokines CCL5, CXCL2, and CXCL10 (**B**), and weakly or not dependent cytokines CCL2, CCL3, and CCL4 (**C**). **D.** RAW264.7 cells were transduced with a lentiviral vector encoding an shRNA targeted to *Crls1* or a non-target control (NT-Control) sequence. *Crls1* transcript levels in CRLS1 KD and NT-Control RAW264.7 cells. **E.** Transcript levels of *Il6 and Tnf* in CRLS1 KD and NT-Control RAW264.7 cells stimulated +/- 200 ng/ml LPS for 4h. **F.** ELISA analysis of secreted IL-6 and TNF-α in CRLS1 KD and NT-Control RAW264.7 cells stimulated +/- 200 ng/ml LPS for 24h. Graphs are presented as the mean of $n \geq 3$ independent experiments with standard deviation (SD) error bars. P values were calculated using a two-way ANOVA with Sidak's post-test for multiple comparisons. *$P < 0.05$; **$P < 0.01$; ***$P < 0.001$; and ****$P < 0.0001$.

Fig. S19 Effect of CRLS1 KD on macrophage phagocytic, antimicrobial, and signaling functions during bacterial infection

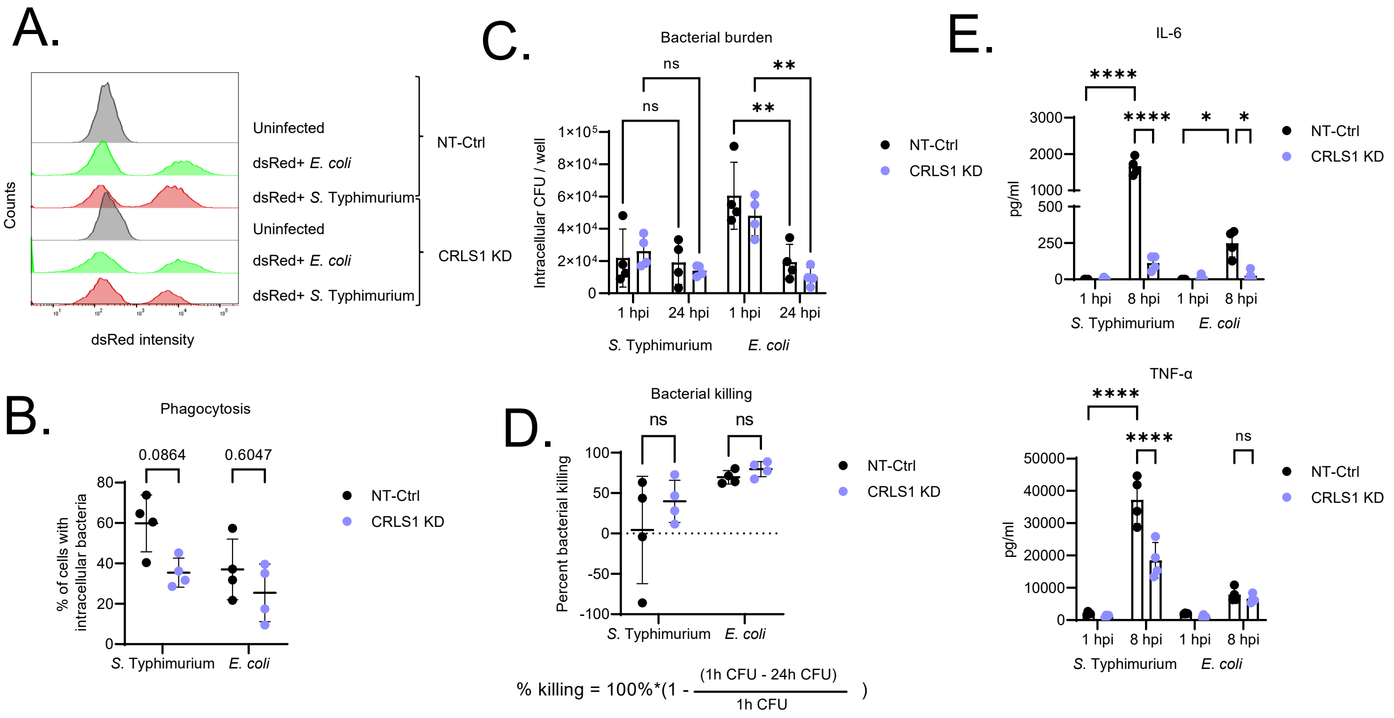

Fig. S19 Effect of CRLS1 KD on macrophage phagocytic, antimicrobial, and signaling functions during bacterial infection. A. CRLS1 KD and NT-Control iBMDM were infected with *Salmonella enterica* serovar Typhimurium (STM) (**SL1344**) or non-pathogenic *E coli* (EC) (**TOP10**) expressing dsRED at an MOI of 25 and analyzed at 1h post infection (hpi) by flow cytometry. **B.** The percentage of cells with intracellular bacteria as indicated by dsRED positivity. **C.** Intracellular bacterial colony forming units (CFU) from CRLS1 KD and NT-Control iBMDM infected with *Salmonella enterica* serovar Typhimurium (STM) (**SL1344**) or non-pathogenic *E coli* (EC) (**TOP10**) at an MOI of 25. **D.** Calculation of intracellular bacterial killing from Fig. S19C as the percentage of CFU at 1 hpi less the CFU at 24 hpi divided by the CFU at 1 hpi. **E.** ELISA analysis of IL-6 and TNF-α from STM or EC-infected iBMDM at 1 and 24 hpi. Graphs are presented as the mean of n = 4 independent experiments with standard deviation (SD) error bars. P values were calculated using a two-way ANOVA with Sidak's post-test for multiple comparisons. *P < 0.05; **P < 0.01; ***P < 0.001; and ****P < 0.0001.

Fig. S20 Pro-inflammatory cytokine production during Atpenin A5 or diethyl succinate treatment.

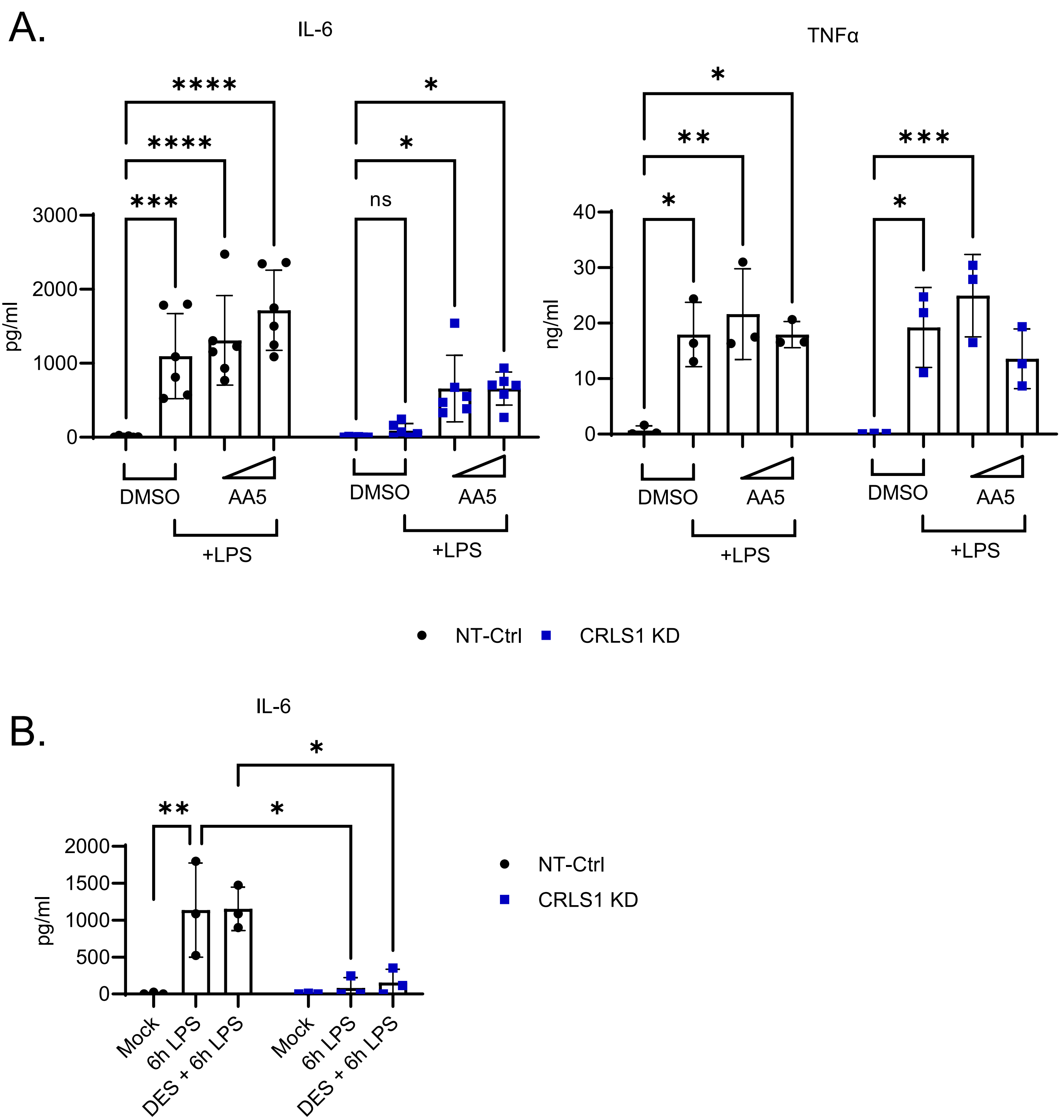

Fig. S20 Pro-inflammatory cytokine production during Atpenin A5 or diethyl succinate treatment. A. ELISA analysis of IL-6 and TNF-α secreted by NT-Control and CRLS1 KD iBMDM pretreated for 1h with or without 0.1 or 1 uM Atpenin A5 (AA5) then challenged with 200 ng/mL LPS for 6h. DMSO was included as a vehicle control for AA5. **B.** ELISA analysis of IL-6 and TNF-α secreted by NT-Control and CRLS1 KD iBMDM pretreated with 5 mM diethyl succinate (DES) for 2h and then challenged with 200 ng/mL LPS for 6 h. Supernatants were collected, and secreted IL-6 was analyzed by ELISA. Graphs are presented as the mean of $n \geq 3$ independent experiments with standard deviation (SD) error bars. P values were calculated using a two-way ANOVA with Sidak's post-test for multiple comparisons. *$P < 0.05$; **$P < 0.01$; ***$P < 0.001$; and ****$P < 0.0001$.

Fig. S21 SDHB sequestration during dimethyl malonate (DMM) treatment.

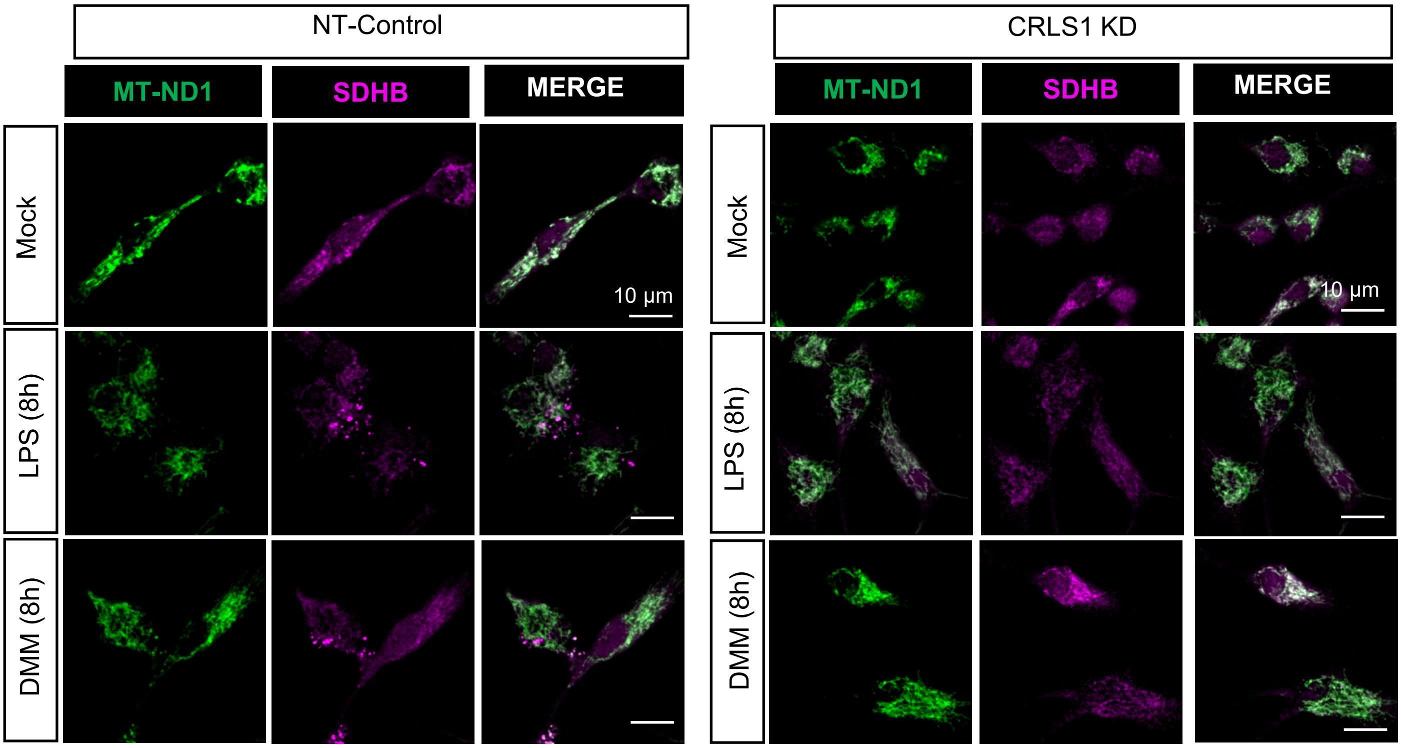

Fig. S21 SDHB sequestration during dimethyl malonate (DMM) treatment. Representative confocal fluorescence micrographs from NT-Control and CRLS1 KD iBMDM stimulated with or without 10 mM DMM or 200 ng/ml LPS for 8h and subject to immunofluorescence labeling of SDHB and MT-ND1.

Table S1. Antibodies and chemicals

Protein	Host species	Target	Conjugate?	Usage	Supplier	Cat #
IgG	Rb	Ms SDHB	no	IB, IFA	Proteintech	10620-1-AP
IgG	Ms	Ms/Hu SDHB	no	IB, IFA	Abcam	ab14714
IgG	Rb	Ms/Hu SDHA	no	IB, IFA, IP	Proteintech	4865-1-AP
IgG	Ms	Rodent OXPHOS cocktail	no	IB	Abcam	ab110413
IgG	Ms	ATP5A	no	IB	Abcam	ab14748
IgG	Rat	Ms CD3	BV421	FC	Biolegend	100213
IgG	Rat	Ms CD19	FITC	FC	Southern Biotech	1575-02
IgG	Rat	Ms CD11b	APC	FC	Southern Biotech	1561-11
IgG	Rat	Ms CD11b	BV421	FC	Biolegend	101235
IgG	Gt	Rb IgG	AF488	IFA, FC	Fisher	A11034
IgG	Gt	Rb IgG	AF594	IFA	Fisher	A11037
IgG	Gt	Rb IgG	AF647	IFA, FC	Fisher	A21244
IgG	Gt	Ms IgG	AF488	IFA	Fisher	A11029
IgG	Gt	Ms IgG	AF594	IFA	Fisher	A11032
IgG	Gt	Ms IgG	AF647	IFA	Fisher	A21235
IgG	Gt	Rb IgG	IRDye 680	IB	LICOR	926-68071
IgG	Gt	Ms IgG	IRDye 800	IB	LICOR	926-32210
IgG	Dk	Gt IgG	IRDye 800	IB	LICOR	925-32214
IgG	Dk	Ms IgG	IRDye 680	IB	LICOR	926-68022
IgG	Ms	Ms MT-ND1	no	IFA	Fisher	43-8800
IgG	Rt	Ms LAMP1	no	IFA	DSHB	1D4B
IgG	Rb	Ms DRP1	no	IB	Cell Signaling	5391S
IgG	Rb	Ms CRLS1	no	IB	Abcam	ab156882
IgG	Rb	Ms SDHC	no	IB	Abcam	ab155999
IgG	Rb	Ms SDHD	no	IB	SDHD	PIPA534387
IgG	Rb	Ms TOM20	no	IB, IFA	Proteintech	11802-1-AP
IgG	Rb	Ms TOM20	Coralite594	FC	Proteintech	CL594-11802
IgG	Ms	Ms GAPDH	no	IB	Santa Cruz	sc-32233
IgG	Ms	Ms β-ACTIN	no	IB	Fisher	ACTN05
IgG	Ms	Ms TAZ	no	IB	Steven Claypool	N/A
IgG	Gt	IL-1β	no	IB	R&D	AF-401-NA
Annexin V	N/A	anionic phospholipid	AF647	FC	Biolegend	640912

Key	
IB	Immunoblot
IFA	Immunofluorescence assay
FC	Flow cytometry
IP	Immunoprecipitation

Rb: Rabbit
Ms: Mouse
Rt: Rat
Gt: Goat
Dk: Donkey

Chemical name	Supplier	Catalog #
Lipopolysaccharide (LPS) derived from *Salmonella enterica* Typhimurium	Sigma	L2262
Bafilomycin A1	Cayman	11038
FCCP	Agilent	103010-100
Oligomycin A	Agilent	103010-100
Rotenone	Agilent	103010-100
Antimycin A	Agilent	103010-100
Atpenin A5	Abcam	ab144194
Dimethyl malonate	Sigma	136441
DETA NONOate	Cayman	82120
Digitonin	Fisher	BN2006
Doxycyline hyclate	Sigma	D9891-1G
Puromycin	Sigma	P8833
Diethyl succinate	Sigma	112402

Table S2. Primers and shRNA

Application	Target	Sequence (5'-3')
SYBR qPCR	*Sdha* Forward	CAAAAACAGACCTGCGGCTT
SYBR qPCR	*Sdha* Reverse	AGCATTGATACCTCCCTGTGC
SYBR qPCR	*Sdhb* Forward	CGTTCTCGGCAGAGTCGG
SYBR qPCR	*Sdhb* Reverse	AGAGCCACAGATGCCTTCTC
SYBR qPCR	*Sdhc* Forward	ATAGCCTTGAGTGGAGGGGTC
SYBR qPCR	*Sdhc* Reverse	GTGAGTGGTACATGAGCGGG
SYBR qPCR	*Sdhd* Forward	AGCTCTCCTACTCCGAAGCC
SYBR qPCR	*Sdhd* Reverse	AGCCTTGGAACCAGAGTGGTG
SYBR qPCR	*Crls1* Forward	GGGCTACCTGATTCTTGAAGA
SYBR qPCR	*Crls1* Reverse	GGCCCAGTTTCGAGCAATAA
SYBR qPCR	*Il6* Forward	CAAAGCCAGAGTCCTTCAGAG
SYBR qPCR	*Il6* Reverse	GTCCTTAGCCACTCCTTCTG
SYBR qPCR	*Tnf* Forward	CTTCTGTCTACTGAACTTCGGG
SYBR qPCR	*Tnf* Reverse	CAGGCTTGTCACTCGAATTTTG
SYBR qPCR	*actb* Forward	ATGGTGGGAATGGGTCAGAAGGAC
SYBR qPCR	*actb* Reverse	CTCTTTGATGTCACGCAGGATTTC
shRNA	*Drp1*	GGCAATTGAGCTAGCTATA
shRNA	*Crls1*	GAAGACTTTAATGTTGCACTA
Gibson assembly	*SLP2* AgeI forward	TAGTGAACCGTCAGATCGCACCGGTGCCACCATGCTGGCGCGCGCGGCGCGGGG
Gibson assembly	*GFP* MluI reverse	AAAACCCGGCGCGGAGGCCACGCGTTTACTTGTACAGCTCGTCCATGCCGAGAGTG

Data S1 Untargeted lipidomics dataset including normalization and data analysis relevant to this study. WT, CRLS1 KD, and NT-Control macrophages were grown overnight in DMEM supplemented with 10 mM glucose, 1 mM pyruvate, 2 mM glutamine, and 10% FBS. Mitochondria were isolated as described above, flash frozen in liquid nitrogen, and stored at −80°C. Mitochondrial isolates were quality controlled and tested for cytosolic contamination by immunoblot analysis of TOM20 and GAPDH, and total protein stains from SDS-PAGE separated mitochondrial fractions (Revert 700, LI-COR) were used for sample loading normalization for lipidomics data. Untargeted lipidomics analysis was performed after general lipid extraction using a methyl-tert-butyl ether (MTBE)-based liquid-liquid protocol. Samples were thawed at RT and 200 µL of PBS and 500 mL methanol containing 20 mL of an internal standard mixture (custom mixture from Cayman Chemical; see associated documentation) were added to each sample. Samples were vortexed, and 1000 mL methanol and 5 mL MTBE were sequentially added to each sample. After additional vortexing, the mixture was incubated on a tabletop shaker at 500 rpm at RT for 1 hour. Phase separation was induced by the addition of 1.25 mL water. Samples were sonicated for 10 minutes, then centrifuged at 2000 x g for 20 minutes. The upper organic phase of each sample was carefully removed using a Pasteur pipette and transferred into a clean glass tube. The remaining aqueous phase was re-extracted with 2.5 ml of the upper phase of MTBE/methanol/water 10:3:2.5 (v/v/v) solvent mixture, whose composition was similar to the expected composition of the upper phase. After vortexing and centrifugation as above, the organic phase was collected and combined with the initial organic phase. The extracted lipids were dried overnight in a SpeedVac concentrator. The dried lipid extracts were reconstituted in 200 µL n-butanol/methanol 1:1 (v/v) and transferred into autosampler vials for analysis by LC-MS/MS. The parameters of the LC-MS/MS settings can be found in the supplementary data files. Lipostar software (Version 2.0.2b3; Molecular Discovery) was used for feature detection, noise and artifact reduction, alignment, normalization, and lipid identification. Significantly changed lipids between CRLS1 KD and NT-Control macrophages were identified using unpaired T-tests and filtering of $P < 0.05$ and absolute value of $\log_2$(fold change) > 0.5.

Data S2 Targeted metabolomics dataset including data analysis relevant to this study. CRLS1 KD, and NT-Control macrophages were grown overnight in DMEM supplemented with 10 mM glucose, 1 mM pyruvate, 2 mM glutamine, and 10% FBS and then stimulated with or without LPS (200 ng/mL) for 4, 8, or 24h. Stimulation was synchronized such that cells across conditions were in culture for the same amount of time. After stimulation, cells were washed twice with ice cold DPBS, and metabolites were extracted by adding cold 80% methanol, incubating at -80°C for 10 min, followed by centrifugation at 17,000 x g for 10 min at 4°C. The resulting metabolite supernatant was collected. Metabolite extracts were normalized to protein content from paired samples, and the normalized fraction was dried using a SpeedVac at 4°C for 8h. Dried metabolite pellets were resuspended in a 50:50 mixture of MeOH and water. Liquid chromatography-based targeted tandem mass spectrometry (LC-MS/MS)-based metabolomics were performed and the data analyzed as previously described. In brief, samples were run on an Agilent 1290 Infinity II LC -6470 Triple Quadrupole (QqQ) tandem mass spectrometer system consisting of the 1290 Infinity II LC Flexible Pump (Quaternary Pump), the 1290 Infinity II Multisampler, the 1290 Infinity II Multicolumn Thermostat with 6 port valve and the 6470 triple quad mass spectrometer. Agilent Masshunter Workstation Software LC/MS Data Acquisition for 6400 Series Triple Quadrupole MS with Version B.08.02 was used for compound optimization, calibration, and data acquisition. Significantly changed metabolites between CRLS1 KD and NT-

Control macrophages were identified using T-tests and filtering of $P < 0.05$ and absolute value of $\log_2$(fold change) > 0.5.

Data S3: SLP2-eGFP pTRIPZ plasmid map generated in SnapGene:

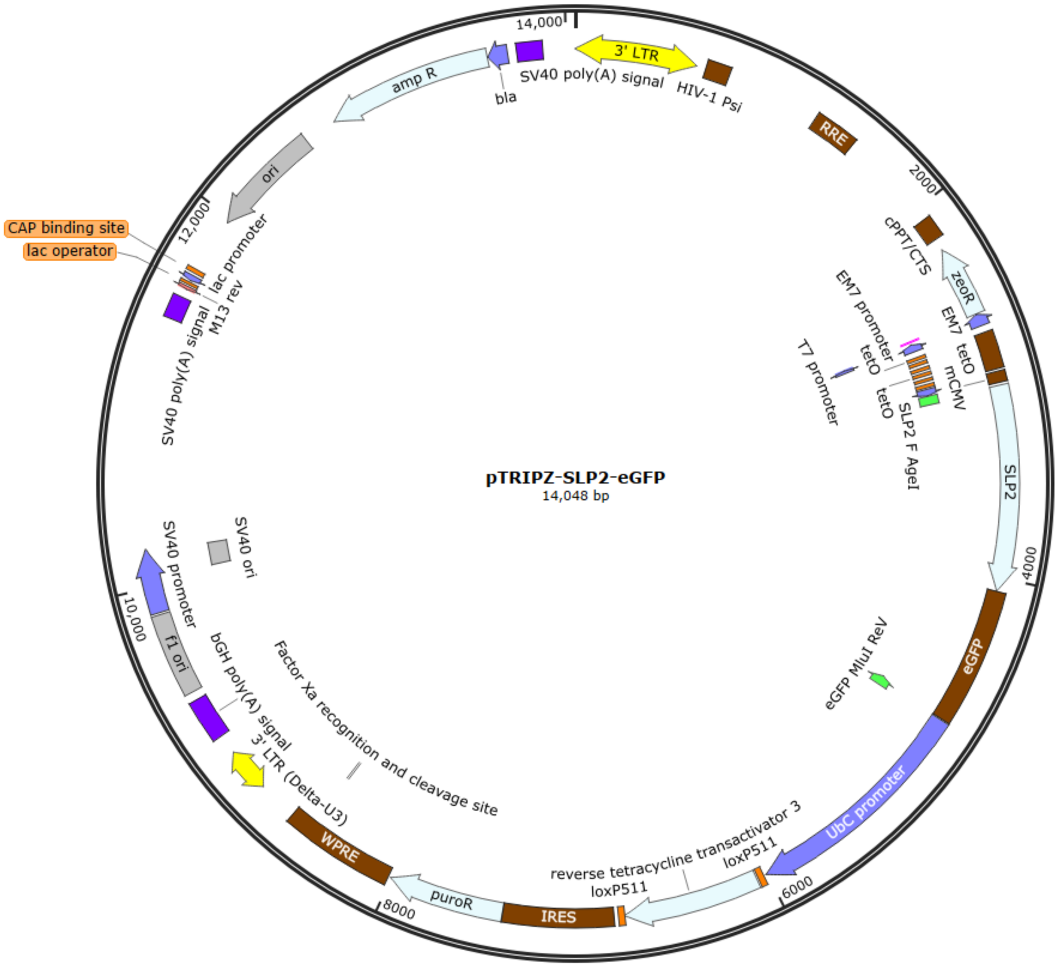

Full plasmid sequence (5’-3’):

tggaagggctaattcactcccaaagaagacaagatatccttgatctgtggatctaccacacacaaggctacttccctgattagcagaactacacacca
gggccaggggtcagatatccactgacctttggatggtgctacaagctagtaccagttgagccagataaggtagaagaggccaataaaggagagaac
accagcttgttacaccctgtgagcctgcatgggatggatgacccggagagagaagtgttagagtggaggtttgacagccgcctagcatttcatcacgt
ggcccgagagctgcatccggagtacttcaagaactgctgatatcgagcttgctacaagggactttccgctggggactttccagggaggcgtggcctgg
gcgggactggggagtggcgagccctcagatcctgcatataagcagctgctttttgcctgtactgggtctctctggttagaccagatctgagcctgggag
ctctctggctaactagggaacccactgcttaagcctcaataaagcttgccttgagtgcttcaagtagtgtgtgcccgtctgttgtgtgactctggtaacta
gagatccctcagacccttttagtcagtgtggaaaatctctagcagtggcgcccgaacagggacttgaaagcgaaagggaaaccagaggagctctctc
gacgcaggactcggcttgctgaagcgcgcacggcaagaggcgaggggcggcgactggtgagtacgccaaaaattttgactagcggaggctagaag
gagagagatgggtgcgagagcgtcagtattaagcgggggagaattagatcgcgatgggaaaaaattcggttaaggccagggggaaagaaaaaata
taaattaaaacatatagtatgggcaagcagggagctagaacgattcgcagttaatcctggcctgttagaaacatcagaaggctgtagacaaatactg

ggacagctacaaccatcccttcagacaggatcagaagaacttagatcattatataatacagtagcaaccctctattgtgtgcatcaaaggatagagat
aaaagacaccaaggaagctttagacaagatagaggaagagcaaaacaaaagtaagaccaccgcacagcaagcggccggccgctgatcttcagac
ctggaggaggagatatgagggacaattggagaagtgaattatataaatataaagtagtaaaaattgaaccattaggagtagcacccaccaaggcaa
agagaagagtggtgcagagagaaaaaagagcagtgggaataggagctttgttccttgggttcttgggagcagcaggaagcactatgggcgcagcgt
caatgacgctgacggtacaggccagacaattattgtctggtatagtgcagcagcagaacaatttgctgagggctattgaggcgcaacagcatctgttg
caactcacagtctggggcatcaagcagctccaggcaagaatcctggctgtggaaagatacctaaaggatcaacagctcctggggatttggggttgctc
tggaaaactcatttgcaccactgctgtgccttggaatgctagttggagtaataaatctctggaacagatttggaatcacacgacctggatggagtggga
cagagaaattaacaattacacaagcttaatacactccttaattgaagaatcgcaaaaccagcaagaaaagaatgaacaagaattattggaattagat
aaatgggcaagtttgtggaattggtttaacataacaaattggctgtggtatataaaattattcataatgatagtaggaggcttggtaggtttaagaata
gtttttgctgtactttctatagtgaatagagttaggcagggatattcaccattatcgtttcagacccacctcccaaccccgaggggacccgacaggcccg
aaggaatagaagaagaaggtggagagagagacagagacagatccattcgattagtgaacggatcggcactgcgtgcgccaattctgcagacaaat
ggcagtattcatccacaattttaaaagaaaaggggggattggggggtacagtgcaggggaaagaatagtagacataatagcaacagacatacaaa
ctaaagaattacaaaaacaaattacaaaaattcaaaattttcgggtttattacagggacagcagagatccagtttggttagtaccgggcccgctctagt
ccggaatcagtcctgctcctcggccacgaagtgcacgcagttgccggccgggtcgcgcagggcgaactcccgcccccacggctgctcgccgatctcgg
tcatggccggcccggaggcgtcccggaagttcgtggacacgacctccgaccactcggcgtacagctcgtccaggccgcgcacccacacccaggccag
ggtgttgtccggcaccacctggtcctggaccgcgctgatgaacagggtcacgtcgtcccggaccacaccggcgaagtcgtcctccacgaagtcccggg
agaacccgagccggtcggtccagaactcgaccgctccggcgacgtcgcgcgcggtgagcaccggaacggcactggtcaacttggccatggtggccct
cctatagtgagtcgtattatactatgccgatatactatgccgatgattaattgtcaacacgtgctgcaggtccgaggttctagacgagtttactccctatc
agtgatagagaacgatgtcgagtttactccctatcagtgatagagaacgtatgtcgagtttactccctatcagtgatagagaacgtatgtcgagtttact
ccctatcagtgatagagaacgtatgtcgagtttatccctatcagtgatagagaacgtatgtcgagtttactccctatcagtgatagagaacgtatgtcga
ggtaggcgtgtacggtgggaggcctatataagcagagctcgtttagtgaaccgtcagatcgcaccggtGCCACCatgctggcgcgcgcggcgcggg
gcactggggccctttgctgaggggctctctactggcttctggccgcgctccgcgccgcgcctcctctggattgccccgaaacaccgtggtactgttcgtg
ccgcagcaggaggcctgggtggtggagcgaatgggccgattccaccggatcctggagcctggtttgaacatcctcatccctgtgttagaccggatccg
atatgtgcagagtctcaaggaaattgtcatcaacgtgcctgagcagtcggctgtgactctcgacaatgtaactctgcaaatcgatggagtcctttacctg
cgcatcatggacccttacaaggcaagctacggtgtggaggaccctgagtatgccgtcacccagctagctcaaacaaccatgagatcagagctcggca
aactctctctggacaaagtcttccgggaacgggagtccctgaatgccagcattgtggatgccatcaaccaagctgctgactgctggggtatccgctgcc
tccgttatgagatcaaggatatccatgtgccaccccgggtgaaagagtctatgcagatgcaggtggaggcagagcggcggaaacgggccacagttct
agagtctgaggggacccgagagtcggccatcaatgtggcagaagggaagaaacaggcccagatcctggcctccgaagcagaaaaggctgaacag
ataaatcaggcagcaggagaggccagtgcagttctggcgaaggccaaggctaaagctgaagctattcgaatcctggctgcagctctgacacaacata
atggagatgcagcagcttcactgactgtggccgagcagtatgtcagcgcgttctccaaactggccaaggactccaacactatcctactgccctccaacc
ctggcgatgtcaccagcatggtggctcaggccatgggtgtatatggagccctcaccaaagccccagtgccagggactccagactcactctccagtggg
agcagcagagatgtccagggtacagatgcaagtcttgatgaggaacttgatcgagtcaagatgagtatggtgagcaagggcgaggagctgttcacc
ggggtggtgcccatcctggtcgagctggacggcgacgtaaacggccacaagttcagcgtgtccggcgagggcgagggcgatgccacctacggcaag
ctgaccctgaagttcatctgcaccaccggcaagctgcccgtgccctggcccaccctcgtgaccaccctgacctacggcgtgcagtgcttcagccgctac
cccgaccacatgaagcagcacgacttcttcaagtccgccatgcccgaaggctacgtccaggagcgcaccatcttcttcaaggacgacggcaactaca
agacccgcgccgaggtgaagttcgagggcgacaccctggtgaaccgcatcgagctgaagggcatcgacttcaaggaggacggcaacatcctggggc
acaagctggagtacaactacaacagccacaacgtctatatcatggccgacaagcagaagaacggcatcaaggtgaacttcaagatccgccacaaca
tcgaggacggcagcgtgcagctcgccgaccactaccagcagaacacccccatcggcgacggccccgtgctgctgcccgacaaccactacctgagcac
ccagtccgccctgagcaaagaccccaacgagaagcgcgatcacatggtcctgctggagttcgtgaccgccgccgggatcactctcggcatggacgag
ctgtacaagtaaacgcgtggcctccgcgccgggttttggcgcctcccgcgggcgcccccctcctcacggcgagcgctgccacgtcagacgaagggcgc
agcgagcgtcctgatccttccgcccggacgctcaggacagcggcccgctgctcataagactcggccttagaaccccagtatcagcagaaggacatttt
aggacgggacttgggtgactctagggcactggttttctttccagagagcggaacaggcgaggaaaagtagtcccttctcggcgattctgcggagggat
ctccgtggggcggtgaacgccgatgattatataaggacgcgccgggtgtggcacagctagttccgtcgcagccgggatttgggtcgcggttcttgtttg
tggatcgctgtgatcgtcacttggtgagtagcgggctgctgggctggccggggctttcgtggccgccgggccgctcggtgggacggaagcgtgtggag

agaccgccaagggctgtagtctgggtccgcgagcaaggttgccctgaactgggggttgggggggagcgcagcaaaatggcggctgttcccgagtcttg
aatggaagacgcttgtgaggcgggctgtgaggtcgttgaaacaaggtgggggggcatggtgggcggcaagaacccaaggtcttgaggccttcgctaat
gcgggaaagctcttattcgggtgagatgggctggggcaccatctggggaccctgacgtgaagtttgtcactgactggagaactcggtttgtcgtctgtt
gcgggggcggcagttatggcggtgccgttgggcagtgcacccgtacctttgggagcgcgcgccctcgtcgtgtcgtgacgtcacccgttctgttggctta
taatgcagggtggggccacctgccggtaggtgtgcggtaggcttttctccgtcgcaggacgcagggttcgggcctagggtaggctctcctgaatcgaca
ggcgccggacctctggtgaggggagggataagtgaggcgtcagtttctttggtcggttttatgtacctatcttcttaagtagctgaagctccggttttgaa
ctatgcgctcggggttggcgagtgtgttttgtgaagtttttaggcaccttttgaaatgtaatcatttgggtcaatatgtaattttcagtgttagactagtaa
attgtccgctaaattctggccgtttttggctttttgttagacgctagcggatccataacttcgtatagtatacattatacgaagttatgccaccatgtctag
gctggacaagagcaaagtcataaacggagctctggaattactcaatggtgtcggtatcgaaggcctgacgacaaggaaactcgctcaaaagctggg
agttgagcagcctaccctgtactggcacgtgaagaacaagcgggccctgctcgatgccctgccaatcgagatgctggacaggcatcatacccacttct
gccccctggaaggcgagtcatggcaagactttctgcggaacaacgccaagtcataccgctgtgctctcctctcacatcgcgacggggctaaagtgcat
ctcggcacccgcccaacagagaaacagtacgaaaccctggaaaatcagctcgcgttcctgtgtcagcaaggcttctccctggagaacgcactgtacg
ctctgtccgccgtgggccactttacactgggctgcgtattggaggaacaggagcatcaagtagcaaaagaggaaagagagacacctaccaccgattc
tatgcccccacttctgagacaagcaattgagctgttcgaccggcagggagccgaacctgccttccttttcggcctggaactaatcatatgtggcctgga
gaaacagctaaagtgcgaaagcggcgggccgaccgacgcccttgacgattttgacttagacatgctcccagccgatgcccttgacgattttgaccttg
acatgctccccgggtaaataacttcgtatagtatacattatacgaagttatggatccgcggccgcaaattccgcccctctccctcccccccccctaacgt
tactggccgaagccgcttggaataaggccggtgtgcgtttgtctatatgttattttccaccatattgccgtcttttggcaatgtgagggcccggaaacctg
gccctgtcttcttgacgagcattcctaggggtctttcccctctcgccaaaggaatgcaaggtctgttgaatgtcgtgaaggaagcagttcctctggaagc
ttcttgaagacaaacaacgtctgtagcgaccctttgcaggcagcggaaccccccacctggcgacaggtgcctctgcggccaaaagccacgtgtataag
atacacctgcaaaggcggcacaaccccagtgccacgttgtgagttggatagttgtggaaagagtcaaatggctctcctcaagcgtattcaacaaggg
gctgaaggatgcccagaaggtaccccattgtatgggatctgatctggggcctcggtgcacatgctttacatgtgtttagtcgaggttaaaaaaacgtct
aggccccccgaaccacggggacgtggttttcctttgaaaaacacgataataccatggccaccgagtacaagcccacggtgcgcctcgccacccgcga
cgacgtccccgggccgtacgcaccctcgccgccgcgttcgccgactaccccgccacgcgccacaccgtcgacccggaccgccacatcgagcgggtc
accgagctgcaagaactcttcctcacgcgcgtcgggctcgacatcggcaaggtgtgggtcgcggacgacggcgccgcggtggcggtctggaccacgc
cggagagcgtcgaagcgggggcggtgttcgccgagatcggctcgcgcatggccgagttgagcggttcccggctggccgcgcagcaacagatggaag
gcctcctggcgccgcaccggcccaaggagcccgcgtggttcctggccaccgtcggcgtctcgcccgaccaccagggcaagggtctgggcagcgccgt
cgtgctccccggagtggaggcggccgagcgcgctggggtgcccgccttcctggagacctccgcgccccgcaacctccccttctacgagcggctcggctt
caccgtcaccgccgacgtcgaggtgcccgaaggaccgcgcacctggtgcatgacccgcaagcccggtgcctgagttcgcgtctggaacaatcaacct
ctggattacaaaatttgtgaaagattgactggtattcttaactatgttgctccttttacgctatgtggatacgctgctttaatgcctttgtatcatgctattg
cttcccgtatggctttcattttctcctccttgtataaatcctggttgctgtctctttatgaggagttgtggcccgttgtcaggcaacgtggcgtggtgtgcac
tgtgtttgctgacgcaacccccactggttggggcattgccaccacctgtcagctcctttccgggactttcgctttccccctccctattgccacggcggaact
catcgccgcctgccttgcccgctgctggacaggggctcggctgttgggcactgacaattccgtggtgttgtcggggaagctgacgtcctttccatggctg
ctcgcctgtgttgccacctggattctgcgcgggacgtccttctgctacgtcccttcggccctcaatccagcggaccttccttcccgcggcctgctgccggct
ctgcggcctcttccgcgtcttcgccttcgccctcagacgagtcggatctccctttgggccgcctccccgcctggaattaattctgcagtcgagacctagaa
aaacatggagcaatcacaagtagcaatacagcagctaccaatgctgattgtgcctggctagaagcacaagaggaggaggaggtgggttttccagtc
acacctcaggtacctttaagaccaatgacttacaaggcagctgtagatcttagccactttttaaaagaaaagaggggactggaagggctaattcactc
ccaacgaagacaagatctgctttttgcttgtactgggtctctctggttagaccagatctgagcctgggagctctctggctaactagggaacccactgctt
aagcctcaataaagcttgccttgagtgcttcaagtagtgtgtgcccgtctgttgtgtgactctggtaactagagatccctcagacccttttagtcagtgtg
gaaaatctctagcagtagtagttcatgtcatcttattattcagtatttataacttgcaaagaaatgaatatcagagagtgagaggccttgacattgttta
aacccgctgatcagcctcgactgtgccttctagttgccagccatctgttgtttgcccctcccccgtgccttccttgaccctggaaggtgccactcccactgt
cctttcctaataaaatgaggaaattgcatcgcattgtctgagtaggtgtcattctattctggggggtggggtggggcaggacagcaagggggaggatt
gggaagacaatagcaggcatgctggggatgcggtgggctctatggcttctgaggcggaaagaaccagctggggctctagggggtatccccacgcgc
cctgtagcggcgcattaagcgcggcgggtgtggtggttacgcgcagcgtgaccgctacacttgccagcgccctagcgcccgctcctttcgctttcttccc
ttcctttctcgccacgttcgccggctttccccgtcaagctctaaatcgggggctccctttagggttccgatttagtgctttacggcacctcgaccccaaaaa

acttgattagggtgatggttcacgtagtgggccatcgccctgatagacggtttttcgccctttgacgttggagtccacgttctttaatagtggactcttgtt
ccaaactggaacaacactcaaccctatctcggtctattcttttgatttataagggattttgccgatttcggcctattggttaaaaaatgagctgatttaac
aaaaatttaacgcgaattaattctgtggaatgtgtgtcagttagggtgtggaaagtccccaggctccccagcaggcagaagtatgcaaagcatgcatc
tcaattagtcagcaaccaggtgtggaaagtccccaggctccccagcaggcagaagtatgcaaagcatgcatctcaattagtcagcaaccatagtccc
gcccctaactccgcccatcccgcccctaactccgcccagttccgcccattctccgccccatggctgactaattttttttatttatgcagaggccgaggccg
cctctgcctctgagctattccagaagtagtgaggaggcttttttggaggcctaggcttttgcaaaaagctcccgggagcttgtatatccattttcggatct
gatcagcacgtgatgaaaaagcctgaactcaccgcgacgtctgtcgagaagtttctgatcgaaaagttcgacagcgtctccgacctgatgcagctctc
ggagggcgaagaatctcgtgctttcagcttcgatgtaggagggcgtggatatgtcctgcgggtaaatagctgcgccgatggtttctacaaagatcgtta
tgtttatcggcactttgcatcggccgcgctcccgattccggaagtgcttgacattggggaattaattcagcgagagcctgacctattgcatctcccgccgt
gcacagggtgtcacgttgcaagacctgcctgaaaccgaactgcccgctgttctgcagccggtcgcggaggccatggatgcgatcgctgcggccgatct
tagccagacgagcgggttcggcccattcggaccgcaaggaatcggtcaatacactacatggcgtgatttcatatgcgcgattgctgatccccatgtgta
tcactggcaaactgtgatggacgacaccgtcagtgcgtccgtcgcgcaggctctcgatgagctgatgctttgggccgaggactgccccgaagtccggc
acctcgtgcacgcggatttcggctccaacaatgtcctgacggacaatggccgcataacagcggtcattgactggagcgaggcgatgttcggggattcc
caatacgaggtcgccaacatcttcttctggaggccgtggttggcttgtatggagcagcagacgcgctacttcgagcggaggcatccggagcttgcagg
atcgccgcggctccgggcgtatatgctccgcattggtcttgaccaactctatcagagcttggttgacggcaatttcgatgatgcagcttgggcgcagggt
cgatgcgacgcaatcgtccgatccggagccgggactgtcgggcgtacacaaatcgcccgcagaagcgcggccgtctggaccgatggctgtgtagaag
tactcgccgatagtggaaaccgacgccccagcactcgtccgagggcaaaggaatagcacgtgctacgagatttcgattccaccgccgccttctatgaa
aggttgggcttcggaatcgttttccgggacgccggctggatgatcctccagcgcggggatctcatgctggagttcttcgcccaccccaacttgtttattgc
agcttataatggttacaaataaagcaatagcatcacaaatttcacaaataaagcatttttttcactgcattctagttgtggtttgtccaaactcatcaatg
tatcttatcatgtctgtataccgtcgacctctagctagagcttggcgtaatcatggtcatagctgtttcctgtgtgaaattgttatccgctcacaattccac
acaacatacgagccggaagcataaagtgtaaagcctggggtgcctaatgagtgagctaactcacattaattgcgttgcgctcactgcccgctttccagt
cgggaaacctgtcgtgccagctgcattaatgaatcggccaacgcgcggggagaggcggtttgcgtattgggcgctcttccgcttcctcgctcactgact
cgctgcgctcggtcgttcggctgcggcgagcggtatcagctcactcaaaggcggtaatacggttatccacagaatcaggggataacgcaggaaagaa
catgtgagcaaaaggccagcaaaaggccaggaaccgtaaaaaggccgcgttgctggcgtttttccataggctccgcccccctgacgagcatcacaaa
aatcgacgctcaagtcagaggtggcgaaacccgacaggactataaagataccaggcgtttccccctggaagctccctcgtgcgctctcctgttccgac
cctgccgcttaccggatacctgtccgcctttctcccttcgggaagcgtggcgctttctcatagctcacgctgtaggtatctcagttcggtgtaggtcgttcg
ctccaagctgggctgtgtgcacgaaccccccgttcagcccgaccgctgcgccttatccggtaactatcgtcttgagtccaacccggtaagacacgactt
atcgccactggcagcagccactggtaacaggattagcagagcgaggtatgtaggcggtgctacagagttcttgaagtggtggcctaactacggctac
actagaagaacagtatttggtatctgcgctctgctgaagccagttaccttcggaaaaagagttggtagctcttgatccggcaaacaaaccaccgctggt
agcggtggtttttttgtttgcaagcagcagattacgcgcagaaaaaaaggatctcaagaagatcctttgatcttttctacggggtctgacgctcagtgga
acgaaaactcacgttaagggattttggtcatgagattatcaaaaaggatcttcacctagatccttttaaattaaaaatgaagttttaaatcaatctaaag
tatatatgagtaaacttggtctgacagttaccaatgcttaatcagtgaggcacctatctcagcgatctgtctatttcgttcatccatagttgcctgactccc
cgtcgtgtagataactacgatacgggagggcttaccatctggccccagtgctgcaatgataccgcgagacccacgctcaccggctccagatttatcag
caataaaccagccagccggaagggccgagcgcagaagtggtcctgcaactttatccgcctccatccagtctattaattgttgccgggaagctagagta
agtagttcgccagttaatagtttgcgcaacgttgttgccattgctacaggcatcgtggtgtcacgctcgtcgtttggtatggcttcattcagctccggttcc
caacgatcaaggcgagttacatgatcccccatgttgtgcaaaaaagcggttagctccttcggtcctccgatcgttgtcagaagtaagttggccgcagtg
ttatcactcatggttatggcagcactgcataattctcttactgtcatgccatccgtaagatgcttttctgtgactggtgagtactcaaccaagtcattctga
gaatagtgtatgcggcgaccgagttgctcttgcccggcgtcaatacgggataataccgcgccacatagcagaactttaaaagtgctcatcattggaaa
acgttcttcggggcgaaaactctcaaggatcttaccgctgttgagatccagttcgatgtaacccactcgtgcacccaactgatcttcagcatcttttactt
tcaccagcgtttctgggtgagcaaaaacaggaaggcaaaatgccgcaaaaaagggaataagggcgacacggaaatgttgaatactcatactcttcc
tttttcaatattattgaagcatttatcagggttattgtctcatgagcggatacatatttgaatgtatttagaaaaataaacaaataggggttccgcgcaca
tttccccgaaaagtgccacctgacgtcgacggatcgggagatcaacttgtttattgcagcttataatggttacaaataaagcaatagcatcacaaattt
cacaaataaagcatttttttcactgcattctagttgtggtttgtccaaactcatcaatgtatcttatcatgtctggatcaactggataactcaagctaacca

aaatcatcccaaacttcccaccccataccctattaccactgccaattacctgtggtttcatttactctaaacctgtgattcctctgaattattttcattttaa
agaaattgtatttgttaaatatgtactacaaacttagtagt

Data S3: SLP2-GFP pTRIPZ plasmid map and full sequence. The sequence corresponding to SLP2-GFP was subcloned into the pTRIPZ lentiviral vector by the University of Michigan Vector Core and was sequence validated using Eurofin DNA sequencing services. Snapgene Viewer was used to generate the annotated plasmid map, and the full sequence is provided.

File S1 CellProfiler image analysis pipeline 1: Confocal analysis of MitoSOX intensity per cell. Annotated CellProfiler image analysis pipeline designed to segment cellular objects and measure the pixel intensity of MitoSOX dye within cellular objects.

File S2 CellProfiler image analysis pipeline 2: Confocal analysis of SDHB puncta per cell. Annotated CellProfiler image analysis pipeline designed to segment and relate cellular objects and high intensity puncta of SDHB immunofluorescent signal.

File S3 CellProfiler image analysis pipeline 3: Confocal analysis of MitoQC reporter. Annotated CellProfiler image analysis pipeline designed to segment cellular objects and measure the pixel intensity and colocalization of mCherry and GFP within cellular objects.

File S4 CellProfiler image analysis pipeline 4: SIM analysis of SDHB and SLP2-GFP puncta. Annotated CellProfiler image analysis pipeline designed to segment and relate cellular objects and high intensity puncta of SDHB immunofluorescent signal or SLP2-GFP fluorescent signal. SDHB and SLP2-GFP puncta are filtered for overlap with mitochondrial network objects defined by TOM20 and related to one another to define double-positive puncta.
